# Mutual regulation between OGT and XIAP to control colon cancer cell growth and invasion

**DOI:** 10.1038/s41419-020-02999-5

**Published:** 2020-09-29

**Authors:** Hyeon Gyu Seo, Han Byeol Kim, Ji Young Yoon, Tae Hyun Kweon, Yun Soo Park, Jingu Kang, Jinwoo Jung, SeongJin Son, Eugene C. Yi, Tae Ho Lee, Won Ho Yang, Jin Won Cho

**Affiliations:** 1 Glycosylation Network Research Center, Yonsei University, 50 Yonsei-ro, Seodaemun-gu, Seoul, 03722 Republic of Korea; 2 Interdisciplinary Program of Integrated OMICS for Biomedical Science, Graduate School, Yonsei University, 50 Yonsei-ro, Seodaemun-gu, Seoul, 03722 Republic of Korea; 3 Department of Systems Biology, College of Life Science and Biotechnology, Yonsei University, 50 Yonsei-ro, Seodaemun-gu, Seoul, 03722 Republic of Korea; 4 Department of Molecular Medicine and Biopharmaceutical Sciences, School of Convergence Science and Technology and College of Medicine or College of Pharmacy, Seoul National University, 28 Yeongeon-dong, Jongno-gu, Seoul, 03080 Republic of Korea

**Keywords:** Glycobiology, Colon cancer, Glycosylation, Ubiquitin ligases

## Abstract

O-GlcNAc transferase (OGT) is an enzyme that catalyzes the O-GlcNAc modification of nucleocytoplasmic proteins and is highly expressed in many types of cancer. However, the mechanism regulating its expression in cancer cells is not well understood. This study shows that OGT is a substrate of the E3 ubiquitin ligase X-linked inhibitor of apoptosis (XIAP) which plays an important role in cancer pathogenesis. Although LSD2 histone demethylase has already been reported as an E3 ubiquitin ligase in lung cancer cells, we identified XIAP as the main E3 ubiquitin ligase in colon cancer cells. Interestingly, OGT catalyzes the O-GlcNAc modification of XIAP at serine 406 and this modification is required for the E3 ubiquitin ligase activity of XIAP toward specifically OGT. Moreover, O-GlcNAcylation of XIAP suppresses colon cancer cell growth and invasion by promoting the proteasomal degradation of OGT. Therefore, our findings regarding the reciprocal regulation of OGT and XIAP provide a novel molecular mechanism for controlling cancer growth and invasion regulated by OGT and O-GlcNAc modification.

## Introduction

The X-linked inhibitor of apoptosis protein (XIAP), also known as BIRC4, is a member of the IAP family and a potent inhibitor of the caspase-mediated apoptosis pathway^1^. XIAP possesses three N-terminal baculovirus IAP repeat domains, which mediate direct binding to caspases 3, 7, and 9 along with flanking residues. These residues are part of a C-terminal really interesting new gene (RING) domain that mediates the E3 ubiquitin ligase activity of XIAP and its involvement in the ubiquitin-proteasome pathway^2,3^. The ubiquitin-associated (UBA) domain and dimerization through the RING domain are important parts of XIAP binding to K63- and, in some cases, K48-linked polyubiquitin chains^4^. XIAP ubiquitinates a broad range of cellular substrates and is thereby involved in many cellular activities beyond its anti-apoptotic functions^5^. XIAP plays a role in the cancer cells’ resistance to anticancer drugs and studies on the anti-apoptotic function of XIAP in cancer cells have been focused on depleting XIAP^6^. XIAP has many other roles besides inhibiting cancer cell death, so its involvement in cancer cell biology should be explored further^7–13^.

The post-translational modification of proteins with O-linked N-acetylglucosamine (O-GlcNAc) occurs on the hydroxyl groups of serine or threonine residues, similar to phosphorylation^14,15^. O-GlcNAc modification is catalyzed by O-GlcNAc transferase (OGT) in the nucleus and cytoplasm^16^. The OGT-mediated transfer of beta N-acetylglucosamine from UDP-GlcNAc to target proteins plays a role in various biological processes, including protein–protein interactions, epigenetic regulation, protein stability and localization, and enzyme activity^17–21^. The aberrant O-GlcNAc modification of cellular proteins has been found in various types of cancers, including bladder, colorectal, lung, and pancreatic cancers^22–26^. OGT overexpression and increased O-GlcNAc modification are correlated with the histological grade of breast cancer tumors^27,28^, suggesting that OGT plays a critical role in tumorigenesis. Although OGT protein expression is high in cancer cells, little is known about its regulatory mechanisms.

It has been recently reported that LSD2 histone demethylase acts as an E3 ubiquitin ligase and promotes the proteasomal degradation of OGT in A549 lung cancer cells^29^. However, the mechanisms that regulate OGT expression in other types of cancer cells are still not well understood. This study identified XIAP as another E3 ubiquitin ligase of OGT in HCT116 human colorectal carcinoma cells. XIAP promotes the ubiquitin-dependent proteasomal degradation of OGT. Interestingly, XIAP is modified by O-GlcNAc on serine 406 which affects the E3 ubiquitin ligase activity of XIAP toward OGT but not toward other substrates, such as TGF-β1-activated kinase 1 (TAK1), caspase-3, and Cdc42^10,30–32^. HCT116 human colorectal carcinoma cells that consistently overexpress XIAP showed decreased cell proliferation and invasion. Thus, our findings reveal a new molecular mechanism regulating OGT and XIAP as key factors in cancer growth and invasion.

## Results

### OGT interaction with XIAP

**Fig. 1:**
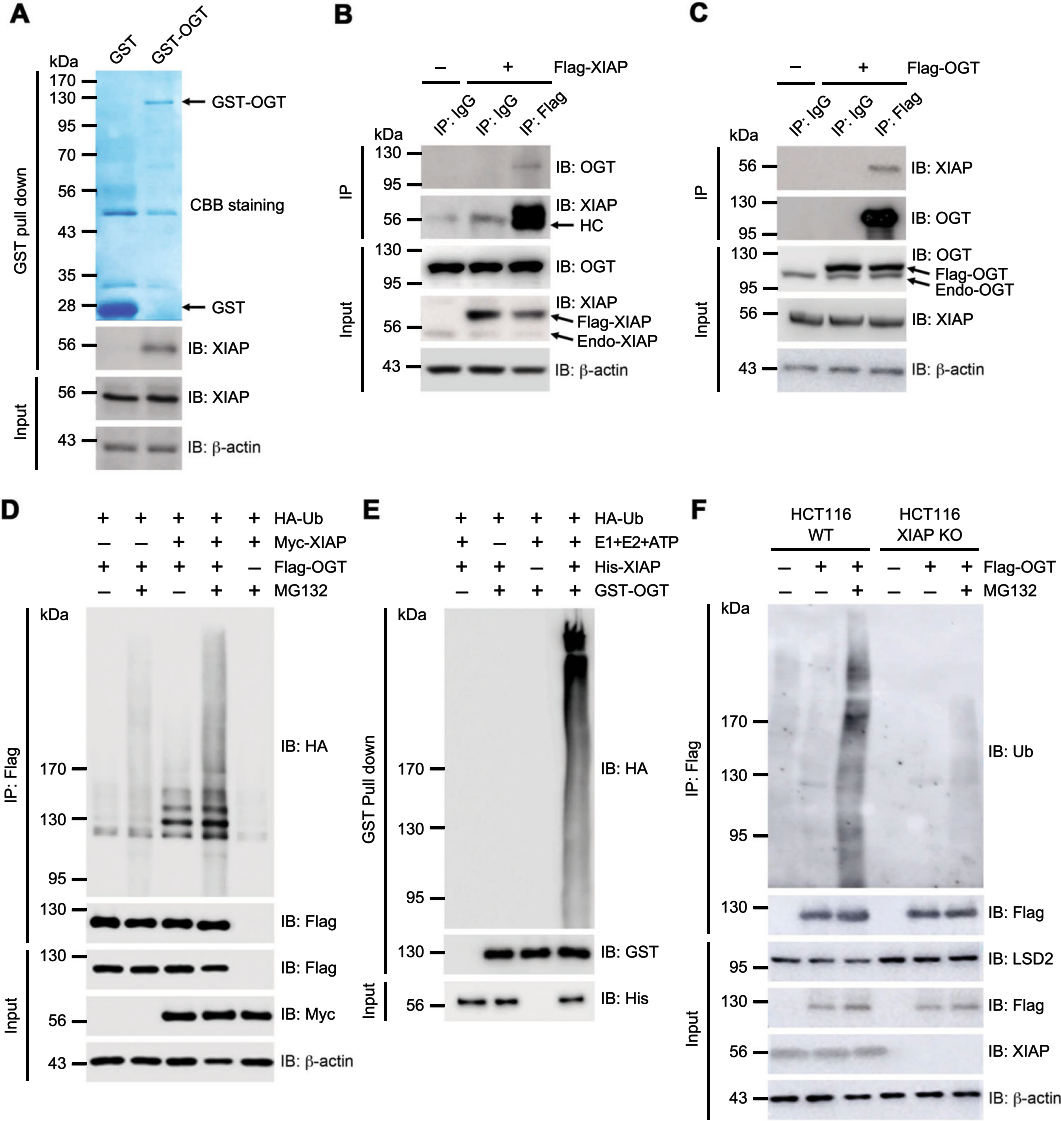
XIAP acts as an E3 ubiquitin ligase for OGT. **a** HCT16 cell lysates were incubated with immobilized recombinant GST-OGT fusion protein (residues 1–1036). GST-OGT was precipitated and the associated XIAP was detected by western blotting with α-XIAP antibodies. GST and GST-OGT were detected by Coomassie brilliant blue staining to verify the amount of protein used in the assay. **b** HCT116 cells transiently overexpressing Flag-tagged XIAP were immunoprecipitated with α-Flag or IgG control antibodies. Co-immunoprecipitated endogenous OGT was detected by α-OGT antibodies. The same membrane was re-probed with α-XIAP antibodies. Total lysates were blotted with α-OGT and α-XIAP antibodies. **c** HCT116 cells were transfected with Flag-tagged OGT and immunoprecipitated with α-Flag or IgG control antibodies. Bound endogenous XIAP was detected by α-XIAP antibodies. The same membrane was re-probed with α-OGT antibodies. Total lysates were blotted with α-OGT and α-XIAP antibodies. **d** OGT in vivo ubiquitination assay. Expression vectors encoding Flag-OGT and HA-ubiquitin (Ub) were transfected into HCT116 cells transiently overexpressing Myc-XIAP as indicated. The cells were treated with 20 μM of MG132 for 4 hours (h) before they were harvested. After being immunoprecipitated with α-Flag antibodies, the ubiquitination of OGT was analyzed by immunoblotting with α-HA antibodies. The same membrane was re-probed with α-Flag antibodies. Equal amounts of total lysates were subjected to immunoblotting with the indicated antibodies. **e** OGT in vitro ubiquitination assay. GST-OGT was incubated with His-XIAP, HA-Ub, E1, E2, and ATP as indicated. After GST pull-down under denaturing conditions with a buffer containing 2% SDS, the ubiquitination of OGT was analyzed by immunoblotting with α-HA antibodies. The same membrane was re-probed with α-GST antibodies. Equal amounts of XIAP in the reaction were immunoblotted with α-His antibodies. **f** HCT116 WT or HCT116 XIAP KO cells were transfected with Flag-OGT and treated with 20 μM of MG132 for 4 h. The ubiquitination of immunoprecipitated OGT was analyzed by immunoblotting with α-Ub antibodies. The same membrane was re-probed with α-Flag antibodies. Equal amounts of total lysates were subjected to immunoblotting as indicated. β-actin was used as a loading control. All data are representative of at least three independent experiments.

High levels of OGT protein and O-GlcNAcylation have a strong effect in determining the characteristics of many types of cancer cells^33^. We examined the proteins that regulate OGT in HCT116 cells because previous research has shown that silencing OGT significantly reduces cell proliferation and migration in human colon cancer cells^34^. In this study, glutathione S-transferase (GST) conjugated OGT were used for a pull-down assay and a subsequent mass spectrometry (MS) analysis to identify the proteins that interact with OGT in HCT116 cells (Supplementary Table S1). Among the proteins that interact with OGT, XIAP was studied because it plays an important role in tumorigenicity^7–13^. The pulled-down GST-OGT was immunoblotted with anti-XIAP antibody to confirm the interaction between GST-OGT and endogenous XIAP. The GST-OGT fusion protein interacted with endogenous XIAP (Fig. 1a). HCT116 cells transiently overexpressing XIAP or OGT were subjected to immunoprecipitation experiments to validate the interaction. Overexpressed Flag-tagged XIAP or Flag-tagged OGT interacted with endogenous OGT and XIAP, respectively (Fig. 1b, c). These results suggest that OGT interacts with XIAP in HCT116 cells.

### OGT is a substrate of the E3 ubiquitin-protein ligase XIAP

Ubiquitination as one of the most versatile post-translational modifications is mediated by a three-step process caused by ubiquitin-activating enzymes (E1), ubiquitin-conjugating enzymes (E2) and ubiquitin ligases (E3)^35^. Thus, the finding that XIAP interacts with OGT prompted the investigation of whether XIAP ubiquitinates OGT in vivo because XIAP acts as an E3 ubiquitin ligase^5,10,30–32^. Expression vectors encoding Flag-tagged OGT and HA-tagged ubiquitin (Ub) were co-transfected into HCT116 cells transiently expressing Myc-XIAP or empty vectors. Then OGT ubiquitination levels were measured in the presence of MG132, a proteasome inhibitor. OGT ubiquitination levels were found to be higher in HCT116 cells with Myc-XIAP than in HCT116 cells without Myc-XIAP (Fig. 1d). Pull-down experiments were performed by incubating the GST-OGT fusion protein with His-tagged XIAP, E1 (UBE1), and E2 (UbcH5c) under stringent denaturing conditions with a buffer containing 2% SDS to determine whether XIAP could ubiquitinate OGT in vitro. GST-OGT was polyubiquitinated in the presence, but not in the absence, of His-XIAP (Fig. 1e).

In vivo ubiquitination assays showed much lower levels of OGT ubiquitination in XIAP-deficient HCT116 (XIAP KO) cells than in wild-type HCT116 (WT) cells (Fig. 1f). Weak polyubiquitination was still detected in XIAP KO cells. Previous studies have reported that histone demethylase LSD2 acts as an E3 ubiquitin ligase for OGT^29^, so LSD2 protein levels in XIAP KO cells were compared to those in WT cells. Increased LSD2 levels were detected in XIAP KO cells (Fig. 1f). Thus, the weak polyubiquitinated OGT in XIAP KO cells may be attributed to the activity of LSD2. These results demonstrate that XIAP acts as an E3 ubiquitin ligase and ubiquitinates OGT in HCT116 colon cancer cells.

### OGT is degraded by XIAP-mediated ubiquitination

**Fig. 2:**
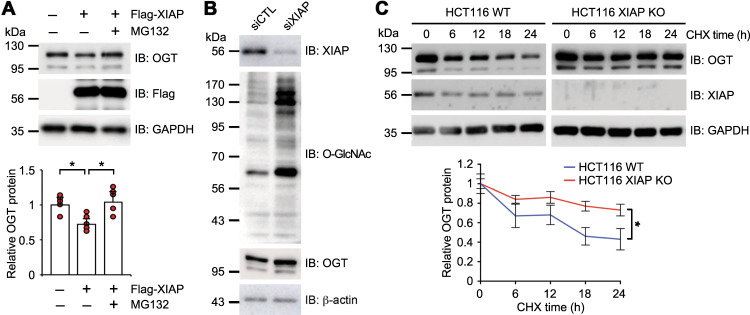
XIAP promotes OGT protein degradation. **a** Empty or expression vectors encoding Flag-XIAP were transfected into HCT116 cells as indicated. The cells were either treated with 20 μM of MG132 or not treated with any MG132 as indicated before being harvested. The expression levels of OGT, XIAP, and GAPDH were then determined and the relative OGT protein levels were plotted (*n* = 5 per condition). **b** HCT116 cells were transfected with XIAP siRNAs. Total lysates were harvested 48 h after transfection. O-GlcNAc and OGT levels were monitored. **c** HCT116 WT or HCT116 XIAP KO cells were treated with 10 μM of cycloheximide (CHX) and then harvested at the indicated times. The expression levels of OGT, XIAP, and GAPDH were determined by immunoblotting and the relative OGT protein levels were plotted (*n* = 5 per condition). β-actin or GAPDH was used as a loading control. Data are presented as means ± SD of at least three independent experiments. Statistical significance was determined using one-way analysis of variance (**a**) or Student’s *t* test (**c**). *P < 0.05, **P < 0.01.

Protein ubiquitination regulates protein degradation and subcellular signaling^35^. HCT116 cells were transfected with Flag-XIAP and the protein levels of endogenous OGT were measured in the presence or absence of MG132 to investigate the function of XIAP-mediated ubiquitination of OGT. The level of endogenous OGT decreased after Flag-XIAP transfection, whereas the XIAP-mediated degradation of OGT was blocked by the addition of MG132 (Fig. 2a). No significant differences in OGT mRNA levels were observed between Flag-XIAP-transfected cells regardless of whether they were treated with MG132 (Supplementary Fig. S1a). The down-regulation of XIAP by using siRNA led to increased expression of endogenous OGT in HCT116 cells (Fig. 2b). The half-life of OGT was measured in HCT116 WT and XIAP KO cells in the presence of cycloheximide (CHX), a protein synthesis inhibitor to further explore this relationship. OGT protein levels decreased at a slower rate in XIAP KO cells than in WT cells (Fig. 2c), whereas OGT mRNA levels in the two cell lines were not significantly different (Supplementary Fig. S1b). These results suggest that the turnover of OGT is regulated at the protein level, possibly through a ubiquitin-dependent pathway. Collectively, these results indicate that XIAP promotes the proteasomal-dependent degradation of OGT in HCT116 colon cancer cells.

### XIAP is modified by O-GlcNAc

**Fig. 3:**
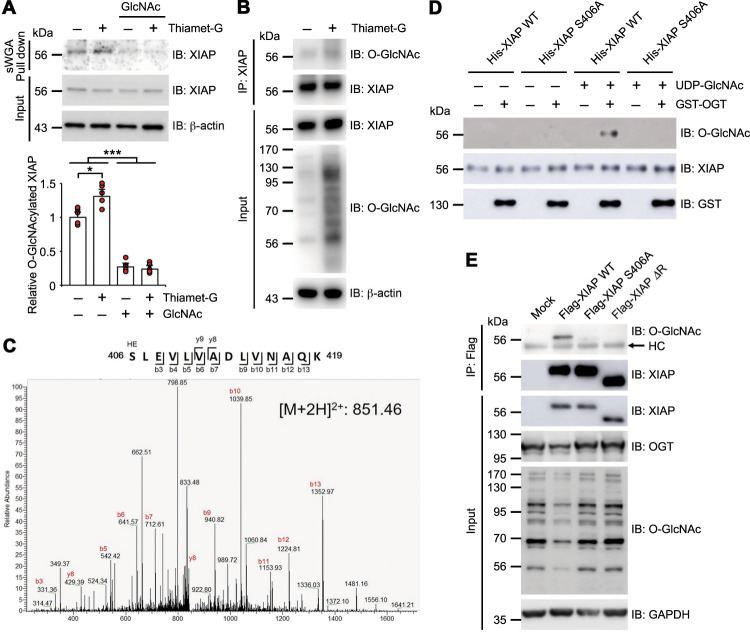
XIAP is modified by O-GlcNAc at Serine 406. **a** HCT116 cells were either treated with 1 µM of Thiamet-G for 4 h or not treated with any Thiamet-G. Cell lysates were then subjected to sWGA lectin affinity purification and analyzed with Western blotting for the presence of endogenous XIAP. As a control, 20 mM of the monosaccharide inhibitor GlcNAc was added during sWGA lectin affinity purification. Relative O-GlcNAcylated XIAP levels were plotted (*n* = 5 per condition). **b** HCT116 cells were either treated with 1 µM of Thiamet-G for 4 h or not treated with any Thiamet-G. Immunoprecipitated endogenous XIAP was detected by α-O-GlcNAc antibodies. **c** Expression vectors encoding Flag-OGT were transiently transfected into HEK293 cells and immunoprecipitated Flag-XIAP was subjected to MS analysis. The CTD MS/MS spectrum of residues 406–419, O-GlcNAcylated XIAP peptides, with the doubly charged precursor ion *m/z* 851.461426 (M + 2H)^2+^ is shown. The b- and y-type product ions were assigned. **d** Identification of the O-GlcNAc modification sites of XIAP by in vitro glycosylation assay. Purified WT or mutant His-tagged XIAP were used as substrates. O-GlcNAc-modified XIAP was analyzed by α-O-GlcNAc antibodies. The same membrane was re-probed with α-XIAP antibodies. Immunoblotting with α-GST antibodies was conducted to ensure that there was an equal amount of OGT. **e** Flag-XIAP WT, XIAP S406A, or XIAP ΔRING mutants were transiently overexpressed in HCT116 XIAP KO cells. XIAP WT and XIAP mutants were immunoprecipitated with α-Flag antibodies and blotted with α-O-GlcNAc and α-XIAP antibodies. Equal amounts of total lysates were subjected to immunoblotting with antibodies as indicated. β-actin or GAPDH was used as a loading control. Data are presented as means ± SD of at least three independent experiments. Statistical significance was determined using one-way analysis of variance. *P < 0.05, ***P < 0.001.

XIAP interaction with OGT suggested that XIAP may be modified by O-GlcNAc. Endogenous XIAP was examined to determine whether XIAP is a substrate of OGT. The Western blotting with XIAP antibodies of HCT116 total cell lysates that were affinity purified with succinylated wheat germ agglutinin (sWGA)^36^ showed that endogenous XIAP is O-GlcNAc-modified (Fig. 3a). The treatment of Thiamet-G, a selective inhibitor of O-GlcNAcase, resulted in a significant increase of O-GlcNAc modification of XIAP. The specificity of the lectin affinity purification was demonstrated by addition of the inhibitory monosaccharide GlcNAc (Fig. 3a). In fact, endogenous XIAP was detected with O-GlcNAc antibodies and the treatment of Thiamet-G induced a significant increase in XIAP O-GlcNAcylation (Fig. 3b). Mass spectrometry (MS) analysis was then employed to identify XIAP O-GlcNAc sites. HEK293 cell lysates transfected with Flag-XIAP were prepared instead of HCT116 cells to acquire the necessary amount of Flag-XIAP for MS analysis and to detect whether XIAP-induced polyubiquitination of OGT occurred in HEK293 cells as in HCT116 cells (Supplementary Fig. S2a). Thereafter, XIAP was separated by immunoprecipitation and SDS-PAGE and MS analysis was performed through collision-induced dissociation (CID) fragmentation method to analyze the protein. The CID MS/MS spectrum of the 2+ ion at *m/z* 851.46 corresponding to O-GlcNAcylated XIAP peptide SLEVLVADLVNAQK identified amino acids 406–419, the O-GlcNAcylated parent peptides of XIAP (Fig. 3c and Supplementary Fig. S2b). However, the exact O-GlcNAcylation site in the O-GlcNAcylated peptide could not be identified due to the limitation of CID. Despite these limitations, only one serine residue was identified in the parent peptide, suggesting that serine 406 was modified by O-GlcNAc.

Generation of a site-specific point mutant of XIAP showed that mutation of serine 406 to alanine (S406A) abolished the O-GlcNAc modification in in vitro O-GlcNAcylation assays (Fig. 3d). Flag-XIAP constructs were transiently expressed in HCT116 XIAP KO cells to further confirm the result of the in vitro O-GlcNAcylation assay. Substitution of serine 406 with alanine resulted in a significant reduction of O-GlcNAc modification of XIAP, indicating that XIAP was O-GlcNAcylated on serine 406 (Fig. 3e). Moreover, transiently overexpressing XIAP WT induced the reduction of endogenous OGT proteins and total cellular O-GlcNAc modification levels more than transiently overexpressing XIAP S406A or XIAP lacking the C-terminal RING domain (Fig. 3e), which has E3 ubiquitin ligase activity^2,3^. These results revealed that O-GlcNAc modification of XIAP at serine 406 affects the degradation of OGT in a RING domain-dependent manner. Co-immunoprecipitation was performed between XIAP and OGT to investigate the effect that substituting serine 406 with alanine on protein–protein interaction. Both XIAP S406A and XIAP lacking the RING domain interacted with OGT as much as XIAP WT, indicating that the interaction between XIAP and OGT was still functional (Supplementary Fig. S3). These results show that XIAP is modified by O-GlcNAc at serine 406 and that this modification directly influences the degradation of OGT.

### O-GlcNAc modification of XIAP at serine 406 is essential for its E3 ubiquitin ligase activity toward OGT

**Fig. 4:**
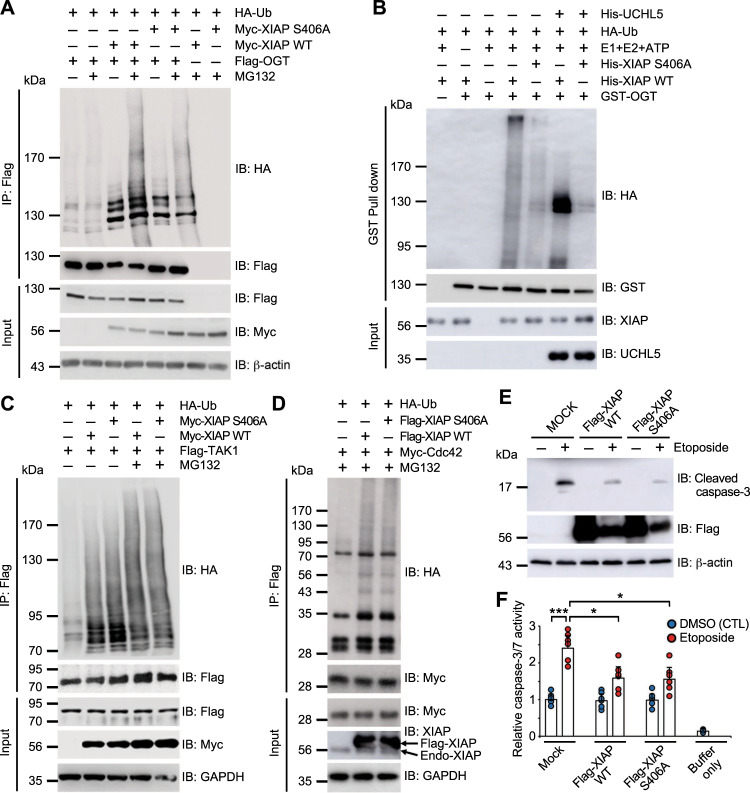
Mutation of XIAP reduced its E3 ubiquitin ligase activity for OGT. **a** Expression vectors encoding Flag-OGT and HA-Ub were transfected into HCT116 cells transiently overexpressing Myc-XIAP WT and Myc-XIAP S406A as indicated. Cells were treated with 10 μM of MG132 for 4 h before being harvested. After immunoprecipitation with α-Flag antibodies, the ubiquitination of OGT was analyzed by immunoblotting with α-HA antibodies. The same membrane was re-probed with α-Flag antibody. Equal amounts of total lysates were subjected to immunoblotting with the indicated antibodies. **b** GST-OGT was incubated with HA-Ub, E1, E2, ATP, His-UCHL5, and either His-XIAP WT or His-XIAP S406A in the presence of UDP-GlcNAc as indicated. After GST pull-down was conducted under denaturing conditions with a buffer containing 2% SDS, the ubiquitination of OGT was detected by immunoblotting with α-HA antibodies. The same membrane was re-probed with α-GST antibodies. The amounts of XIAP and UCHL5 in the reaction were detected with α-XIAP antibody and α-His antibodies, respectively. **c** Expression vectors encoding Flag-TAK1 and HA-Ub were transiently transfected into HCT116 cells overexpressing Myc-XIAP as indicated. Cells were treated with 10 μM of MG132 for 4 h before being harvested. After immunoprecipitation with α-Flag antibodies, the ubiquitination of TAK1 was analyzed by immunoblotting with α-HA antibodies. The same membrane was re-probed with α-Flag antibodies. Equal amounts of total lysates were subjected to immunoblotting with the indicated antibodies. **d** Expression vectors encoding Myc-Cdc42 and HA-Ub were transiently transfected into HCT116 cells overexpressing Flag-XIAP as indicated. Before harvest, cells were treated with 20 μM of MG132 for 4 h. After immunoprecipitation with α-Myc antibodies, the ubiquitination of OGT was analyzed by immunoblotting with α-HA antibodies. The same membrane was re-probed with α-Myc antibodies. Equal amounts of total lysates were subjected to immunoblotting with the indicated antibodies. **e** Expression vectors encoding Flag-XIAP WT and XIAP S406A were transfected into HCT116 cells. Cells were treated with 20 µM of etoposide for 24 h and lysed with NET buffer before being harvested. Activated caspase 3 was monitored with α-cleaved caspase 3 antibodies. Equal amounts of total lysates were subjected to immunoblotting with the indicated antibodies. **f** HCT116 cells were transfected with Myc-XIAP WT or Myc-XIAP S406A. Cells were treated with 20 µM of etoposide for 24 h and lysed with NET buffer before being harvested. Aliquots containing 50 µg of lysate were added to 100 µM of Ac-DEVD-AFC for measuring caspase-3/7 activity (*n* = 6 per condition). Initial rates were analyzed at Ex/Em = 380 / 500 nm. β-actin or GAPDH was used as a loading control. Data are presented as means ± SD of at least three independent experiments. Statistical significance was determined using one-way analysis of variance. *P < 0.05, ***P < 0.001.

The serine 406 residue of XIAP is located in the UBA domain^4^. XIAP’s UBA domain is an essential part of its ability to bind to K63- and, in some cases, K48-linked polyubiquitin^4^, so it was hypothesized that the O-GlcNAc modification of XIAP affects its ability to ubiquitinate OGT. The effect of the substitution of serine 406 with alanine on the E3 ubiquitin ligase activity of XIAP toward OGT was investigated using an in vivo ubiquitination assay. Flag-OGT, HA-Ub, and either Myc-XIAP WT or Myc-XIAP S406A were overexpressed in HCT116 cells. In the presence of MG132, the level of OGT ubiquitination was lower in XIAP S406A transfected cells than in XIAP WT transfected cells (Fig. 4a). This result was supported by an in vitro ubiquitination assay. His-XIAP WT ubiquitinated GST-OGT more efficiently than His-XIAP S406A (Fig. 4b). His-tagged ubiquitin C-terminal hydrolase L5 (UCHL5) was added to an in vitro ubiquitination assay to investigate whether the detected polyubiquitination of OGT was K48-polyubiquitination (Fig. 4b). The significant reduction of polyubiquitinated OGT indicated that OGT is modified by K48-polyubiquitination.

Next the effect of the substitution of serine 406 with alanine of XIAP on its E3 ubiquitin ligase activity toward OGT was examined because TAK1, Cdc42, and caspase-3 are XIAP substrates^10,30–32^. Plasmid vectors encoding Flag-TAK1, HA-ubiquitin, and either Myc-XIAP WT or Myc-XIAP S406A were transfected in HCT116 cells. The cells were then incubated with the proteasome inhibitor MG132 to prevent TAK1 degradation. The levels of the high molecular weight shift of TAK1 were increased, similar to their levels in both transiently overexpressing Myc-XIAP WT and Myc-XIAP S406A cells (Fig. 4c). In addition, both XIAP WT and XIAP S406A efficiently polyubiquitinated Cdc42 when Myc-Cdc42 and HA-ubiquitin were transfected in HCT116 cells with Flag-XIAP (Fig. 4d). Next, the inhibitory effects of XIAP WT and XIAP S406A on endogenous caspase-3 activation were assessed during topoisomerase II inhibitor etoposide-induced apoptosis in HCT116 cells. There was no significant difference in the activation of caspase-3 between transiently overexpressing XIAP WT and XIAP S406A cells (Fig. 4e, f). In addition, the XIAP S406A mutant showed the same levels of auto-ubiquitination as XIAP WT (Supplementary Fig. S4). These results indicate that O-GlcNAc modification on XIAP at serine 406 is required for XIAP’s E3 ubiquitin ligase activity specifically toward OGT.

### XIAP inhibits HCT116 cell growth by mediating OGT degradation

**Fig. 5:**
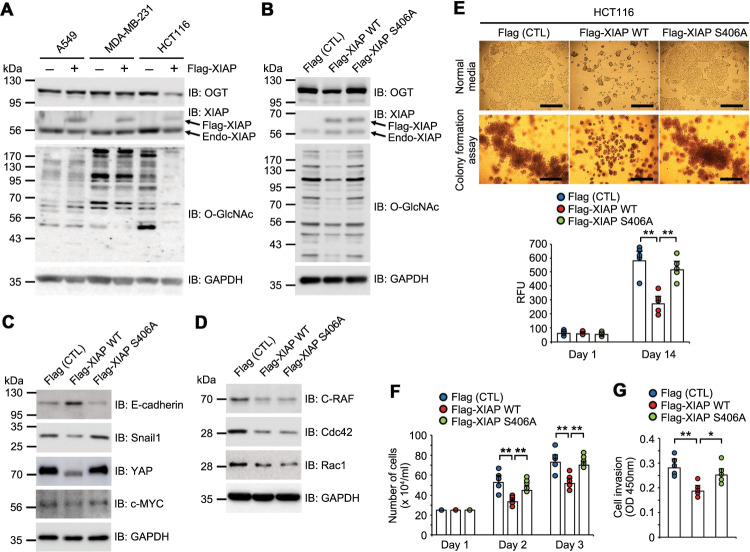
XIAP inhibits HCT116 colorectal carcinoma cell growth by inducing the degradation of OGT. **a** A549, MDA-MB231, and HCT116 overexpressing Flag-XIAP WT cell lines were established by retroviral particle packaging. Total cell lysates were immunoblotted with the indicated antibodies. **b**–**d** Establishing HCT116 cell lines overexpressing Flag-XIAP WT and Flag-XIAP S406A were established by retroviral particle packaging. Total cell lysates were immunoblotted with the indicated antibodies. **e** HCT116 cells overexpressing Flag-XIAP WT and Flag-XIAP S406A were cultured under normal conditions (top) or for soft agar colony formation assay (bottom) as described in the methods. Cell colony formation was examined under a light microscope. Colony formation was measured by using a 485/520 nm filter set (*n* = 5 per condition). Scale bars, 500 μm. **f** HCT116 cells overexpressing Flag-XIAP WT and Flag-XIAP S406A were treated with trypsin and counted for the duration of the indicated time by using 0.4% Trypan blue staining and a Neubauer-improved counting chamber. The values indicate the number of cells per ml (*n* = 5 per condition). **g** HCT116 cells stably overexpressing Flag-XIAP WT and Flag-XIAP S406A were seeded in trans-well chambers containing membranes with a pore size of 8 μm coated in Matrigel matrix. After 2 and 4 days, the number of cells that had been invaded were measured by using the kit-8 cell counting kit (*n* = 5 per condition). GAPDH was used as a loading control. Data are presented as means ± SD of at least three independent experiments. Statistical significance was determined using one-way analysis of variance. *P < 0.05, **P < 0.01.

Previous studies have revealed the roles that XIAP plays in cell migration and motility through its E3 ubiquitin ligase activity^7–13^. OGT is overexpressed in various cancer cells and plays an important role in their growth^23^. Human lung carcinoma A549 cells, human breast cancer MDA-MB-231 cells, and colorectal carcinoma HCT116 cells all stably expressing Flag-XIAP WT were established and their endogenous OGT protein and total cellular O-GlcNAc modification levels were investigated to explore the role of XIAP and OGT in cancer cells (Fig. 5a). The degradation of endogenous OGT was found to be more susceptible to XIAP WT in HCT116 cells than in lung and breast cancer cells. Therefore, HCT116 cells were employed as cells stably overexpressing XIAP WT or mutant XIAP S406A. OGT and total O-GlcNAcylation levels were lower in HCT116 cells overexpressing Flag-XIAP WT than in those overexpressing Flag (CTL) and Flag-XIAP S406A (Fig. 5b).

Decreased OGT protein and total O-GlcNAcylation levels in stable Flag-XIAP WT-expressing cells were associated with increased levels of E-cadherin, a key regulator of epithelial-mesenchymal transition, and decreased levels of Snail1, a transcriptional repressor of E-cadherin (Fig. 5c). Snail1 is modified by O-GlcNAc which affects its stability^37^. Yes-associated protein (YAP) and c-MYC were selected to further confirm the specific contribution of O-GlcNAc-modified XIAP-dependent OGT degradation in cancer cells because both proteins are potent oncogenic factors and regulated by O-GlcNAc modification^38–40^. The levels of YAP and c-MYC were lower in XIAP WT-overexpressing cells than in CTL and XIAP S406A cells (Fig. 5c). The levels of XIAP-regulating tumorigenic proteins, including C-RAF, Cdc42, and Rac1 were also examined^7–13^. C-RAF, Cdc42, and Rac1 levels found to be reduced in both stably XIAP-overexpressing cells (Fig. 5d). XIAP S406A-overexpressing cells had similarly reduced levels of C-RAF, Cdc42, and Rac1 as XIAP WT (Fig. 5d).

**Fig. 6:**
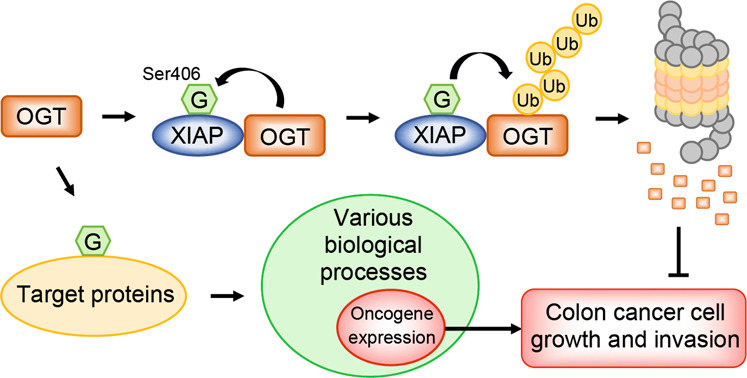
A model for the mutual regulatory function of OGT and XIAP. OGT mediates the O-GlcNAc modification of various target proteins and regulates various cellular processes, including oncogene expression. XIAP interacts with OGT and act as an E3 ubiquitin ligase for OGT. Expectedly, XIAP is also a substrate of OGT and O-GlcNAcylation of XIAP at serine 406 induces the polyubiquitination and degradation of OGT, leading to the inhibition of colon cancer cell growth and invasion.

Silencing OGT has been shown to reduce proliferation^34^ and XIAP depletion promotes the migration and motility of cancer cells^7–13^. In normal culture conditions, the stable overexpression of XIAP WT decreased the proliferation of colon cancer cells, while XIAP S406A cells had similar proliferation rates as CTL cells (Fig. 5e). Moreover, soft agar colony formation assays to observe cluster growth showed that XIAP WT-overexpressing cells formed smaller colonies than CTL cells and XIAP S406A cells (Fig. 5e). A cell counting assay was also performed to compare the growth of cells overexpressing XIAP WT compared with that of cells overexpressing CTL and XIAP S406A. The stable introduction of XIAP WT genes by viral infection decreased HCT116 cell growth more than the introduction of CTL and XIAP S406A genes (Fig. 5f). Next, the effect of overexpressed XIAP on cancer cell invasion was examined because XIAP-induced OGT degradation decreased Snail1 levels which in turn upregulated E-cadherin expression^37^. The invasion was lower in HCT116 cells overexpressing XIAP WT than HCT116 cells overexpressing CTL and XIAP S406A (Fig. 5g). Taken together, these results show that XIAP is an E3 ubiquitin ligase of OGT, is modified by O-GlcNAc at serine 406, and this modification on XIAP leads to the polyubiquitination and degradation of OGT, resulting in the inhibition of colon cancer cell growth and invasion (Fig. 6).

## Discussion

Abnormally increased OGT and O-GlcNAcylation levels are found in most cancers and contribute to the transformation of cancer phenotypes^41–43^. However, it is not well known how OGT and O-GlcNAcylation levels are regulated in various cancers. This study demonstrated that XIAP acts as an E3 ubiquitin ligase for OGT, resulting in the proteasomal degradation of OGT and the inhibition of colon cancer cell growth and invasion. Additionally, XIAP is modified by O-GlcNAc at serine 406 which is important for the E3 ubiquitin ligase activity of XIAP for OGT.

XIAP has mostly been studied for its inhibition of caspases and its involvement in diverse subcellular processes, including copper metabolism, signal activation, and ubiquitination^5^. Apart from its function as a caspase inhibitor, XIAP in cancer cells inhibits cell adhesion, migration, and motility by controlling Rho GTPase proteins, such as Rac1 and Cdc42^8–13^. In addition to regulating Rho GTPase, XIAP modulates cell motility by direct ubiquitination of C-RAF^7^. These discoveries about XIAP’s unconventional roles in cancer were supported by this study which identified another molecular mechanism for the XIAP-mediated inhibition of cancer cell proliferation and invasion through the degradation of OGT.

XIAP is phosphorylated by several protein kinases, including protein kinase B (AKT), IkB kinase ε (IKKε), Tank-binding kinase 1 (TBK1), cyclin-dependent kinase 1 (CDK1)/cyclin-B1, and glycogen synthase kinase 3 (GSK3) at serine or threonine residues that affects the activity and stability of XIAP^44–47^. This study found that XIAP undergoes O-GlcNAc modification at serine 406. O-GlcNAcylation and phosphorylation could occupy the same serine or threonine residues, so this study also examined the possible phosphorylation sites on amino acids 406–419, XIAP’s O-GlcNAc modified peptides. The results showed no phosphorylation events on the O-GlcNAc-modified peptides (Supplementary Fig. S5). However, it has been reported that XIAP is phosphorylated at serine 406^48,49^. Therefore, the relationship between O-GlcNAc and phosphorylation as well as the function of phosphorylation on XIAP at serine 406 should be further investigated to regulate XIAP’s E3 ubiquitin ligase activity toward OGT.

The absence of O-GlcNAc modification on XIAP at serine 406 through the substitution of serine to alanine was found to decrease XIAP’s E3 ubiquitin ligase activity specifically toward OGT. In contrast, the mutation did not affect XIAP ubiquitin ligase activity toward other well-known substrates, such as TAK1, caspase-3, and Cdc42^10,30–32^ or XIAP auto-ubiquitination. The substrate specificity of XIAP affected by the mutation may be a result of the substrate specificity of the activity of E2 ubiquitin-conjugating enzymes during XIAP-mediated OGT ubiquitination. More than 30 active E2 enzymes have been identified in humans^50,51^. They are involved in multiple cellular functions with their own specific substrates^50,51^. This study showed that the E3 ubiquitin ligase activity of XIAP toward OGT could be modulated by UbcH5c, an E2 ubiquitin-conjugating enzyme, suggesting that XIAP’s O-GlcNAc modification on serine 406 might increase the binding affinity of XIAP with the specific E2 ubiquitin-conjugating enzyme UbcH5c. However, these findings also suggest that the ubiquitination of TAK1, caspase-3, and Cdc2 may be regulated by other E2 ubiquitin-conjugating enzymes, with the exception of UbcH5c, when XIAP functions as their E3 ubiquitin ligase.

This study showed that HCT116 colon cells stably overexpressing XIAP WT displayed greater changes in OGT and O-GlcNAc modification levels than A549 lung cancer cells and MDA-MB231 breast cancer cells. In addition, the transient overexpression of XIAP WT was found to decrease of OGT levels in SW13, SW480, and SW620 colon cancer cell lines (Supplementary Fig. S6). This variation in the effectiveness of XIAP to OGT regulation may be a product of the differences in the pool of O-GlcNAc-modified proteins in specific cancer types. These findings suggest that the levels of OGT and O-GlcNAc are regulated by different cell type-specific mechanisms despite the fact that most types of cancer cells have aberrantly high levels of OGT and O-GlcNAc modification. The expression of XIAP in breast cancer cells is regulated by FOXM1 which acts as an intermediate between XIAP and OGT^52,53^. In A549 lung cancer cells, LSD2 functions as an E3 ubiquitin ligase and promotes the proteasomal degradation of OGT^29^. Therefore, it could explain that stable XIAP overexpression would have a smaller effect on OGT and total O-GlcNAc levels in MDA-MB231 and A549 cells than in HCT116 colon cancer cells.

In summary, the OGT-mediated O-GlcNAc modification of XIAP at serine 406 is essential for its E3 ubiquitin ligase activity toward specifically OGT. Normal O-GlcNAc-modified XIAP mediates the ubiquitin-proteasomal degradation of OGT, resulting in the inhibition of colon cancer cell growth and invasion. Thus, a further research about the mutual regulatory mechanism between XIAP and OGT is needed to understand the pathogenesis of colon cancer and consider the potential therapeutic interest for colon cancer.

## Materials and methods

### Cell culture, DNA transfection, and plasmids

HEK293 cells and human colon cancer HCT116 WT and XIAP KO cells were cultured in Dulbecco’s Modified Eagle’s Medium (DMEM, Lonza, Basel, Switzerland) supplemented with 10% fetal bovine serum, 100 U/ml penicillin and 100 μg/ml streptomycin at 37 °C in 5% CO_2_. DNA was transfected using polyethylenimine (Sigma-Aldrich, St Louis, MO, USA) as described previously^54^ and FuGENE® HD (Promega, Madison, WI, USA) following the manufacturer’s protocol. Human OGT, WT XIAP and mutant S406A XIAP were cloned into the p3×FLAG-CMV™-7.1 expression vector (Sigma-Aldrich). Human XIAP WT, XIAP S406A, and XIAP ΔRING proteins were cloned into pRK5 in frame with an N-terminal Myc or Flag epitope. The mutant XIAP S406A was generated using the QuikChange Site-Directed Mutagenesis Kit (Stratagene, La Jolla, CA, USA). The mutations were confirmed by DNA sequence analysis. pRK5-Myc-Cdc42 WT is purchased from Addgene (Plasmid 12972, Cambridge, MA, USA). To generate HCT116 cells stably overexpressing XIAP WT and mutant XIAP S406A, His-tagged WT XIAP and mutant S406A XIAP were cloned into the pET6×HN expression vector (Clontech, Rockville, MD, USA).

### Reagents and antibodies

Antibodies against XIAP (E-2, HRP conjugated), c-Myc (9E10, mouse monoclonal), GST (B-14, mouse monoclonal), His (H8, mouse monoclonal) and GAPDH (6C5, mouse monoclonal) were purchased from Santa Cruz Biotechnology (Dallas, Texas, USA). Antibodies against Flag (F-3165, mouse monoclonal), HA (HA-7, mouse monoclonal) and OGT (DM17, rabbit polyclonal) were from Sigma-Aldrich (St. Louis. MO). Antibodies against XIAP (D2Z8W, rabbit monoclonal), Snail1 (C15D3, rabbit monoclonal), E-Cadherin (24E10, rabbit monoclonal), Ubiquitin (P4D1, mouse monoclonal), β-actin (13E5, rabbit monoclonal), C-RAF (D4B3J, rabbit monoclonal) and Cleaved caspase-3 (5A1E, rabbit monoclonal) were purchased from Cell Signaling Technology (Danvers, MA, USA). Antibodies against LSD2 (EPR18508, rabbit monoclonal), Cdc42 (EPR15620, rabbit monoclonal), Rac1 (23A8, mouse monoclonal) and YAP1 (EP1674Y, rabbit monoclonal) were purchased from Abcam (Cambridge, UK). CTD110.6, an antibody against O-GlcNAc, was purchased from BioLegend (San diego, CA, USA). E1 (UBE1, E1-305), E2 (UbcH5c, E2-627), HA-Ubiquitin (U-110) and HA-Ubiquitin Aldehyde (U-211) were purchased from Boston Biochem (Cambridge, MA, USA). Recombinant GST-TAK1 protein was purchased from Novus Biologicals (Littleton, CO, USA). Recombinant His-Ubiquitin protein and His-UCHL5 were purchased from R&D systems (Minneapolis, MN, USA) and LSBio (Seattle, WA, USA), respectively. Recombinant GST-XIAP was purchased from Creative BioMart (Shirley, NY, USA). Glutathione agarose and Ni-NTA agarose were purchased from Qiagen (Hilden, Germany). Protein A/G Agarose was purchased from ThermoFisher Scientific (Pittsburgh, PA, USA).

### sWGA affinity purification, immunoprecipitation, and western blot

HCT116 cells were lysed with NET lysis buffer (50 mM Tris-HCl, pH 7.4; 150 mM NaCl; 1 mM EDTA and 1% Nonidet P-40) supplemented with a protease inhibitor cocktail (Roche, Mannheim, Germany). Proteins of cell lysates concentrations were determined by the Bio-Rad protein assay (Hercules, CA, USA) and were incubated with agarose-conjugated succinylated wheat germ agglutinin (sWGA, Vector Laboratories, Burlingame, CA, USA) for 3 h at 4 °C. Protein samples were subjected to reducing SDS-PAGE and transferred to nitrocellulose membranes (Amersham, Piscataway, NJ, USA). For Western blot analysis, membranes were blocked with 5% low fat milk in Tris-Buffered Saline with 1% Tween-20 for 1 h and then incubated with various antibodies according to datasheets. Antigen antibody complexes were detected by incubating with horseradish peroxidase coupled secondary antibodies followed by SuperSignal West Dura Extended Duration Substrate (ThermoFisher Scientific). For immunoprecipitation, cell lysates were gently mixed with specific antibodies and protein A/G beads for 4 h at 4 °C. Immunoprecipitates were washed three times with lysis buffer, eluted with SDS sample buffer and subjected to reducing SDS-PAGE and western blotting. For endogenous immunoprecipitation, cells were lysed in co-immunoprecipitation buffer (50 mM Tris-HCl, pH 7.4; 150 mM NaCl; 0.5% Triton X-100; 1 mM DTT; 0.1 mM EDTA and a protease inhibitor cocktail) and incubated with antibodies together with A/G beads for overnight at 4 °C. Thereafter, the beads were washed three times with co-immunoprecipitation washing buffer (20 mM HEPES, pH 7.4; 2 mM MgCl_2_; 2 mM EGTA; 150 mM NaCl and 0.1% Triton X-100), suspended in sample buffer and subjected to SDS-PAGE and western blotting.

### In vivo and in vitro ubiquitination assays

For OGT in vivo ubiquitination assay, HCT116 cells were transiently co-transfected with Flag-tagged OGT, Myc-tagged XIAP and HA-tagged ubiquitin. After 48 h of transfection, cells were treated with MG132 (20 μM) for 4 h then harvested using RIPA buffer (50 mM Tris-HCl, pH 7.4; 150 mM NaCl; 1 mM EDTA; 0.5% deoxycholate; 0.1% SDS; 1 mM Na_3_VO_4_; 1 mM PMSF and 1% Triton X-100) supplemented with DTT (1 mM), ubiquitin aldehyde (5 μM) and protease inhibitor cocktail (Roche). The cell extracts were subjected to immunoprecipitation using anti-FLAG antibody-conjugated A/G beads or pull-down with Glutathione-Sepharose4B (GE Healthcare) beads under denaturing conditions with buffer containing 2% SDS. The beads were washed three times with RIPA buffer followed by SDS-PAGE and western blotting. For TAK1 and Cdc42 in vivo ubiquitination, the cell lysates were lysed with buffer containing 1% SDS and 1% NP-40, respectively.

For OGT in vitro ubiquitination, 2 μg GST-OGT and 300 ng His-XIAP (purified) were incubated with 150 ng E1 (UBE1), 400 ng E2 (UbcH5c), 5 μg HA-ubiquitin and 1 mM UDP-GlcNAc in OGT in vitro ubiquitination buffer containing 25 mM Tris-HCl (pH 8.0), 4 mM ATP, 5 mM MgCl_2_, 200 μM CaCl_2_ and 1 mM DTT. 500 ng UCHL5 was used for OGT in vitro ubiquitination assay. After incubation at 37 °C for 2 h, GST-OGT proteins were pulled down with GST beads. The beads were washed three times with the corresponding buffer, and the immobilized proteins were subjected to SDS-PAGE. The ubiquitination of OGT was analyzed by Western blotting with anti-HA antibody.

### Quantitative real-time PCR

Total RNA was isolated from HCT116 cells using the TRIzol reagent (Invitrogen, Carlsbad, CA, USA). Complementary DNA was synthesized using ReverTra Ace qPCR RT Master Mix (Toyobo Co., Osaka, Japan). Quantitative real-time PCR was performed using SYBR Premix Ex Taq (Takara, Otsu, Japan). Reactions were performed according to the manufacturer’s instructions. Amplified products were analyzed using the Applied Biosystems 7300 Real-Time PCR system. The mRNA levels of OGT were normalized to those of β-actin. Gene specific primers were as follows:

OGT, forward 5′-CTTTAGCACTCTGGCAATTAAACAG-3′,

OGT, reverse 5′-TCAAATAACATGCCTTGGCTTC-3′

β-actin, forward 5′- AGAGCTACGAGCTGCCTGAC-3′,

β-actin, reverse 5′- AGCACTGTGTTGGCGTACAG-3′.

### Transfection of siRNAs

To silence XIAP by RNA interference, HCT116 cells were seeded in a six well plate 24 h prior to transfection. siRNAs directed against XIAP genes and scrambled control siRNA as negative control were transfected using the Lipofectamine RNAi max kits (Invitrogen). At 48 h post transfection, cells were lysed with NET lysis buffer (150 mM NaCl, 50 mM Tris, pH 7.4, 1 mM EDTA, 1% Nonidet P-40) supplemented with a protease inhibitor cocktail (Roche, Mannheim, Germany). siRNAs have been transfected at 100 nM. The following siRNA were employed in this study as previously described^10^: XIAP siRNA, AAGTGCTTTCACTGTGGAGGA and Control siRNA, AATTCTCCGAACGTGTCACGT. siRNAs were synthesized (GenePharma, Shanghai, China).

### Mass spectrometry for the detection of GST-OGT interacting proteins

The peptide samples extracted by in-gel digestion were suspended in 20 µl of solvent A (0.1% formic acid prepared in water, Optima LC/MS grade, ThermoFisher Scientific). Thereafter, 2 µl of the sample was loaded onto an EASYSpray C18 column (75 µm × 50 cm, 2 µm) and separated with a 2–35% gradient of solvent B (0.1% formic acid prepared in ACN) for 65 min at a flow rate of 300 nL/min. Mass spectra were recorded on a QExactive hybrid quadrupole-Orbitrap mass spectrometer (Thermo Fisher Scientific) interfaced with a nano-ultraHPLC system (Easy-nLC1000; Thermo Scientific). Each cycle of survey consisted of full MS scan at the mass range 300–1400 *m/z* and MS/MS scan for ten most intense ions. Peptides were fragmented using Higher energy collision dissociation and the normalized collision energy value was set at 27%. Exclusion time of previously fragmented peptides was for 20 s. The raw data were processed by using the Trans-Proteomic Pipeline (v4.8.0 PHILAE) for converting to mzXML file which is search-available format. Database search for sequenced peptides was using the Sequest (version 27) algorithm in the SORCERER (Sage-N Research, Milpitas) platform with Uniprot human database. Database searching parameters were as follows: parent tolerance 10ppm(average), fragment tolerance 0.6 Da(average), Fixed modification on cysteine of 57 Da (carbamidomethylation), variable modification on methionine of 16 Da(oxidation).

### Mass spectrometry for mapping O-GlcNAc sites

The peptide samples extracted by in-gel digestion were suspended in 20 µl of solvent A (0.1% formic acid prepared in water, Optima LC/MS grade, ThermoFisher Scientific). Thereafter, 5 µl of the sample was loaded onto a house-packed 75 µm (inner diameter of microcapillary) × 15 cm C18 (5 µm, 200 Å) column and separated with a 2–30% gradient of solvent B (0.1% formic acid prepared in ACN) for 280 min at a flow rate of 300 nl/min. Mass spectra were recorded on an LTQ-Velos mass spectrometer (ThermoFisher Scientific) interfaced with an EASY-nLC (Proxeon Biosystems, Odense, Denmark). Data-dependent MS acquisition conditions were as follows: the spray voltage was set at 2.0 kV and the heated capillary was set at 325 °C. Each cycle of survey consisted of full MS scan at the mass range 300–1400 *m/z* and MS/MS scan for five most intense ions. Peptides were fragmented in the LTQ section for five most intense ions. Peptides were fragmented in the LTQ section using collision-induced dissociation and the normalized collision energy value was set at 35%. Exclusion time of previously fragmented peptides was for 30 s. The raw data were processed by using the Trans-Proteomic Pipeline (v4.8.0 PHILAE) for converting to mzML file which is search-available format. Database search for sequenced peptides was performed in the National Centre for Biotechnology Information (Bethesda, MD, USA) non‐redundant database using the MASCOT software package (Matrix Sciences, London, UK) with UniProt. Database searching parameters were as follows: parent tolerance 2.0 Da (average), fragment tolerance 1.00 Da (average), Fixed modification on cysteine of 57 Da (carbamidomethylation), variable modification on methionine of 16 Da (oxidation) and serine/threonine of 203 Da (HexNAc).

### Stable cell line establishment

XIAP was cloned into the retroviral vector pMSCV-Flag for stable cell establishment. Together with the retroviral packaging plasmids pVSV-G and Gag/Pol, 3 μg retroviral DNA constructs were transfected into 293T cells. Retroviral particles were collected twice at 24 and 48 h after transfection to infect target cells supplemented with 10 μg/ml final concentration of polybrene. At 24 h after infection, antibiotic selection was performed to obtain stable transgenic cells.

### Cell counting

HCT116 cells were seeded in 60-mm dishes at a density of 10,000 cells per plate. After the indicated treatments, the cells were incubated with 0.25% trypsin-EDTA (Gibco, Grand Island, NY, USA) for 1 min and then harvested. The cells were resuspended in 25 mM DMEM and diluted to a 1:1 ratio by mixing with 0.4% Trypan blue stain (Gibco) and incubating for 5 min. The stained cells were then transferred to a Neubauer improved chamber (Marienfeld, Lauda-Konigshofen, Germany), and viable cells were counted under an OLYMPUS CK2-TRC microscope. The number of cells in 0.1 μl of cell suspension was calculated by counting cells in four large squares, then dividing the sum of the four squares by 4 and multiplying by a dilution factor of 2. The number of cells per ml was obtained by multiplying the cell number from the previous calculation by 10,000.

### Soft agar colony formation assay

To assess colony formation, the CytoSelect 96-Well Cell Transformation Assay (Cell Biolabs, San Diego, CA, USA) was used according to the manufacturer’s instructions. Briefly, HCT116 cells were seeded in soft agar at a density of 10,000 cells per well. After 1 and 14 days of incubation at 37 °C in 5% CO_2_, colony formation was quantified by solubilizing soft agar, lysing cells and incubating cell lysates with the CyQUANT GR Dye (Cell Biolabs), followed by analysis with the Synergy^TM^ HT plate reader and the Gen5 software (BioTek, Winooski, VT, USA).

### Transwell invasion assay

Invasion assays were performed using Corning^®^ Matrigel^®^ Matrix (Corning, NY, USA) and a 24 mm Transwell with a 0.8 µm insert following the manufacturer’s protocol. After 2 and 4 days of incubation, invasive cells were quantified by the cell counting kit-8 assay.

### In vitro O-GlcNAcylation assay

Recombinant GST-OGT protein (4 μg) was incubated with 6 μg of recombinant His-XIAP WT and His-XIAP S406A in 50 μl reactions (50 Mm Tris-HCL, pH 7.5; 12.5 mM MgCl_2_; 2 mM UDP-GlcNAc and 1 mM DTT) for 6 h at 37 °C. Then, the reactions were resolved with SDS-PAGE and subjected to western blotting analysis.

### Caspase-3/7 activity assay

HCT116 cells were seeded 10,000 cells per well in a six well microplate. Cultured cells were transfected with Flag-tagged XIAP WT and Flag-tagged XIAP S406A prior to etoposide treatment. After 24 h, dispense cell extract at 150 μl per well (96 well plate) was gently mixed with the caspase-3/7 substrate solution by shaking for 1 min at 150 rpm at 37 °C. Fluorescence signal was measured at Ex/Em = 380 nm/500 nm. The procedure was followed by manufacturer’s protocol (AnaSpec, Fremont, CA, USA).

### Statistical analysis

All data are presented as the means ± SD from three independent experiments. Student’s unpaired *t*-test was used to compare the means of two groups. One-way Analysis of Variance (ANOVA) with Turkey’s multiple comparison tests was used to compute statistical significance between multiple groups. *P* values < 0.05 were considered statistically significant. Statistical significance was denoted by *P < 0.05, **P < 0.01, or ***P < 0.001.

## Supplementary information

Table S1. GST-OGT-interacting proteins.

Supplementary Fig. S1 Effects of XIAP overexpression on OGT mRNA level.

Supplementary Fig. S2 OGT is a substrate of XIAP and mediates the O-GlcNAcylation of XIAP in HEK293 cells.

Supplementary Fig. S3 The substitution of Serine 406 to alanine and the deletion of the RING domains in XIAP does not affect its interactions with OGT.

Supplementary Fig. S4 The substitution of Serine 406 to alanine in XIAP does not affect the auto-ubiquitination.

Supplementary Fig. S5 MS analysis for phosphorylation residues on XIAP.

Supplementary Fig. S6 Effects of XIAP overexpression on OGT protein level in several colon cancer cell lines.

Supplementary Figure legends (Fig S1-S6)
