# Supplementary material for: Mutual regulation between OGT and XIAP to control colon cancer cell growth and invasion
Source: Cell Death Dis. 2020 Sep 29;11(9):815. doi: 10.1038/s41419-020-02999-5 (PMC7525441; doi:10.1038/s41419-020-02999-5)
Supplement: Supplementary file 1 — Table S1. GST-OGT-interacting proteins. [file 41419_2020_2999_MOESM1_ESM.docx]

**Supplementary Table S1. GST-OGT-interacting proteins.**

| # | Identified Proteins (716) | Accession Number | Molecular  weight |
| --- | --- | --- | --- |
| 1 | Heat shock 60kDa protein 1 (Chaperonin), isoform CRA_a OS=Homo sapiens GN=HSPD1 PE=2 SV=1 | tr\|A0A024R3X4\|A0A024R3X4_HUMAN | 61 kDa |
| 2 | Tubulin beta chain OS=Homo sapiens GN=TUBB PE=1 SV=2 | sp\|P07437\|TBB5_HUMAN | 50 kDa |
| 3 | Enolase 1, (Alpha), isoform CRA_a OS=Homo sapiens GN=ENO1 PE=2 SV=1 | tr\|A0A024R4F1\|A0A024R4F1_HUMAN (+1) | 47 kDa |
| 4 | Tubulin alpha-1B chain OS=Homo sapiens GN=TUBA1B PE=1 SV=1 | sp\|P68363\|TBA1B_HUMAN | 50 kDa |
| 5 | Heterogeneous nuclear ribonucleoprotein H OS=Homo sapiens GN=HNRNPH1 PE=1 SV=1 | tr\|G8JLB6\|G8JLB6_HUMAN | 51 kDa |
| 6 | Elongation factor 1-gamma OS=Homo sapiens GN=EEF1G PE=1 SV=3 | sp\|P26641\|EF1G_HUMAN (+1) | 50 kDa |
| 7 | T-complex protein 1 subunit beta OS=Homo sapiens GN=CCT2 PE=1 SV=4 | sp\|P78371\|TCPB_HUMAN (+1) | 57 kDa |
| 8 | ATP synthase subunit beta OS=Homo sapiens GN=HEL-S-271 PE=1 SV=1 | tr\|V9HW31\|V9HW31_HUMAN | 57 kDa |
| 9 | 60S ribosomal protein L4 OS=Homo sapiens GN=RPL4 PE=1 SV=5 | sp\|P36578\|RL4_HUMAN (+1) | 48 kDa |
| 10 | Protein disulfide-isomerase OS=Homo sapiens GN=HEL-S-269 PE=2 SV=1 | tr\|V9HVY3\|V9HVY3_HUMAN | 57 kDa |
| 11 | Polypyrimidine tract-binding protein 1 OS=Homo sapiens GN=PTBP1 PE=1 SV=1 | sp\|P26599\|PTBP1_HUMAN | 57 kDa |
| 12 | Heterogeneous nuclear ribonucleoprotein K OS=Homo sapiens GN=HNRNPK PE=1 SV=1 | sp\|P61978\|HNRPK_HUMAN | 51 kDa |
| 13 | Nuclease-sensitive element-binding protein 1 OS=Homo sapiens GN=YBX1 PE=1 SV=3 | sp\|P67809\|YBOX1_HUMAN | 36 kDa |
| 14 | tRNA-splicing ligase RtcB homolog OS=Homo sapiens GN=RTCB PE=1 SV=1 | sp\|Q9Y3I0\|RTCB_HUMAN | 55 kDa |
| 15 | Plasminogen activator inhibitor 1 RNA-binding protein OS=Homo sapiens GN=SERBP1 PE=1 SV=2 | sp\|Q8NC51\|PAIRB_HUMAN | 45 kDa |
| 16 | Actin, cytoplasmic 1 OS=Homo sapiens GN=ACTB PE=1 SV=1 | sp\|P60709\|ACTB_HUMAN (+1) | 42 kDa |
| 17 | Importin subunit alpha-1 OS=Homo sapiens GN=KPNA2 PE=1 SV=1 | sp\|P52292\|IMA1_HUMAN (+1) | 58 kDa |
| 18 | T-complex protein 1 subunit theta OS=Homo sapiens GN=CCT8 PE=1 SV=4 | sp\|P50990\|TCPQ_HUMAN | 60 kDa |
| 19 | Glutamate-rich WD repeat-containing protein 1 OS=Homo sapiens GN=GRWD1 PE=1 SV=1 | sp\|Q9BQ67\|GRWD1_HUMAN | 49 kDa |
| 20 | Lupus La protein OS=Homo sapiens GN=SSB PE=1 SV=2 | sp\|P05455\|LA_HUMAN (+1) | 47 kDa |
| 21 | Y-box-binding protein 3 OS=Homo sapiens GN=YBX3 PE=1 SV=4 | sp\|P16989\|YBOX3_HUMAN (+1) | 40 kDa |
| 22 | Double-stranded RNA-binding protein Staufen homolog 1 OS=Homo sapiens GN=STAU1 PE=1 SV=2 | sp\|O95793\|STAU1_HUMAN | 63 kDa |
| 23 | Inosine-5'-monophosphate dehydrogenase 2 OS=Homo sapiens GN=IMPDH2 PE=1 SV=2 | sp\|P12268\|IMDH2_HUMAN | 56 kDa |
| 24 | Serine hydroxymethyltransferase, mitochondrial OS=Homo sapiens GN=SHMT2 PE=1 SV=3 | sp\|P34897\|GLYM_HUMAN (+1) | 56 kDa |
| 25 | Protein disulfide-isomerase OS=Homo sapiens GN=P4HB PE=1 SV=3 | sp\|P07237\|PDIA1_HUMAN | 57 kDa |
| 26 | rRNA methyltransferase 3, mitochondrial OS=Homo sapiens GN=MRM3 PE=1 SV=2 | sp\|Q9HC36\|MRM3_HUMAN | 47 kDa |
| 27 | Splicing factor U2AF 65 kDa subunit OS=Homo sapiens GN=U2AF2 PE=1 SV=4 | sp\|P26368\|U2AF2_HUMAN | 54 kDa |
| 28 | D-3-phosphoglycerate dehydrogenase OS=Homo sapiens GN=PHGDH PE=1 SV=4 | sp\|O43175\|SERA_HUMAN | 57 kDa |
| 29 | ATP synthase subunit alpha, mitochondrial OS=Homo sapiens GN=ATP5A1 PE=1 SV=1 | sp\|P25705\|ATPA_HUMAN | 60 kDa |
| 30 | Glucose-6-phosphate isomerase (Fragment) OS=Homo sapiens GN=GPI PE=1 SV=1 | tr\|A0A0A0MTS2\|A0A0A0MTS2_HUMAN | 65 kDa |
| 31 | RuvB-like 1 OS=Homo sapiens GN=RUVBL1 PE=1 SV=1 | sp\|Q9Y265\|RUVB1_HUMAN | 50 kDa |
| 32 | Probable ATP-dependent RNA helicase DDX5 OS=Homo sapiens GN=DDX5 PE=1 SV=1 | sp\|P17844\|DDX5_HUMAN | 69 kDa |
| 33 | U4/U6 small nuclear ribonucleoprotein Prp31 OS=Homo sapiens GN=PRPF31 PE=1 SV=2 | sp\|Q8WWY3\|PRP31_HUMAN | 55 kDa |
| 34 | T-complex protein 1 subunit epsilon OS=Homo sapiens GN=CCT5 PE=1 SV=1 | sp\|P48643\|TCPE_HUMAN (+2) | 60 kDa |
| 35 | Tyrosine--tRNA ligase, cytoplasmic OS=Homo sapiens GN=YARS PE=1 SV=4 | sp\|P54577\|SYYC_HUMAN | 59 kDa |
| 36 | Nucleolar and spindle-associated protein 1 OS=Homo sapiens GN=NUSAP1 PE=1 SV=1 | sp\|Q9BXS6\|NUSAP_HUMAN | 49 kDa |
| 37 | G patch domain-containing protein 4 OS=Homo sapiens GN=GPATCH4 PE=1 SV=2 | sp\|Q5T3I0\|GPTC4_HUMAN | 50 kDa |
| 38 | Vimentin OS=Homo sapiens GN=VIM PE=1 SV=4 | sp\|P08670\|VIME_HUMAN (+1) | 54 kDa |
| 39 | Heterogeneous nuclear ribonucleoprotein M OS=Homo sapiens GN=HNRNPM PE=1 SV=3 | sp\|P52272\|HNRPM_HUMAN | 78 kDa |
| 40 | G-rich sequence factor 1 OS=Homo sapiens GN=GRSF1 PE=1 SV=3 | sp\|Q12849\|GRSF1_HUMAN (+2) | 53 kDa |
| 41 | Nucleolin OS=Homo sapiens GN=NCL PE=1 SV=3 | sp\|P19338\|NUCL_HUMAN (+4) | 77 kDa |
| 42 | Protein DEK OS=Homo sapiens GN=DEK PE=1 SV=1 | sp\|P35659\|DEK_HUMAN | 43 kDa |
| 43 | Lamina-associated polypeptide 2, isoforms beta/gamma OS=Homo sapiens GN=TMPO PE=1 SV=2 | sp\|P42167\|LAP2B_HUMAN (+1) | 51 kDa |
| 44 | Splicing factor 3A subunit 3 OS=Homo sapiens GN=SF3A3 PE=1 SV=1 | sp\|Q12874\|SF3A3_HUMAN (+1) | 59 kDa |
| 45 | Cytosol aminopeptidase OS=Homo sapiens GN=LAP3 PE=1 SV=3 | sp\|P28838\|AMPL_HUMAN (+1) | 56 kDa |
| 46 | Ribosomal oxygenase 2 OS=Homo sapiens GN=RIOX2 PE=1 SV=1 | sp\|Q8IUF8\|RIOX2_HUMAN | 53 kDa |
| 47 | Caseinolytic peptidase B protein homolog OS=Homo sapiens GN=CLPB PE=1 SV=1 | sp\|Q9H078\|CLPB_HUMAN | 79 kDa |
| 48 | Apoptosis inhibitor 5 OS=Homo sapiens GN=API5 PE=1 SV=3 | sp\|Q9BZZ5\|API5_HUMAN | 59 kDa |
| 49 | Bystin OS=Homo sapiens GN=BYSL PE=1 SV=3 | sp\|Q13895\|BYST_HUMAN | 50 kDa |
| 50 | Protein disulfide-isomerase A6 OS=Homo sapiens GN=PDIA6 PE=1 SV=1 | sp\|Q15084\|PDIA6_HUMAN | 48 kDa |
| 51 | cDNA, FLJ93619, highly similar to Homo sapiens PRP4 pre-mRNA processing factor 4 homolog (yeast) (PRPF4), mRNA OS=Homo sapiens PE=2 SV=1 | tr\|B2R7V4\|B2R7V4_HUMAN (+1) | 58 kDa |
| 52 | Regulator of nonsense transcripts 3B OS=Homo sapiens GN=UPF3B PE=1 SV=1 | sp\|Q9BZI7\|REN3B_HUMAN (+1) | 58 kDa |
| 53 | Serine/threonine-protein phosphatase 2A 65 kDa regulatory subunit A alpha isoform OS=Homo sapiens GN=PPP2R1A PE=1 SV=4 | sp\|P30153\|2AAA_HUMAN (+2) | 65 kDa |
| 54 | T-complex protein 1 subunit gamma OS=Homo sapiens GN=CCT3 PE=1 SV=4 | sp\|P49368\|TCPG_HUMAN | 61 kDa |
| 55 | Calreticulin OS=Homo sapiens GN=CALR PE=1 SV=1 | sp\|P27797\|CALR_HUMAN (+2) | 48 kDa |
| 56 | Peptidyl-prolyl cis-trans isomerase FKBP4 OS=Homo sapiens GN=FKBP4 PE=1 SV=3 | sp\|Q02790\|FKBP4_HUMAN | 52 kDa |
| 57 | Heat shock 70 kDa protein 1A OS=Homo sapiens GN=HSPA1A PE=1 SV=1 | sp\|P0DMV8\|HS71A_HUMAN (+2) | 70 kDa |
| 58 | Coiled-coil domain-containing protein 47 OS=Homo sapiens GN=CCDC47 PE=1 SV=1 | sp\|Q96A33\|CCD47_HUMAN | 56 kDa |
| 59 | 26S proteasome regulatory subunit 6B OS=Homo sapiens GN=PSMC4 PE=1 SV=2 | sp\|P43686\|PRS6B_HUMAN (+1) | 47 kDa |
| 60 | Nucleosome assembly protein 1-like 1 OS=Homo sapiens GN=NAP1L1 PE=1 SV=1 | sp\|P55209\|NP1L1_HUMAN (+3) | 45 kDa |

| # | Identified Proteins (716) | Accession Number | Molecular  weight |
| --- | --- | --- | --- |
| 61 | Double-stranded RNA-binding protein Staufen homolog 2 OS=Homo sapiens GN=STAU2 PE=1 SV=1 | sp\|Q9NUL3\|STAU2_HUMAN (+2) | 63 kDa |
| 62 | Phenylalanine--tRNA ligase alpha subunit OS=Homo sapiens GN=FARSA PE=1 SV=3 | sp\|Q9Y285\|SYFA_HUMAN (+2) | 58 kDa |
| 63 | Aldehyde dehydrogenase X, mitochondrial OS=Homo sapiens GN=ALDH1B1 PE=1 SV=3 | sp\|P30837\|AL1B1_HUMAN | 57 kDa |
| 64 | Procollagen-proline, 2-oxoglutarate 4-dioxygenase (Proline 4-hydroxylase), alpha polypeptide I variant (Fragment) OS=Homo sapiens GN=P4HA1 PE=2 SV=1 | tr\|Q5VSQ6\|Q5VSQ6_HUMAN | 61 kDa |
| 65 | Protein FAM98A OS=Homo sapiens GN=FAM98A PE=1 SV=1 | sp\|Q8NCA5\|FA98A_HUMAN (+1) | 55 kDa |
| 66 | Luc7-like protein 3 OS=Homo sapiens GN=LUC7L3 PE=1 SV=2 | sp\|O95232\|LC7L3_HUMAN (+2) | 51 kDa |
| 67 | Heterogeneous nuclear ribonucleoprotein H2 OS=Homo sapiens GN=HNRNPH2 PE=1 SV=1 | sp\|P55795\|HNRH2_HUMAN | 49 kDa |
| 68 | T-complex protein 1 subunit delta OS=Homo sapiens GN=CCT4 PE=1 SV=4 | sp\|P50991\|TCPD_HUMAN | 58 kDa |
| 69 | Insulin-like growth factor 2 mRNA-binding protein 1 OS=Homo sapiens GN=IGF2BP1 PE=1 SV=2 | sp\|Q9NZI8\|IF2B1_HUMAN | 63 kDa |
| 70 | RuvB-like 2 OS=Homo sapiens GN=RUVBL2 PE=1 SV=3 | sp\|Q9Y230\|RUVB2_HUMAN | 51 kDa |
| 71 | Histidine--tRNA ligase, cytoplasmic OS=Homo sapiens GN=HARS PE=1 SV=2 | sp\|P12081\|SYHC_HUMAN | 57 kDa |
| 72 | DnaJ homolog subfamily C member 7 OS=Homo sapiens GN=DNAJC7 PE=1 SV=2 | sp\|Q99615\|DNJC7_HUMAN | 56 kDa |
| 73 | T-complex protein 1 subunit zeta OS=Homo sapiens GN=CCT6A PE=1 SV=3 | sp\|P40227\|TCPZ_HUMAN (+3) | 58 kDa |
| 74 | 40S ribosomal protein SA OS=Homo sapiens GN=RPSA PE=1 SV=4 | sp\|P08865\|RSSA_HUMAN (+3) | 33 kDa |
| 75 | ATPase family AAA domain-containing protein 3A (Fragment) OS=Homo sapiens GN=ATAD3A PE=1 SV=1 | tr\|H0Y2W2\|H0Y2W2_HUMAN | 64 kDa |
| 76 | Proteasome (Prosome, macropain) 26S subunit, ATPase, 1 OS=Homo sapiens GN=PSMC1 PE=2 SV=1 | tr\|Q53XL8\|Q53XL8_HUMAN (+1) | 49 kDa |
| 77 | Lysine-rich nucleolar protein 1 OS=Homo sapiens GN=KNOP1 PE=1 SV=1 | sp\|Q1ED39\|KNOP1_HUMAN | 52 kDa |
| 78 | Eukaryotic translation initiation factor 2 subunit 2 OS=Homo sapiens GN=EIF2S2 PE=1 SV=2 | sp\|P20042\|IF2B_HUMAN (+1) | 38 kDa |
| 79 | 26S proteasome regulatory subunit 6A OS=Homo sapiens GN=PSMC3 PE=1 SV=3 | sp\|P17980\|PRS6A_HUMAN (+2) | 49 kDa |
| 80 | Eukaryotic translation initiation factor 5 OS=Homo sapiens GN=EIF5 PE=1 SV=2 | sp\|P55010\|IF5_HUMAN | 49 kDa |
| 81 | Nucleolar complex protein 4 homolog OS=Homo sapiens GN=NOC4L PE=1 SV=1 | sp\|Q9BVI4\|NOC4L_HUMAN | 58 kDa |
| 82 | Heterogeneous nuclear ribonucleoprotein D0 OS=Homo sapiens GN=HNRNPD PE=1 SV=1 | sp\|Q14103\|HNRPD_HUMAN (+2) | 38 kDa |
| 83 | cAMP-dependent protein kinase type II-alpha regulatory subunit OS=Homo sapiens GN=PRKAR2A PE=1 SV=2 | sp\|P13861\|KAP2_HUMAN (+3) | 46 kDa |
| 84 | Splicing factor 45 OS=Homo sapiens GN=RBM17 PE=1 SV=1 | sp\|Q96I25\|SPF45_HUMAN (+1) | 45 kDa |
| 85 | Testis-specific Y-encoded-like protein 1 OS=Homo sapiens GN=TSPYL1 PE=1 SV=3 | sp\|Q9H0U9\|TSYL1_HUMAN | 49 kDa |
| 86 | Insulin-like growth factor 2 mRNA-binding protein 3 OS=Homo sapiens GN=IGF2BP3 PE=1 SV=2 | sp\|O00425\|IF2B3_HUMAN | 64 kDa |
| 87 | Heterogeneous nuclear ribonucleoprotein Q OS=Homo sapiens GN=SYNCRIP PE=1 SV=2 | sp\|O60506\|HNRPQ_HUMAN (+1) | 70 kDa |
| 88 | Serine beta-lactamase-like protein LACTB, mitochondrial OS=Homo sapiens GN=LACTB PE=1 SV=2 | sp\|P83111\|LACTB_HUMAN | 61 kDa |
| 89 | FAS-associated factor 2 OS=Homo sapiens GN=FAF2 PE=1 SV=2 | sp\|Q96CS3\|FAF2_HUMAN | 53 kDa |
| 90 | Guanine nucleotide binding protein-like 3 (Nucleolar), isoform CRA_b OS=Homo sapiens GN=GNL3 PE=4 SV=1 | tr\|A0A024R2Z6\|A0A024R2Z6_HUMAN | 61 kDa |
| 91 | Heterogeneous nuclear ribonucleoprotein F OS=Homo sapiens GN=HNRNPF PE=1 SV=3 | sp\|P52597\|HNRPF_HUMAN (+1) | 46 kDa |
| 92 | THUMP domain-containing protein 1 OS=Homo sapiens GN=THUMPD1 PE=1 SV=2 | sp\|Q9NXG2\|THUM1_HUMAN (+2) | 39 kDa |
| 93 | Proliferation-associated protein 2G4 OS=Homo sapiens GN=PA2G4 PE=1 SV=3 | sp\|Q9UQ80\|PA2G4_HUMAN (+1) | 44 kDa |
| 94 | Thioredoxin domain-containing protein 5 OS=Homo sapiens GN=TXNDC5 PE=1 SV=2 | sp\|Q8NBS9\|TXND5_HUMAN | 48 kDa |
| 95 | Heat shock cognate 71 kDa protein OS=Homo sapiens GN=HSPA8 PE=1 SV=1 | sp\|P11142\|HSP7C_HUMAN | 71 kDa |
| 96 | Polyadenylate-binding protein 1 OS=Homo sapiens GN=PABPC1 PE=1 SV=2 | sp\|P11940\|PABP1_HUMAN (+2) | 71 kDa |
| 97 | Eukaryotic translation initiation factor 3 subunit E OS=Homo sapiens GN=EIF3E PE=1 SV=1 | sp\|P60228\|EIF3E_HUMAN | 52 kDa |
| 98 | Ribosomal RNA processing protein 1 homolog A OS=Homo sapiens GN=RRP1 PE=1 SV=1 | sp\|P56182\|RRP1_HUMAN (+1) | 53 kDa |
| 99 | Tubulin beta-4B chain OS=Homo sapiens GN=TUBB4B PE=1 SV=1 | sp\|P68371\|TBB4B_HUMAN | 50 kDa |
| 100 | Pre-mRNA-splicing regulator WTAP OS=Homo sapiens GN=WTAP PE=1 SV=2 | sp\|Q15007\|FL2D_HUMAN | 44 kDa |
| 101 | Far upstream element-binding protein 3 OS=Homo sapiens GN=FUBP3 PE=1 SV=2 | sp\|Q96I24\|FUBP3_HUMAN (+1) | 62 kDa |
| 102 | Putative RNA-binding protein Luc7-like 2 OS=Homo sapiens GN=LUC7L2 PE=1 SV=2 | sp\|Q9Y383\|LC7L2_HUMAN | 47 kDa |
| 103 | Actin-related protein 3 OS=Homo sapiens GN=ACTR3 PE=1 SV=3 | sp\|P61158\|ARP3_HUMAN (+1) | 47 kDa |
| 104 | Non-POU domain-containing octamer-binding protein OS=Homo sapiens GN=NONO PE=1 SV=4 | sp\|Q15233\|NONO_HUMAN (+1) | 54 kDa |
| 105 | Importin subunit alpha-7 OS=Homo sapiens GN=KPNA6 PE=1 SV=1 | sp\|O60684\|IMA7_HUMAN | 60 kDa |
| 106 | Histone-binding protein RBBP4 OS=Homo sapiens GN=RBBP4 PE=1 SV=3 | sp\|Q09028\|RBBP4_HUMAN | 48 kDa |
| 107 | Nicotinamide phosphoribosyltransferase OS=Homo sapiens GN=NAMPT PE=1 SV=1 | sp\|P43490\|NAMPT_HUMAN (+1) | 56 kDa |
| 108 | Bcl-2-associated transcription factor 1 OS=Homo sapiens GN=BCLAF1 PE=1 SV=2 | sp\|Q9NYF8\|BCLF1_HUMAN | 106 kDa |
| 109 | Nucleophosmin OS=Homo sapiens GN=NPM1 PE=1 SV=2 | sp\|P06748\|NPM_HUMAN | 33 kDa |
| 110 | U1 small nuclear ribonucleoprotein 70 kDa OS=Homo sapiens GN=SNRNP70 PE=1 SV=2 | sp\|P08621\|RU17_HUMAN (+1) | 52 kDa |
| 111 | Annexin A11 OS=Homo sapiens GN=ANXA11 PE=1 SV=1 | sp\|P50995\|ANX11_HUMAN (+1) | 54 kDa |
| 112 | Aldehyde dehydrogenase, mitochondrial OS=Homo sapiens GN=ALDH2 PE=1 SV=2 | sp\|P05091\|ALDH2_HUMAN | 56 kDa |
| 113 | Coiled-coil domain-containing protein 86 OS=Homo sapiens GN=CCDC86 PE=1 SV=1 | sp\|Q9H6F5\|CCD86_HUMAN | 40 kDa |
| 114 | Glutamate dehydrogenase 1, mitochondrial OS=Homo sapiens GN=GLUD1 PE=1 SV=2 | sp\|P00367\|DHE3_HUMAN (+2) | 61 kDa |
| 115 | Polymerase delta-interacting protein 3 OS=Homo sapiens GN=POLDIP3 PE=1 SV=2 | sp\|Q9BY77\|PDIP3_HUMAN (+2) | 46 kDa |
| 116 | Heterogeneous nuclear ribonucleoprotein A1 OS=Homo sapiens GN=HNRNPA1 PE=1 SV=5 | sp\|P09651\|ROA1_HUMAN (+4) | 39 kDa |
| 117 | Signal recognition particle 54 kDa protein OS=Homo sapiens GN=SRP54 PE=1 SV=1 | sp\|P61011\|SRP54_HUMAN | 56 kDa |
| 118 | Ras-GTPase activating protein SH3 domain-binding protein 2, isoform CRA_b OS=Homo sapiens GN=G3BP2 PE=4 SV=1 | tr\|A0A024RDB2\|A0A024RDB2_HUMAN | 51 kDa |
| 119 | Eukaryotic translation initiation factor 3 subunit F OS=Homo sapiens GN=EIF3F PE=1 SV=1 | sp\|O00303\|EIF3F_HUMAN (+2) | 38 kDa |
| 120 | Serine--tRNA ligase, cytoplasmic OS=Homo sapiens GN=SARS PE=1 SV=3 | sp\|P49591\|SYSC_HUMAN (+2) | 59 kDa |

| # | Identified Proteins (716) | Accession Number | Molecular  weight |
| --- | --- | --- | --- |
| 121 | Centromere protein U OS=Homo sapiens GN=CENPU PE=1 SV=1 | sp\|Q71F23\|CENPU_HUMAN | 48 kDa |
| 122 | Tryptophan--tRNA ligase, cytoplasmic OS=Homo sapiens GN=WARS PE=1 SV=2 | sp\|P23381\|SYWC_HUMAN (+1) | 53 kDa |
| 123 | Tubulin-specific chaperone E OS=Homo sapiens GN=TBCE PE=1 SV=1 | sp\|Q15813\|TBCE_HUMAN | 59 kDa |
| 124 | Glutaminase kidney isoform, mitochondrial OS=Homo sapiens GN=GLS PE=1 SV=1 | sp\|O94925\|GLSK_HUMAN (+1) | 73 kDa |
| 125 | Basigin OS=Homo sapiens GN=BSG PE=1 SV=2 | sp\|P35613\|BASI_HUMAN (+2) | 42 kDa |
| 126 | Spliceosome RNA helicase DDX39B OS=Homo sapiens GN=DDX39B PE=1 SV=1 | sp\|Q13838\|DX39B_HUMAN | 49 kDa |
| 127 | ATP-dependent zinc metalloprotease YME1L1 OS=Homo sapiens GN=YME1L1 PE=1 SV=2 | sp\|Q96TA2\|YMEL1_HUMAN | 86 kDa |
| 128 | Cellular tumor antigen p53 (Fragment) OS=Homo sapiens PE=2 SV=1 | tr\|H6U5S2\|H6U5S2_HUMAN | 44 kDa |
| 129 | Hsc70-interacting protein OS=Homo sapiens GN=ST13 PE=1 SV=2 | sp\|P50502\|F10A1_HUMAN (+2) | 41 kDa |
| 130 | Hsp90 co-chaperone Cdc37 OS=Homo sapiens GN=CDC37 PE=1 SV=1 | sp\|Q16543\|CDC37_HUMAN (+1) | 44 kDa |
| 131 | Cleavage and polyadenylation specificity factor subunit 7 OS=Homo sapiens GN=CPSF7 PE=1 SV=1 | sp\|Q8N684\|CPSF7_HUMAN (+1) | 52 kDa |
| 132 | Mitochondrial-processing peptidase subunit alpha OS=Homo sapiens GN=PMPCA PE=1 SV=2 | sp\|Q10713\|MPPA_HUMAN | 58 kDa |
| 133 | Adenylosuccinate lyase OS=Homo sapiens GN=ADSL PE=1 SV=2 | sp\|P30566\|PUR8_HUMAN (+2) | 55 kDa |
| 134 | Serine/threonine-protein phosphatase 5 OS=Homo sapiens GN=PPP5C PE=1 SV=1 | sp\|P53041\|PPP5_HUMAN (+5) | 57 kDa |
| 135 | Torsin-1A-interacting protein 1 OS=Homo sapiens GN=TOR1AIP1 PE=1 SV=1 | tr\|J3KN66\|J3KN66_HUMAN | 68 kDa |
| 136 | Cytoplasmic dynein 1 light intermediate chain 1 OS=Homo sapiens GN=DYNC1LI1 PE=1 SV=3 | sp\|Q9Y6G9\|DC1L1_HUMAN | 57 kDa |
| 137 | Importin subunit alpha-4 OS=Homo sapiens GN=KPNA3 PE=1 SV=2 | sp\|O00505\|IMA4_HUMAN (+1) | 58 kDa |
| 138 | NF-kappa-B-activating protein OS=Homo sapiens GN=NKAP PE=1 SV=1 | sp\|Q8N5F7\|NKAP_HUMAN | 47 kDa |
| 139 | G patch domain and KOW motifs-containing protein OS=Homo sapiens GN=GPKOW PE=1 SV=2 | sp\|Q92917\|GPKOW_HUMAN | 52 kDa |
| 140 | Cell growth-regulating nucleolar protein OS=Homo sapiens GN=LYAR PE=1 SV=2 | sp\|Q9NX58\|LYAR_HUMAN | 44 kDa |
| 141 | Prelamin-A/C OS=Homo sapiens GN=LMNA PE=1 SV=1 | sp\|P02545\|LMNA_HUMAN | 74 kDa |
| 142 | Probable ATP-dependent RNA helicase DDX17 OS=Homo sapiens GN=DDX17 PE=1 SV=2 | sp\|Q92841\|DDX17_HUMAN (+1) | 80 kDa |
| 143 | Ras GTPase-activating protein-binding protein 1 OS=Homo sapiens GN=G3BP1 PE=1 SV=1 | sp\|Q13283\|G3BP1_HUMAN (+2) | 52 kDa |
| 144 | Serine/threonine-protein kinase 26 OS=Homo sapiens GN=STK26 PE=1 SV=2 | sp\|Q9P289\|STK26_HUMAN | 47 kDa |
| 145 | Serine--tRNA ligase, mitochondrial OS=Homo sapiens GN=SARS2 PE=1 SV=1 | sp\|Q9NP81\|SYSM_HUMAN (+1) | 58 kDa |
| 146 | Phosphoglucomutase-1 OS=Homo sapiens GN=PGM1 PE=1 SV=3 | sp\|P36871\|PGM1_HUMAN | 61 kDa |
| 147 | Programmed cell death protein 7 OS=Homo sapiens GN=PDCD7 PE=1 SV=1 | sp\|Q8N8D1\|PDCD7_HUMAN (+1) | 55 kDa |
| 148 | Pre-mRNA-splicing factor CWC25 homolog OS=Homo sapiens GN=CWC25 PE=1 SV=1 | sp\|Q9NXE8\|CWC25_HUMAN | 50 kDa |
| 149 | MAGUK p55 subfamily member 6 OS=Homo sapiens GN=MPP6 PE=1 SV=2 | sp\|Q9NZW5\|MPP6_HUMAN (+1) | 61 kDa |
| 150 | Heterogeneous nuclear ribonucleoprotein D-like OS=Homo sapiens GN=HNRNPDL PE=1 SV=3 | sp\|O14979\|HNRDL_HUMAN (+1) | 46 kDa |
| 151 | Thyroid hormone receptor-associated protein 3 OS=Homo sapiens GN=THRAP3 PE=1 SV=2 | sp\|Q9Y2W1\|TR150_HUMAN | 109 kDa |
| 152 | RNA-binding protein FUS OS=Homo sapiens GN=FUS PE=1 SV=1 | sp\|P35637\|FUS_HUMAN (+5) | 53 kDa |
| 153 | Rap1 GTPase-GDP dissociation stimulator 1 OS=Homo sapiens GN=RAP1GDS1 PE=1 SV=3 | sp\|P52306\|GDS1_HUMAN | 66 kDa |
| 154 | Dynactin subunit 2 OS=Homo sapiens GN=DCTN2 PE=1 SV=4 | sp\|Q13561\|DCTN2_HUMAN (+1) | 44 kDa |
| 155 | Brain-specific angiogenesis inhibitor 1-associated protein 2 OS=Homo sapiens GN=BAIAP2 PE=1 SV=1 | sp\|Q9UQB8\|BAIP2_HUMAN (+1) | 61 kDa |
| 156 | NF110b OS=Homo sapiens PE=2 SV=1 | tr\|F4ZW66\|F4ZW66_HUMAN | 96 kDa |
| 157 | COP9 signalosome complex subunit 2 OS=Homo sapiens GN=COPS2 PE=1 SV=1 | sp\|P61201\|CSN2_HUMAN (+1) | 52 kDa |
| 158 | Zinc finger CCCH domain-containing protein 15 OS=Homo sapiens GN=ZC3H15 PE=1 SV=1 | sp\|Q8WU90\|ZC3HF_HUMAN (+1) | 49 kDa |
| 159 | p4HA2 protein OS=Homo sapiens GN=P4HA2 PE=2 SV=1 | tr\|Q05DA4\|Q05DA4_HUMAN | 57 kDa |
| 160 | NEDD8-activating enzyme E1 catalytic subunit OS=Homo sapiens GN=UBA3 PE=1 SV=2 | sp\|Q8TBC4\|UBA3_HUMAN (+1) | 52 kDa |
| 161 | Nucleosome assembly protein 1-like 4 OS=Homo sapiens GN=NAP1L4 PE=1 SV=1 | sp\|Q99733\|NP1L4_HUMAN (+1) | 43 kDa |
| 162 | Tubulin beta-6 chain OS=Homo sapiens GN=TUBB6 PE=1 SV=1 | sp\|Q9BUF5\|TBB6_HUMAN | 50 kDa |
| 163 | Keratin, type I cytoskeletal 10 OS=Homo sapiens GN=KRT10 PE=1 SV=6 | sp\|P13645\|K1C10_HUMAN | 59 kDa |
| 164 | Bifunctional purine biosynthesis protein PURH OS=Homo sapiens GN=ATIC PE=1 SV=3 | sp\|P31939\|PUR9_HUMAN (+1) | 65 kDa |
| 165 | Programmed cell death protein 4 OS=Homo sapiens GN=PDCD4 PE=1 SV=2 | sp\|Q53EL6\|PDCD4_HUMAN (+1) | 52 kDa |
| 166 | Arginine/serine-rich coiled-coil protein 2 OS=Homo sapiens GN=RSRC2 PE=1 SV=1 | sp\|Q7L4I2\|RSRC2_HUMAN | 51 kDa |
| 167 | Vacuolar protein sorting-associated protein 4A OS=Homo sapiens GN=VPS4A PE=1 SV=1 | sp\|Q9UN37\|VPS4A_HUMAN (+1) | 49 kDa |
| 168 | Cytochrome b-c1 complex subunit 1, mitochondrial OS=Homo sapiens GN=UQCRC1 PE=1 SV=3 | sp\|P31930\|QCR1_HUMAN | 53 kDa |
| 169 | Alpha-aminoadipic semialdehyde dehydrogenase OS=Homo sapiens GN=ALDH7A1 PE=1 SV=5 | sp\|P49419\|AL7A1_HUMAN (+2) | 58 kDa |
| 170 | Serum response factor-binding protein 1 OS=Homo sapiens GN=SRFBP1 PE=1 SV=1 | sp\|Q8NEF9\|SRFB1_HUMAN | 49 kDa |
| 171 | RNA-binding protein 42 OS=Homo sapiens GN=RBM42 PE=1 SV=1 | sp\|Q9BTD8\|RBM42_HUMAN | 50 kDa |
| 172 | Heterogeneous nuclear ribonucleoprotein L OS=Homo sapiens GN=HNRNPL PE=1 SV=2 | sp\|P14866\|HNRPL_HUMAN (+2) | 64 kDa |
| 173 | Tubulin beta-2B chain OS=Homo sapiens GN=TUBB2B PE=1 SV=1 | sp\|Q9BVA1\|TBB2B_HUMAN | 50 kDa |
| 174 | EH domain-containing protein 1 OS=Homo sapiens GN=EHD1 PE=1 SV=2 | sp\|Q9H4M9\|EHD1_HUMAN (+4) | 61 kDa |
| 175 | Keratin 1 OS=Homo sapiens GN=KRT1 PE=3 SV=1 | tr\|H6VRF8\|H6VRF8_HUMAN | 66 kDa |
| 176 | Elongation factor 1-alpha 1 OS=Homo sapiens GN=EEF1A1 PE=1 SV=1 | sp\|P68104\|EF1A1_HUMAN (+2) | 50 kDa |
| 177 | Poly(U)-binding-splicing factor PUF60 (Fragment) OS=Homo sapiens GN=PUF60 PE=1 SV=1 | tr\|A0A0J9YYL3\|A0A0J9YYL3_HUMAN | 55 kDa |
| 178 | General transcription factor IIH subunit 1 OS=Homo sapiens GN=GTF2H1 PE=1 SV=1 | sp\|P32780\|TF2H1_HUMAN (+1) | 62 kDa |
| 179 | Succinyl-CoA:3-ketoacid coenzyme A transferase 1, mitochondrial OS=Homo sapiens GN=OXCT1 PE=1 SV=1 | sp\|P55809\|SCOT1_HUMAN (+1) | 56 kDa |
| 180 | Probable glutamate--tRNA ligase, mitochondrial OS=Homo sapiens GN=EARS2 PE=1 SV=2 | sp\|Q5JPH6\|SYEM_HUMAN | 59 kDa |

| # | Identified Proteins (716) | Accession Number | Molecular  weight |
| --- | --- | --- | --- |
| 181 | Protein LSM14 homolog B OS=Homo sapiens GN=LSM14B PE=1 SV=1 | sp\|Q9BX40\|LS14B_HUMAN | 42 kDa |
| 182 | Lipoamide acyltransferase component of branched-chain alpha-keto acid dehydrogenase complex, mitochondrial OS=Homo sapiens GN=DBT PE=1 SV=3 | sp\|P11182\|ODB2_HUMAN | 53 kDa |
| 183 | S-adenosylmethionine synthase isoform type-2 OS=Homo sapiens GN=MAT2A PE=1 SV=1 | sp\|P31153\|METK2_HUMAN (+1) | 44 kDa |
| 184 | RNA exonuclease 4 OS=Homo sapiens GN=REXO4 PE=1 SV=2 | sp\|Q9GZR2\|REXO4_HUMAN | 47 kDa |
| 185 | Ribosome biogenesis protein NOP53 OS=Homo sapiens GN=NOP53 PE=1 SV=2 | sp\|Q9NZM5\|NOP53_HUMAN (+4) | 54 kDa |
| 186 | cAMP-dependent protein kinase type I-alpha regulatory subunit OS=Homo sapiens GN=PRKAR1A PE=1 SV=1 | sp\|P10644\|KAP0_HUMAN (+1) | 43 kDa |
| 187 | COP9 signalosome complex subunit 1 OS=Homo sapiens GN=GPS1 PE=1 SV=4 | sp\|Q13098\|CSN1_HUMAN (+1) | 56 kDa |
| 188 | Carboxypeptidase OS=Homo sapiens PE=2 SV=1 | tr\|B3KW79\|B3KW79_HUMAN | 54 kDa |
| 189 | HSP90AA1 protein (Fragment) OS=Homo sapiens GN=HSP90AA1 PE=2 SV=1 | tr\|Q2VPJ6\|Q2VPJ6_HUMAN (+1) | 68 kDa |
| 190 | Ubiquitin carboxyl-terminal hydrolase 14 OS=Homo sapiens GN=USP14 PE=1 SV=3 | sp\|P54578\|UBP14_HUMAN | 56 kDa |
| 191 | Serine/threonine-protein kinase 4 OS=Homo sapiens GN=STK4 PE=1 SV=2 | sp\|Q13043\|STK4_HUMAN (+1) | 56 kDa |
| 192 | Serine/threonine-protein kinase PAK 2 OS=Homo sapiens GN=PAK2 PE=1 SV=3 | sp\|Q13177\|PAK2_HUMAN (+1) | 58 kDa |
| 193 | Histone-binding protein RBBP7 OS=Homo sapiens GN=RBBP7 PE=1 SV=1 | sp\|Q16576\|RBBP7_HUMAN (+1) | 48 kDa |
| 194 | Insulin-like growth factor 2 mRNA-binding protein 2 OS=Homo sapiens GN=IGF2BP2 PE=1 SV=2 | sp\|Q9Y6M1\|IF2B2_HUMAN (+1) | 66 kDa |
| 195 | G2/mitotic-specific cyclin-B1 OS=Homo sapiens GN=CCNB1 PE=1 SV=1 | sp\|P14635\|CCNB1_HUMAN | 48 kDa |
| 196 | Catalase OS=Homo sapiens GN=CAT PE=1 SV=3 | sp\|P04040\|CATA_HUMAN | 60 kDa |
| 197 | Tubulin alpha-1C chain OS=Homo sapiens GN=TUBA1C PE=1 SV=1 | sp\|Q9BQE3\|TBA1C_HUMAN | 50 kDa |
| 198 | Glutathione synthetase OS=Homo sapiens GN=GSS PE=1 SV=1 | sp\|P48637\|GSHB_HUMAN (+1) | 52 kDa |
| 199 | Interleukin enhancer-binding factor 2 OS=Homo sapiens GN=ILF2 PE=1 SV=2 | sp\|Q12905\|ILF2_HUMAN (+3) | 43 kDa |
| 200 | Plastin-3 OS=Homo sapiens GN=PLS3 PE=1 SV=4 | sp\|P13797\|PLST_HUMAN (+3) | 71 kDa |
| 201 | 40S ribosomal protein S3 OS=Homo sapiens GN=RPS3 PE=1 SV=2 | sp\|P23396\|RS3_HUMAN | 27 kDa |
| 202 | KH domain-containing, RNA-binding, signal transduction-associated protein 1 OS=Homo sapiens GN=KHDRBS1 PE=1 SV=1 | sp\|Q07666\|KHDR1_HUMAN | 48 kDa |
| 203 | RNA-binding protein 39 OS=Homo sapiens GN=RBM39 PE=1 SV=2 | sp\|Q14498\|RBM39_HUMAN (+2) | 59 kDa |
| 204 | Zinc finger protein 622 OS=Homo sapiens GN=ZNF622 PE=1 SV=1 | sp\|Q969S3\|ZN622_HUMAN | 54 kDa |
| 205 | SWI/SNF-related matrix-associated actin-dependent regulator of chromatin subfamily D member 1 OS=Homo sapiens GN=SMARCD1 PE=1 SV=2 | sp\|Q96GM5\|SMRD1_HUMAN (+1) | 58 kDa |
| 206 | Target of EGR1 protein 1 OS=Homo sapiens GN=TOE1 PE=1 SV=1 | sp\|Q96GM8\|TOE1_HUMAN | 57 kDa |
| 207 | KRR1 small subunit processome component homolog OS=Homo sapiens GN=KRR1 PE=1 SV=4 | sp\|Q13601\|KRR1_HUMAN | 44 kDa |
| 208 | Peptidase M20 domain-containing protein 2 OS=Homo sapiens GN=PM20D2 PE=1 SV=2 | sp\|Q8IYS1\|P20D2_HUMAN | 48 kDa |
| 209 | Protein kinase C and casein kinase substrate in neurons protein 3 OS=Homo sapiens GN=PACSIN3 PE=1 SV=2 | sp\|Q9UKS6\|PACN3_HUMAN | 48 kDa |
| 210 | ATP-dependent RNA helicase DDX3X OS=Homo sapiens GN=DDX3X PE=1 SV=1 | tr\|A0A0D9SFB3\|A0A0D9SFB3_HUMAN (+1) | 71 kDa |
| 211 | Splicing factor 3B subunit 4 OS=Homo sapiens GN=SF3B4 PE=1 SV=1 | sp\|Q15427\|SF3B4_HUMAN | 44 kDa |
| 212 | Katanin p60 ATPase-containing subunit A1 OS=Homo sapiens GN=KATNA1 PE=1 SV=1 | sp\|O75449\|KTNA1_HUMAN (+1) | 56 kDa |
| 213 | Tubulin beta-4A chain OS=Homo sapiens GN=TUBB4A PE=1 SV=2 | sp\|P04350\|TBB4A_HUMAN | 50 kDa |
| 214 | DNA primase large subunit OS=Homo sapiens GN=PRIM2 PE=1 SV=2 | sp\|P49643\|PRI2_HUMAN | 59 kDa |
| 215 | SWI/SNF-related matrix-associated actin-dependent regulator of chromatin subfamily E member 1 OS=Homo sapiens GN=SMARCE1 PE=1 SV=2 | sp\|Q969G3\|SMCE1_HUMAN (+1) | 47 kDa |
| 216 | Alpha-ketoglutarate-dependent dioxygenase FTO OS=Homo sapiens GN=FTO PE=1 SV=3 | sp\|Q9C0B1\|FTO_HUMAN (+1) | 58 kDa |
| 217 | SAP30-binding protein OS=Homo sapiens GN=SAP30BP PE=1 SV=1 | sp\|Q9UHR5\|S30BP_HUMAN (+2) | 34 kDa |
| 218 | Beta-hexosaminidase OS=Homo sapiens PE=2 SV=1 | tr\|B4DVA7\|B4DVA7_HUMAN (+1) | 62 kDa |
| 219 | Cdc42 effector protein 1 OS=Homo sapiens GN=CDC42EP1 PE=1 SV=1 | sp\|Q00587\|BORG5_HUMAN (+1) | 40 kDa |
| 220 | E3 ubiquitin-protein ligase RING1 OS=Homo sapiens GN=RING1 PE=1 SV=2 | sp\|Q06587\|RING1_HUMAN (+2) | 42 kDa |
| 221 | Periphilin-1 OS=Homo sapiens GN=PPHLN1 PE=1 SV=2 | sp\|Q8NEY8\|PPHLN_HUMAN | 53 kDa |
| 222 | Nuclear pore complex protein Nup50 OS=Homo sapiens GN=NUP50 PE=1 SV=2 | sp\|Q9UKX7\|NUP50_HUMAN | 50 kDa |
| 223 | Pre-mRNA-processing factor 19 OS=Homo sapiens GN=PRPF19 PE=1 SV=1 | sp\|Q9UMS4\|PRP19_HUMAN | 55 kDa |
| 224 | Rab11 family-interacting protein 2 OS=Homo sapiens GN=RAB11FIP2 PE=1 SV=1 | sp\|Q7L804\|RFIP2_HUMAN | 58 kDa |
| 225 | Nucleoporin p54 OS=Homo sapiens GN=NUP54 PE=1 SV=2 | sp\|Q7Z3B4\|NUP54_HUMAN (+2) | 55 kDa |
| 226 | Polyadenylate-binding protein 2 OS=Homo sapiens GN=PABPN1 PE=1 SV=3 | sp\|Q86U42\|PABP2_HUMAN | 33 kDa |
| 227 | Stress-70 protein, mitochondrial OS=Homo sapiens GN=HSPA9 PE=1 SV=2 | sp\|P38646\|GRP75_HUMAN | 74 kDa |
| 228 | Rho GTPase-activating protein 1 OS=Homo sapiens GN=ARHGAP1 PE=1 SV=1 | sp\|Q07960\|RHG01_HUMAN | 50 kDa |
| 229 | Protein TFG OS=Homo sapiens GN=TFG PE=1 SV=2 | sp\|Q92734\|TFG_HUMAN (+5) | 43 kDa |
| 230 | CUGBP Elav-like family member 1 OS=Homo sapiens GN=CELF1 PE=1 SV=2 | sp\|Q92879\|CELF1_HUMAN (+1) | 52 kDa |
| 231 | Reticulon OS=Homo sapiens GN=RTN4 PE=1 SV=1 | tr\|F8W914\|F8W914_HUMAN (+1) | 37 kDa |
| 232 | 26S proteasome non-ATPase regulatory subunit 12 OS=Homo sapiens GN=PSMD12 PE=1 SV=3 | sp\|O00232\|PSD12_HUMAN (+1) | 53 kDa |
| 233 | Dihydrolipoyllysine-residue succinyltransferase component of 2-oxoglutarate dehydrogenase complex, mitochondrial OS=Homo sapiens GN=DLST PE=1 SV=4 | sp\|P36957\|ODO2_HUMAN (+1) | 49 kDa |
| 234 | Angio-associated migratory cell protein OS=Homo sapiens GN=AAMP PE=1 SV=2 | sp\|Q13685\|AAMP_HUMAN (+3) | 47 kDa |
| 235 | Rho GTPase-activating protein 19 OS=Homo sapiens GN=ARHGAP19 PE=1 SV=1 | sp\|Q14CB8\|RHG19_HUMAN | 56 kDa |
| 236 | THAP domain-containing protein 11 OS=Homo sapiens GN=THAP11 PE=1 SV=2 | sp\|Q96EK4\|THA11_HUMAN | 34 kDa |
| 237 | Cell division cycle 5-like protein OS=Homo sapiens GN=CDC5L PE=1 SV=2 | sp\|Q99459\|CDC5L_HUMAN (+1) | 92 kDa |
| 238 | Sorting nexin-6 OS=Homo sapiens GN=SNX6 PE=1 SV=1 | sp\|Q9UNH7\|SNX6_HUMAN (+2) | 47 kDa |
| 239 | Synoviocyte proliferation-associated in collagen-induced arthritis 1 OS=Homo sapiens GN=SPACIA1 PE=2 SV=1 | tr\|G1UCX3\|G1UCX3_HUMAN | 54 kDa |
| 240 | Importin subunit alpha-3 OS=Homo sapiens GN=KPNA4 PE=1 SV=1 | sp\|O00629\|IMA3_HUMAN | 58 kDa |

| # | Identified Proteins (716) | Accession Number | Molecular  weight |
| --- | --- | --- | --- |
| 241 | Alpha-galactosidase A OS=Homo sapiens GN=GLA PE=1 SV=1 | sp\|P06280\|AGAL_HUMAN (+1) | 49 kDa |
| 242 | Regulator of chromosome condensation OS=Homo sapiens GN=RCC1 PE=1 SV=1 | sp\|P18754\|RCC1_HUMAN (+3) | 45 kDa |
| 243 | 26S proteasome non-ATPase regulatory subunit 4 OS=Homo sapiens GN=PSMD4 PE=1 SV=1 | sp\|P55036\|PSMD4_HUMAN | 41 kDa |
| 244 | Eukaryotic initiation factor 4A-I OS=Homo sapiens GN=EIF4A1 PE=1 SV=1 | sp\|P60842\|IF4A1_HUMAN | 46 kDa |
| 245 | DnaJ homolog subfamily C member 3 OS=Homo sapiens GN=DNAJC3 PE=1 SV=1 | sp\|Q13217\|DNJC3_HUMAN (+1) | 58 kDa |
| 246 | Centrosomal protein of 55 kDa OS=Homo sapiens GN=CEP55 PE=1 SV=3 | sp\|Q53EZ4\|CEP55_HUMAN | 54 kDa |
| 247 | T-complex protein 1 subunit eta OS=Homo sapiens GN=CCT7 PE=1 SV=2 | sp\|Q99832\|TCPH_HUMAN | 59 kDa |
| 248 | tRNA (adenine(58)-N(1))-methyltransferase non-catalytic subunit TRM6 OS=Homo sapiens GN=TRMT6 PE=1 SV=1 | sp\|Q9UJA5\|TRM6_HUMAN | 56 kDa |
| 249 | Integrin-linked protein kinase OS=Homo sapiens GN=ILK PE=1 SV=2 | sp\|Q13418\|ILK_HUMAN (+2) | 51 kDa |
| 250 | Enhancer of mRNA-decapping protein 3 OS=Homo sapiens GN=EDC3 PE=1 SV=1 | sp\|Q96F86\|EDC3_HUMAN | 56 kDa |
| 251 | ERO1-like protein alpha OS=Homo sapiens GN=ERO1A PE=1 SV=2 | sp\|Q96HE7\|ERO1A_HUMAN | 54 kDa |
| 252 | RNA binding protein fox-1 homolog 2 OS=Homo sapiens GN=RBFOX2 PE=1 SV=3 | sp\|O43251\|RFOX2_HUMAN (+1) | 41 kDa |
| 253 | Stress-induced-phosphoprotein 1 OS=Homo sapiens GN=STIP1 PE=1 SV=1 | sp\|P31948\|STIP1_HUMAN (+2) | 63 kDa |
| 254 | UV excision repair protein RAD23 homolog B OS=Homo sapiens GN=RAD23B PE=1 SV=1 | sp\|P54727\|RD23B_HUMAN (+2) | 43 kDa |
| 255 | Cytoskeleton-associated protein 4 OS=Homo sapiens GN=CKAP4 PE=1 SV=2 | sp\|Q07065\|CKAP4_HUMAN | 66 kDa |
| 256 | Transcriptional adapter 2-beta OS=Homo sapiens GN=TADA2B PE=1 SV=2 | sp\|Q86TJ2\|TAD2B_HUMAN | 48 kDa |
| 257 | RNA-binding protein 14 OS=Homo sapiens GN=RBM14 PE=1 SV=2 | sp\|Q96PK6\|RBM14_HUMAN (+1) | 69 kDa |
| 258 | Protein HGH1 homolog OS=Homo sapiens GN=HGH1 PE=1 SV=1 | sp\|Q9BTY7\|HGH1_HUMAN | 42 kDa |
| 259 | Ubiquitin-like modifier-activating enzyme 5 OS=Homo sapiens GN=UBA5 PE=1 SV=1 | sp\|Q9GZZ9\|UBA5_HUMAN | 45 kDa |
| 260 | Zinc finger protein 277 OS=Homo sapiens GN=ZNF277 PE=1 SV=2 | sp\|Q9NRM2\|ZN277_HUMAN (+1) | 53 kDa |
| 261 | cDNA FLJ55936, highly similar to Polypyrimidine tract-binding protein 2 OS=Homo sapiens PE=2 SV=1 | tr\|B4DSS8\|B4DSS8_HUMAN | 60 kDa |
| 262 | Serpin H1 OS=Homo sapiens GN=SERPINH1 PE=1 SV=2 | sp\|P50454\|SERPH_HUMAN (+1) | 46 kDa |
| 263 | Peptidyl-prolyl cis-trans isomerase FKBP5 OS=Homo sapiens GN=FKBP5 PE=1 SV=2 | sp\|Q13451\|FKBP5_HUMAN (+2) | 51 kDa |
| 264 | Protein PRRC1 OS=Homo sapiens GN=PRRC1 PE=1 SV=1 | sp\|Q96M27\|PRRC1_HUMAN | 47 kDa |
| 265 | Drebrin-like protein OS=Homo sapiens GN=DBNL PE=1 SV=1 | sp\|Q9UJU6\|DBNL_HUMAN | 48 kDa |
| 266 | Zinc finger protein 787 OS=Homo sapiens GN=ZNF787 PE=1 SV=1 | tr\|A0A087WUD1\|A0A087WUD1_HUMAN | 40 kDa |
| 267 | DnaJ homolog subfamily A member 1 OS=Homo sapiens GN=DNAJA1 PE=1 SV=2 | sp\|P31689\|DNJA1_HUMAN | 45 kDa |
| 268 | Secernin-1 OS=Homo sapiens GN=SCRN1 PE=1 SV=2 | sp\|Q12765\|SCRN1_HUMAN (+1) | 46 kDa |
| 269 | Exportin-1 OS=Homo sapiens GN=XPO1 PE=1 SV=1 | sp\|O14980\|XPO1_HUMAN (+3) | 123 kDa |
| 270 | DNA polymerase delta subunit 2 OS=Homo sapiens GN=POLD2 PE=1 SV=1 | sp\|P49005\|DPOD2_HUMAN (+1) | 51 kDa |
| 271 | Cleavage and polyadenylation specificity factor subunit 6 OS=Homo sapiens GN=CPSF6 PE=1 SV=2 | sp\|Q16630\|CPSF6_HUMAN (+1) | 59 kDa |
| 272 | Endophilin-B2 OS=Homo sapiens GN=SH3GLB2 PE=1 SV=1 | sp\|Q9NR46\|SHLB2_HUMAN (+2) | 44 kDa |
| 273 | Zinc finger and SCAN domain-containing protein 21 OS=Homo sapiens GN=ZSCAN21 PE=1 SV=2 | sp\|Q9Y5A6\|ZSC21_HUMAN (+1) | 54 kDa |
| 274 | cDNA FLJ77283, highly similar to Homo sapiens G patch domain containing 2, mRNA OS=Homo sapiens PE=2 SV=1 | tr\|A8K5C3\|A8K5C3_HUMAN | 43 kDa |
| 275 | Serine/threonine-protein kinase VRK1 OS=Homo sapiens GN=VRK1 PE=1 SV=1 | sp\|Q99986\|VRK1_HUMAN (+1) | 45 kDa |
| 276 | Glomulin OS=Homo sapiens GN=GLMN PE=1 SV=2 | sp\|Q92990\|GLMN_HUMAN | 68 kDa |
| 277 | Pyruvate dehydrogenase protein X component, mitochondrial OS=Homo sapiens GN=PDHX PE=1 SV=3 | sp\|O00330\|ODPX_HUMAN (+1) | 54 kDa |
| 278 | Mitochondrial ribonuclease P protein 3 OS=Homo sapiens GN=KIAA0391 PE=1 SV=2 | sp\|O15091\|MRRP3_HUMAN | 67 kDa |
| 279 | Vacuolar protein sorting-associated protein 4B OS=Homo sapiens GN=VPS4B PE=1 SV=2 | sp\|O75351\|VPS4B_HUMAN | 49 kDa |
| 280 | Zinc finger protein RFP OS=Homo sapiens GN=TRIM27 PE=1 SV=1 | sp\|P14373\|TRI27_HUMAN (+1) | 58 kDa |
| 281 | Protein ERGIC-53 OS=Homo sapiens GN=LMAN1 PE=1 SV=2 | sp\|P49257\|LMAN1_HUMAN (+2) | 58 kDa |
| 282 | LEM domain-containing protein 2 OS=Homo sapiens GN=LEMD2 PE=1 SV=1 | sp\|Q8NC56\|LEMD2_HUMAN (+1) | 57 kDa |
| 283 | Zinc finger protein with KRAB and SCAN domains 4 OS=Homo sapiens GN=ZKSCAN4 PE=1 SV=1 | sp\|Q969J2\|ZKSC4_HUMAN (+1) | 62 kDa |
| 284 | Leukocyte receptor cluster member 9 OS=Homo sapiens GN=LENG9 PE=1 SV=1 | tr\|A0A087WVD1\|A0A087WVD1_HUMAN | 51 kDa |
| 285 | RNA-binding protein with serine-rich domain 1 OS=Homo sapiens GN=RNPS1 PE=1 SV=1 | sp\|Q15287\|RNPS1_HUMAN (+4) | 34 kDa |
| 286 | 3'-5' exoribonuclease 1 OS=Homo sapiens GN=ERI1 PE=1 SV=3 | sp\|Q8IV48\|ERI1_HUMAN (+1) | 40 kDa |
| 287 | Heat shock 70 kDa protein 14 OS=Homo sapiens GN=HSPA14 PE=1 SV=1 | sp\|Q0VDF9\|HSP7E_HUMAN | 55 kDa |
| 288 | Methylcrotonoyl-CoA carboxylase beta chain, mitochondrial OS=Homo sapiens GN=MCCC2 PE=1 SV=1 | sp\|Q9HCC0\|MCCB_HUMAN (+1) | 61 kDa |
| 289 | DNA methyltransferase 1-associated protein 1 OS=Homo sapiens GN=DMAP1 PE=1 SV=1 | sp\|Q9NPF5\|DMAP1_HUMAN (+1) | 53 kDa |
| 290 | Eukaryotic translation initiation factor 3 subunit G OS=Homo sapiens GN=EIF3G PE=1 SV=2 | sp\|O75821\|EIF3G_HUMAN (+1) | 36 kDa |
| 291 | BUB3-interacting and GLEBS motif-containing protein ZNF207 OS=Homo sapiens GN=ZNF207 PE=1 SV=1 | sp\|O43670\|ZN207_HUMAN (+3) | 51 kDa |
| 292 | CD2 antigen cytoplasmic tail-binding protein 2 OS=Homo sapiens GN=CD2BP2 PE=1 SV=1 | sp\|O95400\|CD2B2_HUMAN (+1) | 38 kDa |
| 293 | 78 kDa glucose-regulated protein OS=Homo sapiens GN=HSPA5 PE=1 SV=2 | sp\|P11021\|GRP78_HUMAN (+1) | 72 kDa |
| 294 | 40S ribosomal protein S3a OS=Homo sapiens GN=RPS3A PE=1 SV=2 | sp\|P61247\|RS3A_HUMAN (+1) | 30 kDa |
| 295 | Peptidyl-prolyl cis-trans isomerase FKBP8 OS=Homo sapiens GN=FKBP8 PE=1 SV=2 | sp\|Q14318\|FKBP8_HUMAN (+3) | 45 kDa |
| 296 | Lysophospholipid acyltransferase LPCAT4 OS=Homo sapiens GN=LPCAT4 PE=1 SV=1 | sp\|Q643R3\|LPCT4_HUMAN | 57 kDa |
| 297 | Putative ATP-dependent RNA helicase DHX30 OS=Homo sapiens GN=DHX30 PE=1 SV=1 | sp\|Q7L2E3\|DHX30_HUMAN (+2) | 134 kDa |
| 298 | Translocation protein SEC62 OS=Homo sapiens GN=SEC62 PE=1 SV=1 | sp\|Q99442\|SEC62_HUMAN | 46 kDa |
| 299 | Coiled-coil domain-containing protein 77 OS=Homo sapiens GN=CCDC77 PE=2 SV=1 | sp\|Q9BR77\|CCD77_HUMAN | 57 kDa |
| 300 | 60S acidic ribosomal protein P0 OS=Homo sapiens GN=RPLP0 PE=1 SV=1 | sp\|P05388\|RLA0_HUMAN (+2) | 34 kDa |

| # | Identified Proteins (716) | Accession Number | Molecular  weight |
| --- | --- | --- | --- |
| 301 | Probable RNA-binding protein 23 OS=Homo sapiens GN=RBM23 PE=1 SV=1 | sp\|Q86U06\|RBM23_HUMAN (+2) | 49 kDa |
| 302 | RNA-binding protein 45 OS=Homo sapiens GN=RBM45 PE=1 SV=1 | sp\|Q8IUH3\|RBM45_HUMAN | 54 kDa |
| 303 | Serine/arginine-rich splicing factor 11 OS=Homo sapiens GN=SRSF11 PE=1 SV=1 | sp\|Q05519\|SRS11_HUMAN | 54 kDa |
| 304 | Cell division cycle protein 123 homolog OS=Homo sapiens GN=CDC123 PE=1 SV=1 | sp\|O75794\|CD123_HUMAN | 39 kDa |
| 305 | Fumarate hydratase, mitochondrial OS=Homo sapiens GN=FH PE=1 SV=3 | sp\|P07954\|FUMH_HUMAN (+1) | 55 kDa |
| 306 | Pre-mRNA 3'-end-processing factor FIP1 OS=Homo sapiens GN=FIP1L1 PE=1 SV=1 | sp\|Q6UN15\|FIP1_HUMAN | 67 kDa |
| 307 | Nucleolar protein 58 OS=Homo sapiens GN=NOP58 PE=1 SV=1 | sp\|Q9Y2X3\|NOP58_HUMAN | 60 kDa |
| 308 | cDNA FLJ60942, highly similar to Phosphatidylinositol 3-kinase regulatory subunit gamma OS=Homo sapiens PE=2 SV=1 | tr\|B4DXM8\|B4DXM8_HUMAN (+1) | 59 kDa |
| 309 | Ribosomal RNA processing protein 1 homolog B OS=Homo sapiens GN=RRP1B PE=1 SV=3 | sp\|Q14684\|RRP1B_HUMAN | 84 kDa |
| 310 | Atypical kinase COQ8B, mitochondrial OS=Homo sapiens GN=COQ8B PE=1 SV=2 | sp\|Q96D53\|COQ8B_HUMAN | 60 kDa |
| 311 | KH domain-containing, RNA-binding, signal transduction-associated protein 3 OS=Homo sapiens GN=KHDRBS3 PE=1 SV=1 | sp\|O75525\|KHDR3_HUMAN | 39 kDa |
| 312 | 5'-nucleotidase domain-containing protein 1 OS=Homo sapiens GN=NT5DC1 PE=1 SV=1 | sp\|Q5TFE4\|NT5D1_HUMAN | 52 kDa |
| 313 | UDP-N-acetylglucosamine--peptide N-acetylglucosaminyltransferase 110 kDa subunit OS=Homo sapiens GN=OGT PE=1 SV=3 | sp\|O15294\|OGT1_HUMAN | 117 kDa |
| 314 | Inosine-5'-monophosphate dehydrogenase 1 OS=Homo sapiens GN=IMPDH1 PE=1 SV=2 | sp\|P20839\|IMDH1_HUMAN (+4) | 55 kDa |
| 315 | Telomeric repeat-binding factor 1 OS=Homo sapiens GN=TERF1 PE=1 SV=3 | sp\|P54274\|TERF1_HUMAN (+2) | 50 kDa |
| 316 | Hydroxymethylglutaryl-CoA synthase, cytoplasmic OS=Homo sapiens GN=HMGCS1 PE=1 SV=2 | sp\|Q01581\|HMCS1_HUMAN (+2) | 57 kDa |
| 317 | Probable ATP-dependent RNA helicase DDX28 OS=Homo sapiens GN=DDX28 PE=1 SV=2 | sp\|Q9NUL7\|DDX28_HUMAN | 60 kDa |
| 318 | Zinc finger HIT domain-containing protein 2 OS=Homo sapiens GN=ZNHIT2 PE=1 SV=1 | sp\|Q9UHR6\|ZNHI2_HUMAN (+2) | 43 kDa |
| 319 | Heterogeneous nuclear ribonucleoprotein R OS=Homo sapiens GN=HNRNPR PE=1 SV=1 | sp\|O43390\|HNRPR_HUMAN (+1) | 71 kDa |
| 320 | Target of Myb protein 1 OS=Homo sapiens GN=TOM1 PE=1 SV=2 | sp\|O60784\|TOM1_HUMAN (+2) | 54 kDa |
| 321 | 6-phosphogluconate dehydrogenase, decarboxylating OS=Homo sapiens GN=PGD PE=1 SV=3 | sp\|P52209\|6PGD_HUMAN | 53 kDa |
| 322 | Caveolae-associated protein 1 OS=Homo sapiens GN=CAVIN1 PE=1 SV=1 | sp\|Q6NZI2\|CAVN1_HUMAN (+2) | 43 kDa |
| 323 | Paraneoplastic antigen-like protein 8A OS=Homo sapiens GN=PNMA8A PE=1 SV=2 | sp\|Q86V59\|PNM8A_HUMAN | 48 kDa |
| 324 | BAG family molecular chaperone regulator 1 OS=Homo sapiens GN=BAG1 PE=1 SV=4 | sp\|Q99933\|BAG1_HUMAN (+2) | 39 kDa |
| 325 | Splicing factor 3A subunit 2 OS=Homo sapiens GN=SF3A2 PE=1 SV=2 | sp\|Q15428\|SF3A2_HUMAN (+1) | 49 kDa |
| 326 | Tubulin alpha-1A chain OS=Homo sapiens GN=TUBA1A PE=1 SV=1 | sp\|Q71U36\|TBA1A_HUMAN | 50 kDa |
| 327 | Ribosomal RNA-processing protein 8 OS=Homo sapiens GN=RRP8 PE=1 SV=2 | sp\|O43159\|RRP8_HUMAN | 51 kDa |
| 328 | 55 kDa erythrocyte membrane protein OS=Homo sapiens GN=MPP1 PE=1 SV=2 | sp\|Q00013\|EM55_HUMAN (+2) | 52 kDa |
| 329 | ATP-dependent Clp protease ATP-binding subunit clpX-like, mitochondrial OS=Homo sapiens GN=CLPX PE=1 SV=2 | sp\|O76031\|CLPX_HUMAN (+1) | 69 kDa |
| 330 | Uridine 5'-monophosphate synthase OS=Homo sapiens GN=UMPS PE=1 SV=1 | sp\|P11172\|UMPS_HUMAN (+2) | 52 kDa |
| 331 | Caspase activity and apoptosis inhibitor 1 OS=Homo sapiens GN=CAAP1 PE=1 SV=2 | sp\|Q9H8G2\|CAAP1_HUMAN | 38 kDa |
| 332 | Probable cysteine--tRNA ligase, mitochondrial OS=Homo sapiens GN=CARS2 PE=1 SV=1 | sp\|Q9HA77\|SYCM_HUMAN | 62 kDa |
| 333 | DNA-directed RNA polymerase III subunit RPC4 OS=Homo sapiens GN=POLR3D PE=1 SV=2 | sp\|P05423\|RPC4_HUMAN | 44 kDa |
| 334 | DNA-directed RNA polymerase III subunit RPC3 OS=Homo sapiens GN=POLR3C PE=1 SV=1 | sp\|Q9BUI4\|RPC3_HUMAN (+2) | 61 kDa |
| 335 | cDNA FLJ78127, highly similar to Homo sapiens SFRS protein kinase 1 (SRPK1), mRNA OS=Homo sapiens PE=2 SV=1 | tr\|A8K8B2\|A8K8B2_HUMAN (+1) | 74 kDa |
| 336 | Keratin, type I cytoskeletal 9 OS=Homo sapiens GN=KRT9 PE=1 SV=3 | sp\|P35527\|K1C9_HUMAN | 62 kDa |
| 337 | Paraspeckle component 1 OS=Homo sapiens GN=PSPC1 PE=1 SV=1 | sp\|Q8WXF1\|PSPC1_HUMAN (+2) | 59 kDa |
| 338 | Glucosylceramidase OS=Homo sapiens GN=GBA PE=1 SV=3 | sp\|P04062\|GLCM_HUMAN (+6) | 60 kDa |
| 339 | Keratin, type II cytoskeletal 2 epidermal OS=Homo sapiens GN=KRT2 PE=1 SV=2 | sp\|P35908\|K22E_HUMAN | 65 kDa |
| 340 | SWI/SNF-related matrix-associated actin-dependent regulator of chromatin subfamily D member 2 OS=Homo sapiens GN=SMARCD2 PE=1 SV=3 | sp\|Q92925\|SMRD2_HUMAN (+1) | 59 kDa |
| 341 | Inositol-3-phosphate synthase 1 OS=Homo sapiens GN=ISYNA1 PE=1 SV=1 | sp\|Q9NPH2\|INO1_HUMAN (+2) | 61 kDa |
| 342 | RNA binding motif single stranded interacting protein 1 isoform 2 (Fragment) OS=Homo sapiens GN=RBMS1 PE=2 SV=1 | tr\|A0A0S2Z4B3\|A0A0S2Z4B3_HUMAN | 46 kDa |
| 343 | Creatine kinase U-type, mitochondrial OS=Homo sapiens GN=CKMT1A PE=1 SV=1 | sp\|P12532\|KCRU_HUMAN | 47 kDa |
| 344 | Peptidyl-prolyl cis-trans isomerase CWC27 homolog OS=Homo sapiens GN=CWC27 PE=1 SV=1 | sp\|Q6UX04\|CWC27_HUMAN (+1) | 54 kDa |
| 345 | Pre-mRNA-splicing factor RBM22 OS=Homo sapiens GN=RBM22 PE=1 SV=1 | sp\|Q9NW64\|RBM22_HUMAN | 47 kDa |
| 346 | Ubiquitin-like modifier-activating enzyme 1 OS=Homo sapiens GN=UBA1 PE=1 SV=3 | sp\|P22314\|UBA1_HUMAN (+1) | 118 kDa |
| 347 | BRCA2 and CDKN1A-interacting protein OS=Homo sapiens GN=BCCIP PE=1 SV=1 | sp\|Q9P287\|BCCIP_HUMAN (+2) | 36 kDa |
| 348 | Phosphoglycerate kinase 1 OS=Homo sapiens GN=PGK1 PE=1 SV=3 | sp\|P00558\|PGK1_HUMAN (+2) | 45 kDa |
| 349 | Methylmalonate-semialdehyde dehydrogenase [acylating], mitochondrial OS=Homo sapiens GN=ALDH6A1 PE=1 SV=2 | sp\|Q02252\|MMSA_HUMAN (+1) | 58 kDa |
| 350 | Death-associated protein kinase 3 OS=Homo sapiens GN=DAPK3 PE=1 SV=1 | sp\|O43293\|DAPK3_HUMAN (+1) | 53 kDa |
| 351 | Importin-5 OS=Homo sapiens GN=IPO5 PE=1 SV=4 | sp\|O00410\|IPO5_HUMAN (+2) | 124 kDa |
| 352 | SNW domain-containing protein 1 OS=Homo sapiens GN=SNW1 PE=1 SV=1 | sp\|Q13573\|SNW1_HUMAN (+3) | 61 kDa |
| 353 | cDNA FLJ76669, highly similar to Homo sapiens zinc finger protein 278 (ZNF278), transcript variant 4, mRNA OS=Homo sapiens PE=2 SV=1 | tr\|A8K6Y8\|A8K6Y8_HUMAN | 58 kDa |
| 354 | Zinc finger protein with KRAB and SCAN domains 8 OS=Homo sapiens GN=ZKSCAN8 PE=1 SV=2 | sp\|Q15776\|ZKSC8_HUMAN (+1) | 66 kDa |
| 355 | Tumor susceptibility gene 101 protein OS=Homo sapiens GN=TSG101 PE=1 SV=2 | sp\|Q99816\|TS101_HUMAN | 44 kDa |
| 356 | Polyadenylate-binding protein OS=Homo sapiens GN=PABPC4 PE=1 SV=1 | tr\|B1ANR0\|B1ANR0_HUMAN | 68 kDa |
| 357 | Retinal dehydrogenase 2 OS=Homo sapiens GN=ALDH1A2 PE=1 SV=3 | sp\|O94788\|AL1A2_HUMAN | 57 kDa |
| 358 | Splicing factor 1 OS=Homo sapiens GN=SF1 PE=1 SV=4 | sp\|Q15637\|SF01_HUMAN (+5) | 68 kDa |
| 359 | Interferon-related developmental regulator 2 OS=Homo sapiens GN=IFRD2 PE=1 SV=1 | tr\|A0A0R4J2F6\|A0A0R4J2F6_HUMAN (+3) | 48 kDa |
| 360 | Protein SDE2 homolog OS=Homo sapiens GN=SDE2 PE=1 SV=1 | sp\|Q6IQ49\|SDE2_HUMAN | 50 kDa |

| # | Identified Proteins (716) | Accession Number | Molecular  weight |
| --- | --- | --- | --- |
| 361 | ADP-dependent glucokinase OS=Homo sapiens GN=ADPGK PE=1 SV=1 | sp\|Q9BRR6\|ADPGK_HUMAN | 54 kDa |
| 362 | 60S ribosomal protein L6 OS=Homo sapiens GN=RPL6 PE=1 SV=3 | sp\|Q02878\|RL6_HUMAN (+4) | 33 kDa |
| 363 | DNA-directed RNA polymerase I subunit RPA49 OS=Homo sapiens GN=POLR1E PE=1 SV=2 | sp\|Q9GZS1\|RPA49_HUMAN | 54 kDa |
| 364 | 26S proteasome regulatory subunit 7 OS=Homo sapiens GN=PSMC2 PE=1 SV=3 | sp\|P35998\|PRS7_HUMAN (+3) | 49 kDa |
| 365 | Flap endonuclease 1 OS=Homo sapiens GN=FEN1 PE=1 SV=1 | sp\|P39748\|FEN1_HUMAN | 43 kDa |
| 366 | Golgi reassembly-stacking protein 2 OS=Homo sapiens GN=GORASP2 PE=1 SV=3 | sp\|Q9H8Y8\|GORS2_HUMAN (+1) | 47 kDa |
| 367 | NEDD8-activating enzyme E1 regulatory subunit OS=Homo sapiens GN=NAE1 PE=1 SV=1 | sp\|Q13564\|ULA1_HUMAN | 60 kDa |
| 368 | Corepressor interacting with RBPJ 1 OS=Homo sapiens GN=CIR1 PE=1 SV=1 | sp\|Q86X95\|CIR1_HUMAN | 52 kDa |
| 369 | Creatine kinase B-type OS=Homo sapiens GN=CKB PE=1 SV=1 | sp\|P12277\|KCRB_HUMAN (+3) | 43 kDa |
| 370 | Beta-1,4-glucuronyltransferase 1 OS=Homo sapiens GN=B4GAT1 PE=1 SV=1 | sp\|O43505\|B4GA1_HUMAN (+1) | 47 kDa |
| 371 | General transcription factor 3C polypeptide 5 OS=Homo sapiens GN=GTF3C5 PE=1 SV=2 | sp\|Q9Y5Q8\|TF3C5_HUMAN (+1) | 60 kDa |
| 372 | Tetratricopeptide repeat protein 4 OS=Homo sapiens GN=TTC4 PE=1 SV=3 | sp\|O95801\|TTC4_HUMAN | 45 kDa |
| 373 | Serine/threonine-protein phosphatase 2A 56 kDa regulatory subunit gamma isoform OS=Homo sapiens GN=PPP2R5C PE=1 SV=3 | sp\|Q13362\|2A5G_HUMAN (+1) | 61 kDa |
| 374 | Eukaryotic peptide chain release factor subunit 1 OS=Homo sapiens GN=ETF1 PE=1 SV=3 | sp\|P62495\|ERF1_HUMAN (+1) | 49 kDa |
| 375 | Synapse-associated protein 1 OS=Homo sapiens GN=SYAP1 PE=1 SV=1 | sp\|Q96A49\|SYAP1_HUMAN (+1) | 40 kDa |
| 376 | Protein misato homolog 1 OS=Homo sapiens GN=MSTO1 PE=1 SV=1 | sp\|Q9BUK6\|MSTO1_HUMAN | 62 kDa |
| 377 | Cytoplasmic tRNA 2-thiolation protein 2 OS=Homo sapiens GN=CTU2 PE=1 SV=1 | sp\|Q2VPK5\|CTU2_HUMAN (+1) | 56 kDa |
| 378 | cDNA FLJ10358 fis, clone NT2RM2001238, highly similar to Glutaminase kidney isoform, mitochondrial (Fragment) OS=Homo sapiens PE=2 SV=1 | tr\|B3KM58\|B3KM58_HUMAN | 41 kDa |
| 379 | Transmembrane and coiled-coil domain protein 3 OS=Homo sapiens GN=TMCC3 PE=2 SV=3 | sp\|Q9ULS5\|TMCC3_HUMAN (+1) | 54 kDa |
| 380 | Nuclear migration protein nudC OS=Homo sapiens GN=NUDC PE=1 SV=1 | sp\|Q9Y266\|NUDC_HUMAN | 38 kDa |
| 381 | FGFR1 oncogene partner OS=Homo sapiens GN=FGFR1OP PE=1 SV=1 | sp\|O95684\|FR1OP_HUMAN (+2) | 43 kDa |
| 382 | Rab GDP dissociation inhibitor alpha OS=Homo sapiens GN=GDI1 PE=1 SV=2 | sp\|P31150\|GDIA_HUMAN (+1) | 51 kDa |
| 383 | Polycomb group RING finger protein 6 OS=Homo sapiens GN=PCGF6 PE=1 SV=2 | sp\|Q9BYE7\|PCGF6_HUMAN | 39 kDa |
| 384 | 60S ribosomal protein L7 OS=Homo sapiens GN=RPL7 PE=1 SV=1 | sp\|P18124\|RL7_HUMAN (+1) | 29 kDa |
| 385 | Cartilage-associated protein OS=Homo sapiens GN=CRTAP PE=1 SV=1 | sp\|O75718\|CRTAP_HUMAN (+5) | 47 kDa |
| 386 | DAZ-associated protein 1 OS=Homo sapiens GN=DAZAP1 PE=1 SV=1 | sp\|Q96EP5\|DAZP1_HUMAN (+3) | 43 kDa |
| 387 | Alanine aminotransferase 2 OS=Homo sapiens GN=GPT2 PE=1 SV=1 | sp\|Q8TD30\|ALAT2_HUMAN (+1) | 58 kDa |
| 388 | TGF-beta-activated kinase 1 and MAP3K7-binding protein 1 OS=Homo sapiens GN=TAB1 PE=1 SV=1 | sp\|Q15750\|TAB1_HUMAN (+2) | 55 kDa |
| 389 | Protein LYRIC OS=Homo sapiens GN=MTDH PE=1 SV=2 | sp\|Q86UE4\|LYRIC_HUMAN (+1) | 64 kDa |
| 390 | Myelin expression factor 2 OS=Homo sapiens GN=MYEF2 PE=1 SV=3 | sp\|Q9P2K5\|MYEF2_HUMAN (+3) | 64 kDa |
| 391 | Probable ATP-dependent RNA helicase DDX56 OS=Homo sapiens GN=DDX56 PE=1 SV=1 | sp\|Q9NY93\|DDX56_HUMAN (+1) | 62 kDa |
| 392 | KDEL motif-containing protein 2 OS=Homo sapiens GN=KDELC2 PE=1 SV=2 | sp\|Q7Z4H8\|KDEL2_HUMAN (+1) | 59 kDa |
| 393 | Heterogeneous nuclear ribonucleoproteins C1/C2 OS=Homo sapiens GN=HNRNPC PE=1 SV=4 | sp\|P07910\|HNRPC_HUMAN (+4) | 34 kDa |
| 394 | Tubulin gamma-1 chain OS=Homo sapiens GN=TUBG1 PE=1 SV=2 | sp\|P23258\|TBG1_HUMAN (+2) | 51 kDa |
| 395 | Periodic tryptophan protein 1 homolog OS=Homo sapiens GN=PWP1 PE=1 SV=1 | sp\|Q13610\|PWP1_HUMAN (+4) | 56 kDa |
| 396 | Negative elongation factor C/D OS=Homo sapiens GN=NELFCD PE=1 SV=2 | sp\|Q8IXH7\|NELFD_HUMAN (+2) | 66 kDa |
| 397 | Kinetochore protein Nuf2 OS=Homo sapiens GN=NUF2 PE=1 SV=2 | sp\|Q9BZD4\|NUF2_HUMAN (+1) | 54 kDa |
| 398 | Polycomb group RING finger protein 2 OS=Homo sapiens GN=PCGF2 PE=1 SV=1 | sp\|P35227\|PCGF2_HUMAN (+3) | 38 kDa |
| 399 | Phosphorylated adapter RNA export protein OS=Homo sapiens GN=PHAX PE=1 SV=1 | sp\|Q9H814\|PHAX_HUMAN | 44 kDa |
| 400 | Glyceraldehyde-3-phosphate dehydrogenase OS=Homo sapiens GN=GAPDH PE=1 SV=3 | sp\|P04406\|G3P_HUMAN (+2) | 36 kDa |
| 401 | F-box-like/WD repeat-containing protein TBL1XR1 OS=Homo sapiens GN=TBL1XR1 PE=1 SV=1 | sp\|Q9BZK7\|TBL1R_HUMAN (+1) | 56 kDa |
| 402 | Zinc finger protein 460 OS=Homo sapiens GN=ZNF460 PE=1 SV=2 | sp\|Q14592\|ZN460_HUMAN (+1) | 64 kDa |
| 403 | Glutathione S-transferase P OS=Homo sapiens GN=GSTP1 PE=1 SV=2 | sp\|P09211\|GSTP1_HUMAN (+1) | 23 kDa |
| 404 | IST1 homolog (Fragment) OS=Homo sapiens GN=IST1 PE=1 SV=1 | tr\|H3BUI0\|H3BUI0_HUMAN | 28 kDa |
| 405 | Transcriptional adapter 3 OS=Homo sapiens GN=TADA3 PE=1 SV=1 | sp\|O75528\|TADA3_HUMAN (+2) | 49 kDa |
| 406 | Serine/threonine-protein kinase 24 OS=Homo sapiens GN=STK24 PE=1 SV=1 | sp\|Q9Y6E0\|STK24_HUMAN (+2) | 49 kDa |
| 407 | Zinc finger CCCH-type antiviral protein 1 OS=Homo sapiens GN=ZC3HAV1 PE=1 SV=3 | sp\|Q7Z2W4\|ZCCHV_HUMAN (+2) | 101 kDa |
| 408 | Nuclear autoantigenic sperm protein OS=Homo sapiens GN=NASP PE=1 SV=2 | sp\|P49321\|NASP_HUMAN (+2) | 85 kDa |
| 409 | Vascular endothelial zinc finger 1 OS=Homo sapiens GN=VEZF1 PE=1 SV=2 | sp\|Q14119\|VEZF1_HUMAN (+1) | 57 kDa |
| 410 | E3 ubiquitin-protein ligase ARIH2 OS=Homo sapiens GN=ARIH2 PE=1 SV=1 | sp\|O95376\|ARI2_HUMAN (+3) | 58 kDa |
| 411 | Heat shock 70 kDa protein 13 OS=Homo sapiens GN=HSPA13 PE=1 SV=1 | sp\|P48723\|HSP13_HUMAN (+3) | 52 kDa |
| 412 | Spermatogenesis-associated serine-rich protein 2 OS=Homo sapiens GN=SPATS2 PE=1 SV=1 | sp\|Q86XZ4\|SPAS2_HUMAN (+1) | 60 kDa |
| 413 | Heat shock protein HSP 90-beta OS=Homo sapiens GN=HSP90AB1 PE=1 SV=4 | sp\|P08238\|HS90B_HUMAN (+2) | 83 kDa |
| 414 | Myc-associated zinc finger protein OS=Homo sapiens GN=MAZ PE=1 SV=1 | sp\|P56270\|MAZ_HUMAN (+1) | 49 kDa |
| 415 | V-type proton ATPase subunit S1 OS=Homo sapiens GN=ATP6AP1 PE=1 SV=2 | sp\|Q15904\|VAS1_HUMAN (+2) | 52 kDa |
| 416 | Eukaryotic translation initiation factor 3 subunit D OS=Homo sapiens GN=EIF3D PE=1 SV=1 | sp\|O15371\|EIF3D_HUMAN (+1) | 64 kDa |
| 417 | Putative E3 ubiquitin-protein ligase UBR7 OS=Homo sapiens GN=UBR7 PE=1 SV=2 | sp\|Q8N806\|UBR7_HUMAN | 48 kDa |
| 418 | Reticulocalbin-2 OS=Homo sapiens GN=RCN2 PE=1 SV=1 | sp\|Q14257\|RCN2_HUMAN | 37 kDa |
| 419 | cDNA FLJ54552, highly similar to Heterogeneous nuclear ribonucleoprotein K OS=Homo sapiens PE=2 SV=1 | tr\|B4DUQ1\|B4DUQ1_HUMAN | 49 kDa |
| 420 | Coilin OS=Homo sapiens GN=COIL PE=1 SV=1 | sp\|P38432\|COIL_HUMAN | 63 kDa |

| # | Identified Proteins (716) | Accession Number | Molecular  weight |
| --- | --- | --- | --- |
| 421 | Transcription intermediary factor 1-beta OS=Homo sapiens GN=TRIM28 PE=1 SV=5 | sp\|Q13263\|TIF1B_HUMAN (+1) | 89 kDa |
| 422 | 40S ribosomal protein S4, X isoform OS=Homo sapiens GN=RPS4X PE=1 SV=2 | sp\|P62701\|RS4X_HUMAN (+3) | 30 kDa |
| 423 | NudC-like protein OS=Homo sapiens PE=1 SV=1 | tr\|B0FTY2\|B0FTY2_HUMAN | 41 kDa |
| 424 | Glutamyl-tRNA(Gln) amidotransferase subunit A, mitochondrial OS=Homo sapiens GN=QRSL1 PE=1 SV=2 | sp\|Q9H0R6\|GATA_HUMAN | 57 kDa |
| 425 | Serine/threonine-protein kinase OSR1 OS=Homo sapiens GN=OXSR1 PE=1 SV=1 | sp\|O95747\|OXSR1_HUMAN (+1) | 58 kDa |
| 426 | Serine/threonine-protein phosphatase 2B catalytic subunit alpha isoform OS=Homo sapiens GN=PPP3CA PE=1 SV=1 | sp\|Q08209\|PP2BA_HUMAN (+2) | 59 kDa |
| 427 | Bifunctional polynucleotide phosphatase/kinase OS=Homo sapiens GN=PNKP PE=1 SV=1 | sp\|Q96T60\|PNKP_HUMAN (+3) | 57 kDa |
| 428 | 60S ribosomal protein L9 OS=Homo sapiens GN=RPL9 PE=1 SV=1 | sp\|P32969\|RL9_HUMAN (+2) | 22 kDa |
| 429 | Pleckstrin homology domain-containing family O member 1 OS=Homo sapiens GN=PLEKHO1 PE=1 SV=2 | sp\|Q53GL0\|PKHO1_HUMAN (+1) | 46 kDa |
| 430 | Actin-like protein 6A OS=Homo sapiens GN=ACTL6A PE=1 SV=1 | sp\|O96019\|ACL6A_HUMAN (+1) | 47 kDa |
| 431 | HAUS augmin-like complex subunit 8 OS=Homo sapiens GN=HAUS8 PE=1 SV=3 | sp\|Q9BT25\|HAUS8_HUMAN | 45 kDa |
| 432 | Adipocyte plasma membrane-associated protein OS=Homo sapiens GN=APMAP PE=1 SV=2 | sp\|Q9HDC9\|APMAP_HUMAN | 46 kDa |
| 433 | Cystathionine beta-synthase-like protein OS=Homo sapiens GN=CBSL PE=1 SV=1 | sp\|P0DN79\|CBSL_HUMAN (+2) | 61 kDa |
| 434 | Arginine--tRNA ligase, cytoplasmic OS=Homo sapiens GN=RARS PE=1 SV=2 | sp\|P54136\|SYRC_HUMAN | 75 kDa |
| 435 | Actin, alpha cardiac muscle 1 OS=Homo sapiens GN=ACTC1 PE=1 SV=1 | sp\|P68032\|ACTC_HUMAN (+1) | 42 kDa |
| 436 | Host cell factor 1 OS=Homo sapiens GN=HCFC1 PE=1 SV=2 | sp\|P51610\|HCFC1_HUMAN (+1) | 209 kDa |
| 437 | Trifunctional purine biosynthetic protein adenosine-3 OS=Homo sapiens GN=GART PE=1 SV=1 | sp\|P22102\|PUR2_HUMAN (+2) | 108 kDa |
| 438 | Protein phosphatase 1F OS=Homo sapiens GN=PPM1F PE=1 SV=3 | sp\|P49593\|PPM1F_HUMAN (+2) | 50 kDa |
| 439 | Zinc finger protein 768 OS=Homo sapiens GN=ZNF768 PE=1 SV=2 | sp\|Q9H5H4\|ZN768_HUMAN (+2) | 60 kDa |
| 440 | Leucine-rich repeat flightless-interacting protein 2 OS=Homo sapiens GN=LRRFIP2 PE=2 SV=1 | tr\|A0A1S5UZ17\|A0A1S5UZ17_HUMAN | 49 kDa |
| 441 | Transcription initiation factor TFIID subunit 7 OS=Homo sapiens GN=TAF7 PE=1 SV=1 | sp\|Q15545\|TAF7_HUMAN | 40 kDa |
| 442 | Importin subunit alpha-5 OS=Homo sapiens GN=KPNA1 PE=1 SV=3 | sp\|P52294\|IMA5_HUMAN (+1) | 60 kDa |
| 443 | Poly(rC)-binding protein 2 OS=Homo sapiens GN=PCBP2 PE=1 SV=1 | tr\|F8VZX2\|F8VZX2_HUMAN | 34 kDa |
| 444 | Nucleolar MIF4G domain-containing protein 1 OS=Homo sapiens GN=NOM1 PE=1 SV=1 | sp\|Q5C9Z4\|NOM1_HUMAN | 96 kDa |
| 445 | Methionine--tRNA ligase, mitochondrial OS=Homo sapiens GN=MARS2 PE=1 SV=2 | sp\|Q96GW9\|SYMM_HUMAN (+1) | 67 kDa |
| 446 | Probable histidine--tRNA ligase, mitochondrial OS=Homo sapiens GN=HARS2 PE=1 SV=1 | sp\|P49590\|SYHM_HUMAN (+1) | 57 kDa |
| 447 | Heterogeneous nuclear ribonucleoprotein A/B OS=Homo sapiens GN=HNRNPAB PE=1 SV=1 | tr\|D6RBZ0\|D6RBZ0_HUMAN (+1) | 36 kDa |
| 448 | Interferon-induced, double-stranded RNA-activated protein kinase OS=Homo sapiens GN=EIF2AK2 PE=1 SV=2 | sp\|P19525\|E2AK2_HUMAN (+1) | 62 kDa |
| 449 | Zinc finger protein ZPR1 OS=Homo sapiens GN=ZPR1 PE=1 SV=1 | sp\|O75312\|ZPR1_HUMAN (+2) | 51 kDa |
| 450 | RNA-binding motif protein, X chromosome OS=Homo sapiens GN=RBMX PE=1 SV=3 | sp\|P38159\|RBMX_HUMAN (+2) | 42 kDa |
| 451 | Zinc finger protein 70 OS=Homo sapiens GN=ZNF70 PE=2 SV=2 | sp\|Q9UC06\|ZNF70_HUMAN (+1) | 51 kDa |
| 452 | 40S ribosomal protein S7 OS=Homo sapiens GN=RPS7 PE=1 SV=1 | sp\|P62081\|RS7_HUMAN | 22 kDa |
| 453 | Mitochondrial ribonuclease P protein 1 OS=Homo sapiens GN=TRMT10C PE=1 SV=2 | sp\|Q7L0Y3\|MRRP1_HUMAN (+1) | 47 kDa |
| 454 | Protein scribble homolog OS=Homo sapiens GN=SCRIB PE=1 SV=4 | sp\|Q14160\|SCRIB_HUMAN (+2) | 175 kDa |
| 455 | L-lactate dehydrogenase B chain OS=Homo sapiens GN=LDHB PE=1 SV=2 | sp\|P07195\|LDHB_HUMAN (+1) | 37 kDa |
| 456 | 60S ribosomal protein L22 OS=Homo sapiens GN=RPL22 PE=1 SV=2 | sp\|P35268\|RL22_HUMAN (+1) | 15 kDa |
| 457 | Glutathione S-transferase Mu 3 OS=Homo sapiens GN=GSTM3 PE=1 SV=3 | sp\|P21266\|GSTM3_HUMAN (+2) | 27 kDa |
| 458 | Phosphatidylinositol 4-kinase type 2-alpha OS=Homo sapiens GN=PI4K2A PE=1 SV=1 | sp\|Q9BTU6\|P4K2A_HUMAN (+2) | 54 kDa |
| 459 | DnaJ homolog subfamily A member 2 OS=Homo sapiens GN=DNAJA2 PE=1 SV=1 | sp\|O60884\|DNJA2_HUMAN (+1) | 46 kDa |
| 460 | Transcription factor Dp-1 OS=Homo sapiens GN=TFDP1 PE=1 SV=1 | sp\|Q14186\|TFDP1_HUMAN (+2) | 45 kDa |
| 461 | Non-structural maintenance of chromosomes element 4 homolog A OS=Homo sapiens GN=NSMCE4A PE=1 SV=2 | sp\|Q9NXX6\|NSE4A_HUMAN (+1) | 44 kDa |
| 462 | Histone-lysine N-methyltransferase SETD7 OS=Homo sapiens GN=SETD7 PE=1 SV=1 | sp\|Q8WTS6\|SETD7_HUMAN (+1) | 41 kDa |
| 463 | Integrin-linked kinase-associated serine/threonine phosphatase 2C OS=Homo sapiens GN=ILKAP PE=1 SV=1 | sp\|Q9H0C8\|ILKAP_HUMAN | 43 kDa |
| 464 | Probable ATP-dependent RNA helicase DDX47 OS=Homo sapiens GN=DDX47 PE=1 SV=1 | sp\|Q9H0S4\|DDX47_HUMAN (+4) | 51 kDa |
| 465 | Septin-11 OS=Homo sapiens GN=SEPT11 PE=1 SV=3 | sp\|Q9NVA2\|SEP11_HUMAN | 49 kDa |
| 466 | Protein LTV1 homolog OS=Homo sapiens GN=LTV1 PE=1 SV=1 | sp\|Q96GA3\|LTV1_HUMAN | 55 kDa |
| 467 | Atlastin-2 OS=Homo sapiens GN=ATL2 PE=1 SV=2 | sp\|Q8NHH9\|ATLA2_HUMAN | 66 kDa |
| 468 | 60 kDa chaperonin (Fragment) OS=Homo sapiens GN=HSPD1 PE=4 SV=1 | tr\|B9VP24\|B9VP24_HUMAN | 20 kDa |
| 469 | Probable arginine--tRNA ligase, mitochondrial OS=Homo sapiens GN=RARS2 PE=1 SV=1 | sp\|Q5T160\|SYRM_HUMAN | 66 kDa |
| 470 | Carbonyl reductase [NADPH] 1 OS=Homo sapiens GN=CBR1 PE=1 SV=3 | sp\|P16152\|CBR1_HUMAN | 30 kDa |
| 471 | 26S proteasome regulatory subunit 8 OS=Homo sapiens GN=PSMC5 PE=1 SV=1 | sp\|P62195\|PRS8_HUMAN (+1) | 46 kDa |
| 472 | 60S ribosomal protein L11 OS=Homo sapiens GN=RPL11 PE=1 SV=2 | sp\|P62913\|RL11_HUMAN (+2) | 20 kDa |
| 473 | GA-binding protein alpha chain OS=Homo sapiens GN=GABPA PE=1 SV=1 | sp\|Q06546\|GABPA_HUMAN (+3) | 51 kDa |
| 474 | Nucleoredoxin OS=Homo sapiens GN=NXN PE=1 SV=2 | sp\|Q6DKJ4\|NXN_HUMAN | 48 kDa |
| 475 | Obg-like ATPase 1 OS=Homo sapiens GN=OLA1 PE=1 SV=2 | sp\|Q9NTK5\|OLA1_HUMAN (+1) | 45 kDa |
| 476 | Zinc finger Ran-binding domain-containing protein 2 OS=Homo sapiens GN=ZRANB2 PE=1 SV=2 | sp\|O95218\|ZRAB2_HUMAN | 37 kDa |
| 477 | 60S ribosomal protein L12 OS=Homo sapiens GN=RPL12 PE=1 SV=1 | sp\|P30050\|RL12_HUMAN | 18 kDa |
| 478 | Sodium/potassium-transporting ATPase subunit beta-3 OS=Homo sapiens GN=ATP1B3 PE=1 SV=1 | sp\|P54709\|AT1B3_HUMAN (+2) | 32 kDa |
| 479 | Sorting nexin-4 OS=Homo sapiens GN=SNX4 PE=1 SV=1 | sp\|O95219\|SNX4_HUMAN | 52 kDa |
| 480 | Nucleolar and coiled-body phosphoprotein 1 OS=Homo sapiens GN=NOLC1 PE=1 SV=2 | sp\|Q14978\|NOLC1_HUMAN (+2) | 74 kDa |

| # | Identified Proteins (716) | Accession Number | Molecular  weight |
| --- | --- | --- | --- |
| 481 | Plasma alpha-L-fucosidase OS=Homo sapiens GN=FUCA2 PE=1 SV=2 | sp\|Q9BTY2\|FUCO2_HUMAN | 54 kDa |
| 482 | Annexin A6 OS=Homo sapiens GN=ANXA6 PE=1 SV=3 | sp\|P08133\|ANXA6_HUMAN (+3) | 76 kDa |
| 483 | BRCA1-A complex subunit Abraxas 1 OS=Homo sapiens GN=ABRAXAS1 PE=1 SV=2 | sp\|Q6UWZ7\|ABRX1_HUMAN (+1) | 47 kDa |
| 484 | UBX domain-containing protein 6 OS=Homo sapiens GN=UBXN6 PE=1 SV=1 | sp\|Q9BZV1\|UBXN6_HUMAN (+1) | 50 kDa |
| 485 | Annexin A7 OS=Homo sapiens GN=ANXA7 PE=1 SV=3 | sp\|P20073\|ANXA7_HUMAN (+6) | 53 kDa |
| 486 | Importin subunit beta-1 OS=Homo sapiens GN=KPNB1 PE=1 SV=2 | sp\|Q14974\|IMB1_HUMAN (+1) | 97 kDa |
| 487 | Acid sphingomyelinase-like phosphodiesterase 3b OS=Homo sapiens GN=SMPDL3B PE=1 SV=2 | sp\|Q92485\|ASM3B_HUMAN (+1) | 51 kDa |
| 488 | Tubulin alpha-4A chain OS=Homo sapiens GN=TUBA4A PE=1 SV=1 | sp\|P68366\|TBA4A_HUMAN | 50 kDa |
| 489 | Lysosomal Pro-X carboxypeptidase OS=Homo sapiens GN=PRCP PE=1 SV=1 | sp\|P42785\|PCP_HUMAN (+2) | 56 kDa |
| 490 | Interferon regulatory factor 3 OS=Homo sapiens GN=IRF3 PE=1 SV=1 | sp\|Q14653\|IRF3_HUMAN (+1) | 47 kDa |
| 491 | PHD finger protein 10 OS=Homo sapiens GN=PHF10 PE=1 SV=3 | sp\|Q8WUB8\|PHF10_HUMAN (+1) | 56 kDa |
| 492 | Nuclear fragile X mental retardation-interacting protein 2 OS=Homo sapiens GN=NUFIP2 PE=1 SV=1 | sp\|Q7Z417\|NUFP2_HUMAN | 76 kDa |
| 493 | Alpha-aminoadipic semialdehyde synthase, mitochondrial OS=Homo sapiens GN=AASS PE=1 SV=1 | sp\|Q9UDR5\|AASS_HUMAN (+1) | 102 kDa |
| 494 | Peroxisomal membrane protein PEX14 OS=Homo sapiens GN=PEX14 PE=1 SV=1 | sp\|O75381\|PEX14_HUMAN | 41 kDa |
| 495 | Glutamyl-tRNA(Gln) amidotransferase subunit B, mitochondrial OS=Homo sapiens GN=GATB PE=1 SV=1 | sp\|O75879\|GATB_HUMAN (+1) | 62 kDa |
| 496 | Serine/threonine-protein phosphatase 2A 65 kDa regulatory subunit A beta isoform OS=Homo sapiens GN=PPP2R1B PE=1 SV=3 | sp\|P30154\|2AAB_HUMAN | 66 kDa |
| 497 | tRNA (guanine(37)-N1)-methyltransferase OS=Homo sapiens GN=TRMT5 PE=1 SV=2 | sp\|Q32P41\|TRM5_HUMAN | 58 kDa |
| 498 | Bifunctional arginine demethylase and lysyl-hydroxylase JMJD6 OS=Homo sapiens GN=JMJD6 PE=1 SV=1 | sp\|Q6NYC1\|JMJD6_HUMAN (+4) | 46 kDa |
| 499 | PC4 and SFRS1-interacting protein OS=Homo sapiens GN=PSIP1 PE=1 SV=1 | sp\|O75475\|PSIP1_HUMAN (+1) | 60 kDa |
| 500 | Eukaryotic translation initiation factor 4 gamma 2 OS=Homo sapiens GN=EIF4G2 PE=1 SV=1 | sp\|P78344\|IF4G2_HUMAN (+5) | 102 kDa |
| 501 | Protein LSM14 homolog A OS=Homo sapiens GN=LSM14A PE=1 SV=3 | sp\|Q8ND56\|LS14A_HUMAN | 51 kDa |
| 502 | tRNA dimethylallyltransferase, mitochondrial OS=Homo sapiens GN=TRIT1 PE=1 SV=1 | sp\|Q9H3H1\|MOD5_HUMAN (+6) | 53 kDa |
| 503 | Protein lunapark OS=Homo sapiens GN=LNP PE=1 SV=2 | sp\|Q9C0E8\|LNP_HUMAN (+3) | 48 kDa |
| 504 | tRNA-dihydrouridine(16/17) synthase [NAD(P)(+)]-like OS=Homo sapiens GN=DUS1L PE=1 SV=1 | sp\|Q6P1R4\|DUS1L_HUMAN (+4) | 53 kDa |
| 505 | Protein LSM14 homolog B OS=Homo sapiens GN=LSM14B PE=1 SV=1 | tr\|A0A0C4DFV2\|A0A0C4DFV2_HUMAN | 23 kDa |
| 506 | Caseinolytic peptidase B protein homolog OS=Homo sapiens GN=CLPB PE=1 SV=1 | tr\|F5GX99\|F5GX99_HUMAN | 55 kDa |
| 507 | cAMP-dependent protein kinase type II-beta regulatory subunit OS=Homo sapiens GN=PRKAR2B PE=1 SV=3 | sp\|P31323\|KAP3_HUMAN (+3) | 46 kDa |
| 508 | 60S ribosomal protein L30 OS=Homo sapiens GN=RPL30 PE=1 SV=2 | sp\|P62888\|RL30_HUMAN (+1) | 13 kDa |
| 509 | Cytoplasmic dynein 1 light intermediate chain 2 OS=Homo sapiens GN=DYNC1LI2 PE=1 SV=1 | sp\|O43237\|DC1L2_HUMAN (+1) | 54 kDa |
| 510 | Sodium/potassium-transporting ATPase subunit beta-1 OS=Homo sapiens GN=ATP1B1 PE=1 SV=1 | sp\|P05026\|AT1B1_HUMAN (+5) | 35 kDa |
| 511 | Endoplasmin OS=Homo sapiens GN=HSP90B1 PE=1 SV=1 | sp\|P14625\|ENPL_HUMAN (+3) | 92 kDa |
| 512 | Wilms tumor protein OS=Homo sapiens GN=WT1 PE=1 SV=2 | sp\|P19544\|WT1_HUMAN (+6) | 49 kDa |
| 513 | Elongation factor Tu, mitochondrial OS=Homo sapiens GN=TUFM PE=1 SV=2 | sp\|P49411\|EFTU_HUMAN | 50 kDa |
| 514 | Arf-GAP domain and FG repeat-containing protein 1 OS=Homo sapiens GN=AGFG1 PE=1 SV=2 | sp\|P52594\|AGFG1_HUMAN (+7) | 58 kDa |
| 515 | Aspartyl/asparaginyl beta-hydroxylase OS=Homo sapiens GN=ASPH PE=1 SV=3 | sp\|Q12797\|ASPH_HUMAN (+4) | 86 kDa |
| 516 | Serine/arginine-rich splicing factor 6 OS=Homo sapiens GN=SRSF6 PE=1 SV=2 | sp\|Q13247\|SRSF6_HUMAN (+2) | 40 kDa |
| 517 | Lipolysis-stimulated lipoprotein receptor OS=Homo sapiens GN=LSR PE=1 SV=4 | sp\|Q86X29\|LSR_HUMAN (+1) | 71 kDa |
| 518 | Abl interactor 1 OS=Homo sapiens GN=ABI1 PE=1 SV=4 | sp\|Q8IZP0\|ABI1_HUMAN (+3) | 55 kDa |
| 519 | Lysophosphatidylcholine acyltransferase 1 OS=Homo sapiens GN=LPCAT1 PE=1 SV=2 | sp\|Q8NF37\|PCAT1_HUMAN (+2) | 59 kDa |
| 520 | Protein DGCR14 OS=Homo sapiens GN=DGCR14 PE=1 SV=1 | sp\|Q96DF8\|DGC14_HUMAN | 53 kDa |
| 521 | Cytosolic non-specific dipeptidase OS=Homo sapiens GN=CNDP2 PE=1 SV=2 | sp\|Q96KP4\|CNDP2_HUMAN (+1) | 53 kDa |
| 522 | Zinc finger CCHC domain-containing protein 3 OS=Homo sapiens GN=ZCCHC3 PE=1 SV=1 | sp\|Q9NUD5\|ZCHC3_HUMAN | 44 kDa |
| 523 | Phosphatidylinositol 5-phosphate 4-kinase type-2 gamma OS=Homo sapiens GN=PIP4K2C PE=1 SV=3 | sp\|Q8TBX8\|PI42C_HUMAN | 47 kDa |
| 524 | Phosphatidylinositol-binding clathrin assembly protein OS=Homo sapiens GN=PICALM PE=1 SV=2 | sp\|Q13492\|PICAL_HUMAN (+2) | 71 kDa |
| 525 | Putative methyltransferase NSUN6 OS=Homo sapiens GN=NSUN6 PE=1 SV=1 | sp\|Q8TEA1\|NSUN6_HUMAN | 52 kDa |
| 526 | Splicing factor, proline- and glutamine-rich OS=Homo sapiens GN=SFPQ PE=1 SV=2 | sp\|P23246\|SFPQ_HUMAN (+3) | 76 kDa |
| 527 | Selenocysteine lyase OS=Homo sapiens GN=SCLY PE=1 SV=4 | sp\|Q96I15\|SCLY_HUMAN (+5) | 48 kDa |
| 528 | General transcription factor IIE subunit 1 OS=Homo sapiens GN=GTF2E1 PE=1 SV=2 | sp\|P29083\|T2EA_HUMAN (+2) | 49 kDa |
| 529 | UV excision repair protein RAD23 homolog A OS=Homo sapiens GN=RAD23A PE=1 SV=1 | sp\|P54725\|RD23A_HUMAN (+2) | 40 kDa |
| 530 | Autophagy protein 5 OS=Homo sapiens GN=ATG5 PE=1 SV=2 | sp\|Q9H1Y0\|ATG5_HUMAN (+2) | 32 kDa |
| 531 | Ubiquitin-conjugating enzyme E2 Q1 OS=Homo sapiens GN=UBE2Q1 PE=1 SV=1 | sp\|Q7Z7E8\|UB2Q1_HUMAN (+1) | 46 kDa |
| 532 | Na(+)/H(+) exchange regulatory cofactor NHE-RF1 OS=Homo sapiens GN=SLC9A3R1 PE=1 SV=4 | sp\|O14745\|NHRF1_HUMAN (+1) | 39 kDa |
| 533 | Ornithine aminotransferase, mitochondrial OS=Homo sapiens GN=OAT PE=1 SV=1 | sp\|P04181\|OAT_HUMAN (+1) | 49 kDa |
| 534 | [Pyruvate dehydrogenase [acetyl-transferring]]-phosphatase 1, mitochondrial OS=Homo sapiens GN=PDP1 PE=1 SV=3 | sp\|Q9P0J1\|PDP1_HUMAN (+4) | 61 kDa |
| 535 | Casein kinase I isoform gamma-2 OS=Homo sapiens GN=CSNK1G2 PE=1 SV=1 | sp\|P78368\|KC1G2_HUMAN | 47 kDa |
| 536 | TATA-binding protein-associated factor 2N OS=Homo sapiens GN=TAF15 PE=1 SV=1 | sp\|Q92804\|RBP56_HUMAN (+2) | 62 kDa |
| 537 | Atlastin-3 OS=Homo sapiens GN=ATL3 PE=1 SV=1 | sp\|Q6DD88\|ATLA3_HUMAN (+2) | 61 kDa |
| 538 | 60 kDa SS-A/Ro ribonucleoprotein OS=Homo sapiens GN=TROVE2 PE=1 SV=2 | sp\|P10155\|RO60_HUMAN (+1) | 61 kDa |
| 539 | 40S ribosomal protein S2 OS=Homo sapiens GN=RPS2 PE=1 SV=2 | sp\|P15880\|RS2_HUMAN (+3) | 31 kDa |
| 540 | Coxsackievirus and adenovirus receptor OS=Homo sapiens GN=CXADR PE=1 SV=1 | sp\|P78310\|CXAR_HUMAN | 40 kDa |

| # | Identified Proteins (716) | Accession Number | Molecular  weight |
| --- | --- | --- | --- |
| 541 | Heterochromatin protein 1-binding protein 3 OS=Homo sapiens GN=HP1BP3 PE=1 SV=1 | sp\|Q5SSJ5\|HP1B3_HUMAN (+1) | 61 kDa |
| 542 | BEN domain-containing protein 7 OS=Homo sapiens GN=BEND7 PE=1 SV=2 | sp\|Q8N7W2\|BEND7_HUMAN | 58 kDa |
| 543 | tRNA modification GTPase GTPBP3, mitochondrial OS=Homo sapiens GN=GTPBP3 PE=1 SV=2 | sp\|Q969Y2\|GTPB3_HUMAN | 52 kDa |
| 544 | Partitioning defective 6 homolog beta OS=Homo sapiens GN=PARD6B PE=1 SV=1 | sp\|Q9BYG5\|PAR6B_HUMAN | 41 kDa |
| 545 | Signal recognition particle subunit SRP68 OS=Homo sapiens GN=SRP68 PE=1 SV=2 | sp\|Q9UHB9\|SRP68_HUMAN | 71 kDa |
| 546 | Chloride channel CLIC-like protein 1 OS=Homo sapiens GN=CLCC1 PE=1 SV=1 | sp\|Q96S66\|CLCC1_HUMAN (+2) | 62 kDa |
| 547 | Delta-1-pyrroline-5-carboxylate dehydrogenase, mitochondrial OS=Homo sapiens GN=ALDH4A1 PE=1 SV=3 | sp\|P30038\|AL4A1_HUMAN (+1) | 62 kDa |
| 548 | Microtubule-associated protein 1A OS=Homo sapiens GN=MAP1A PE=1 SV=6 | sp\|P78559\|MAP1A_HUMAN (+3) | 305 kDa |
| 549 | DNA repair protein XRCC4 OS=Homo sapiens GN=XRCC4 PE=1 SV=2 | sp\|Q13426\|XRCC4_HUMAN (+3) | 38 kDa |
| 550 | Protein O-mannose kinase OS=Homo sapiens GN=POMK PE=1 SV=1 | sp\|Q9H5K3\|SG196_HUMAN | 40 kDa |
| 551 | Amidophosphoribosyltransferase OS=Homo sapiens GN=PPAT PE=1 SV=1 | sp\|Q06203\|PUR1_HUMAN (+2) | 57 kDa |
| 552 | Tubulin beta-2A chain OS=Homo sapiens GN=TUBB2A PE=1 SV=1 | sp\|Q13885\|TBB2A_HUMAN | 50 kDa |
| 553 | Fructose-bisphosphate aldolase A OS=Homo sapiens GN=ALDOA PE=1 SV=2 | sp\|P04075\|ALDOA_HUMAN (+3) | 39 kDa |
| 554 | YTH domain-containing family protein 2 OS=Homo sapiens GN=YTHDF2 PE=1 SV=2 | sp\|Q9Y5A9\|YTHD2_HUMAN (+1) | 62 kDa |
| 555 | 5'-3' exoribonuclease 2 OS=Homo sapiens GN=XRN2 PE=1 SV=1 | sp\|Q9H0D6\|XRN2_HUMAN (+3) | 109 kDa |
| 556 | Endoplasmic reticulum-Golgi intermediate compartment protein 3 OS=Homo sapiens GN=ERGIC3 PE=1 SV=1 | sp\|Q9Y282\|ERGI3_HUMAN (+5) | 43 kDa |
| 557 | Serine/arginine-rich splicing factor 7 OS=Homo sapiens GN=SRSF7 PE=1 SV=1 | sp\|Q16629\|SRSF7_HUMAN (+3) | 27 kDa |
| 558 | RISC-loading complex subunit TARBP2 OS=Homo sapiens GN=TARBP2 PE=1 SV=3 | sp\|Q15633\|TRBP2_HUMAN (+1) | 39 kDa |
| 559 | Sequestosome-1 OS=Homo sapiens GN=SQSTM1 PE=1 SV=1 | sp\|Q13501\|SQSTM_HUMAN | 48 kDa |
| 560 | 60S ribosomal protein L8 OS=Homo sapiens GN=RPL8 PE=1 SV=2 | sp\|P62917\|RL8_HUMAN (+1) | 28 kDa |
| 561 | 60S ribosomal protein L5 OS=Homo sapiens GN=RPL5 PE=1 SV=3 | sp\|P46777\|RL5_HUMAN (+4) | 34 kDa |
| 562 | Rab GDP dissociation inhibitor beta OS=Homo sapiens GN=GDI2 PE=1 SV=2 | sp\|P50395\|GDIB_HUMAN (+2) | 51 kDa |
| 563 | Serine/threonine-protein phosphatase 2A 56 kDa regulatory subunit epsilon isoform OS=Homo sapiens GN=PPP2R5E PE=1 SV=1 | sp\|Q16537\|2A5E_HUMAN (+3) | 55 kDa |
| 564 | Cell cycle control protein 50A OS=Homo sapiens GN=TMEM30A PE=1 SV=1 | sp\|Q9NV96\|CC50A_HUMAN (+2) | 41 kDa |
| 565 | Activating signal cointegrator 1 complex subunit 1, isoform CRA_a OS=Homo sapiens GN=ASCC1 PE=4 SV=1 | tr\|A0A024QZM0\|A0A024QZM0_HUMAN (+1) | 41 kDa |
| 566 | Zinc finger protein 385A OS=Homo sapiens GN=ZNF385A PE=1 SV=2 | sp\|Q96PM9\|Z385A_HUMAN | 40 kDa |
| 567 | BRISC complex subunit Abraxas 2 OS=Homo sapiens GN=ABRAXAS2 PE=1 SV=2 | sp\|Q15018\|ABRX2_HUMAN | 47 kDa |
| 568 | Programmed cell death protein 2-like OS=Homo sapiens GN=PDCD2L PE=1 SV=1 | sp\|Q9BRP1\|PDD2L_HUMAN | 39 kDa |
| 569 | Ceroid-lipofuscinosis neuronal protein 5 OS=Homo sapiens GN=CLN5 PE=1 SV=2 | sp\|O75503\|CLN5_HUMAN (+3) | 41 kDa |
| 570 | Tissue alpha-L-fucosidase OS=Homo sapiens GN=FUCA1 PE=1 SV=4 | sp\|P04066\|FUCO_HUMAN | 54 kDa |
| 571 | Coiled-coil domain-containing protein 130 OS=Homo sapiens GN=CCDC130 PE=1 SV=2 | sp\|P13994\|CC130_HUMAN | 45 kDa |
| 572 | Serine/threonine-protein kinase 3 OS=Homo sapiens GN=STK3 PE=1 SV=2 | sp\|Q13188\|STK3_HUMAN | 56 kDa |
| 573 | Glucoside xylosyltransferase 1 OS=Homo sapiens GN=GXYLT1 PE=1 SV=2 | sp\|Q4G148\|GXLT1_HUMAN | 51 kDa |
| 574 | Charged multivesicular body protein 7 OS=Homo sapiens GN=CHMP7 PE=1 SV=1 | sp\|Q8WUX9\|CHMP7_HUMAN (+3) | 51 kDa |
| 575 | Plakophilin-4 OS=Homo sapiens GN=PKP4 PE=1 SV=2 | sp\|Q99569\|PKP4_HUMAN (+2) | 132 kDa |
| 576 | TNF receptor-associated factor 4 OS=Homo sapiens GN=TRAF4 PE=1 SV=1 | sp\|Q9BUZ4\|TRAF4_HUMAN (+4) | 54 kDa |
| 577 | Polyadenylate-binding protein-interacting protein 1 OS=Homo sapiens GN=PAIP1 PE=1 SV=1 | sp\|Q9H074\|PAIP1_HUMAN (+2) | 54 kDa |
| 578 | Telomeric repeat-binding factor 2-interacting protein 1 OS=Homo sapiens GN=TERF2IP PE=1 SV=1 | sp\|Q9NYB0\|TE2IP_HUMAN | 44 kDa |
| 579 | Nuclear fragile X mental retardation-interacting protein 1 OS=Homo sapiens GN=NUFIP1 PE=1 SV=2 | sp\|Q9UHK0\|NUFP1_HUMAN | 56 kDa |
| 580 | Zinc finger protein 436 OS=Homo sapiens GN=ZNF436 PE=1 SV=2 | sp\|Q9C0F3\|ZN436_HUMAN (+1) | 54 kDa |
| 581 | ATP-dependent RNA helicase A OS=Homo sapiens GN=DHX9 PE=1 SV=4 | sp\|Q08211\|DHX9_HUMAN (+2) | 141 kDa |
| 582 | Sorting nexin-5 OS=Homo sapiens GN=SNX5 PE=1 SV=1 | sp\|Q9Y5X3\|SNX5_HUMAN (+4) | 47 kDa |
| 583 | Mitochondrial proton/calcium exchanger protein OS=Homo sapiens GN=LETM1 PE=1 SV=1 | sp\|O95202\|LETM1_HUMAN (+1) | 83 kDa |
| 584 | X-ray repair cross-complementing protein 6 OS=Homo sapiens GN=XRCC6 PE=1 SV=2 | sp\|P12956\|XRCC6_HUMAN (+1) | 70 kDa |
| 585 | NADPH:adrenodoxin oxidoreductase, mitochondrial OS=Homo sapiens GN=FDXR PE=1 SV=3 | sp\|P22570\|ADRO_HUMAN (+5) | 54 kDa |
| 586 | 60S ribosomal protein L10 OS=Homo sapiens GN=RPL10 PE=1 SV=4 | sp\|P27635\|RL10_HUMAN (+4) | 25 kDa |
| 587 | NADH dehydrogenase [ubiquinone] flavoprotein 1, mitochondrial OS=Homo sapiens GN=NDUFV1 PE=1 SV=4 | sp\|P49821\|NDUV1_HUMAN (+4) | 51 kDa |
| 588 | Histone deacetylase 1 OS=Homo sapiens GN=HDAC1 PE=1 SV=1 | sp\|Q13547\|HDAC1_HUMAN (+1) | 55 kDa |
| 589 | RNA demethylase ALKBH5 OS=Homo sapiens GN=ALKBH5 PE=1 SV=2 | sp\|Q6P6C2\|ALKB5_HUMAN | 44 kDa |
| 590 | E3 ubiquitin-protein ligase Hakai OS=Homo sapiens GN=CBLL1 PE=1 SV=1 | sp\|Q75N03\|HAKAI_HUMAN (+2) | 55 kDa |
| 591 | Zinc finger protein 397 OS=Homo sapiens GN=ZNF397 PE=1 SV=2 | sp\|Q8NF99\|ZN397_HUMAN | 61 kDa |
| 592 | DNA-directed DNA/RNA polymerase mu OS=Homo sapiens GN=POLM PE=1 SV=1 | sp\|Q9NP87\|DPOLM_HUMAN | 55 kDa |
| 593 | Something about silencing protein 10 OS=Homo sapiens GN=UTP3 PE=1 SV=1 | sp\|Q9NQZ2\|SAS10_HUMAN | 55 kDa |
| 594 | SPATS2-like protein OS=Homo sapiens GN=SPATS2L PE=1 SV=2 | sp\|Q9NUQ6\|SPS2L_HUMAN (+2) | 62 kDa |
| 595 | Elongin-A OS=Homo sapiens GN=ELOA PE=1 SV=2 | sp\|Q14241\|ELOA1_HUMAN (+1) | 90 kDa |
| 596 | U3 small nucleolar RNA-associated protein 18 homolog OS=Homo sapiens GN=UTP18 PE=1 SV=3 | sp\|Q9Y5J1\|UTP18_HUMAN | 62 kDa |
| 597 | Alanyl-tRNA editing protein Aarsd1 OS=Homo sapiens GN=AARSD1 PE=1 SV=2 | sp\|Q9BTE6\|AASD1_HUMAN (+2) | 45 kDa |
| 598 | Threonylcarbamoyladenosine tRNA methylthiotransferase OS=Homo sapiens GN=CDKAL1 PE=1 SV=1 | sp\|Q5VV42\|CDKAL_HUMAN | 65 kDa |
| 599 | WD repeat-containing protein 55 OS=Homo sapiens GN=WDR55 PE=1 SV=2 | sp\|Q9H6Y2\|WDR55_HUMAN | 42 kDa |
| 600 | Stomatin-like protein 2, mitochondrial OS=Homo sapiens GN=STOML2 PE=1 SV=1 | sp\|Q9UJZ1\|STML2_HUMAN | 39 kDa |

| # | Identified Proteins (716) | Accession Number | Molecular  weight |
| --- | --- | --- | --- |
| 601 | Ataxin-2-like protein OS=Homo sapiens GN=ATXN2L PE=1 SV=2 | sp\|Q8WWM7\|ATX2L_HUMAN (+1) | 113 kDa |
| 602 | T-complex protein 1 subunit alpha OS=Homo sapiens GN=TCP1 PE=1 SV=1 | sp\|P17987\|TCPA_HUMAN | 60 kDa |
| 603 | 60S ribosomal protein L13 OS=Homo sapiens GN=RPL13 PE=1 SV=4 | sp\|P26373\|RL13_HUMAN (+2) | 24 kDa |
| 604 | 60S ribosomal protein L3 OS=Homo sapiens GN=RPL3 PE=1 SV=2 | sp\|P39023\|RL3_HUMAN (+4) | 46 kDa |
| 605 | Perilipin-2 OS=Homo sapiens GN=PLIN2 PE=1 SV=2 | sp\|Q99541\|PLIN2_HUMAN (+2) | 48 kDa |
| 606 | Immunoglobulin kappa variable 2D-29 OS=Homo sapiens GN=IGKV2D-29 PE=3 SV=1 | sp\|A0A075B6S2\|KVD29_HUMAN (+2) | 13 kDa |
| 607 | Baculoviral IAP repeat-containing protein 5 OS=Homo sapiens GN=BIRC5 PE=1 SV=3 | sp\|O15392\|BIRC5_HUMAN (+3) | 16 kDa |
| 608 | Beta-2-glycoprotein 1 OS=Homo sapiens GN=APOH PE=1 SV=3 | sp\|P02749\|APOH_HUMAN (+2) | 38 kDa |
| 609 | Keratin, type II cytoskeletal 8 OS=Homo sapiens GN=KRT8 PE=1 SV=7 | sp\|P05787\|K2C8_HUMAN | 54 kDa |
| 610 | Non-specific lipid-transfer protein OS=Homo sapiens GN=SCP2 PE=1 SV=2 | sp\|P22307\|NLTP_HUMAN (+1) | 59 kDa |
| 611 | Succinate-semialdehyde dehydrogenase, mitochondrial OS=Homo sapiens GN=ALDH5A1 PE=1 SV=2 | sp\|P51649\|SSDH_HUMAN (+6) | 57 kDa |
| 612 | 40S ribosomal protein S24 OS=Homo sapiens GN=RPS24 PE=1 SV=1 | sp\|P62847\|RS24_HUMAN (+2) | 15 kDa |
| 613 | RNA-binding protein EWS OS=Homo sapiens GN=EWSR1 PE=1 SV=1 | sp\|Q01844\|EWS_HUMAN (+4) | 68 kDa |
| 614 | COBW domain-containing protein 6 OS=Homo sapiens GN=CBWD6 PE=3 SV=1 | sp\|Q4V339\|CBWD6_HUMAN (+3) | 44 kDa |
| 615 | Tubulin-specific chaperone cofactor E-like protein OS=Homo sapiens GN=TBCEL PE=1 SV=2 | sp\|Q5QJ74\|TBCEL_HUMAN (+1) | 48 kDa |
| 616 | Protein FAM199X OS=Homo sapiens GN=FAM199X PE=1 SV=1 | sp\|Q6PEV8\|F199X_HUMAN (+1) | 43 kDa |
| 617 | Uncharacterized protein C16orf59 OS=Homo sapiens GN=C16orf59 PE=1 SV=1 | sp\|Q7L2K0\|CP059_HUMAN | 46 kDa |
| 618 | Serine/threonine-protein kinase VRK2 OS=Homo sapiens GN=VRK2 PE=1 SV=3 | sp\|Q86Y07\|VRK2_HUMAN (+2) | 58 kDa |
| 619 | ERO1-like protein beta OS=Homo sapiens GN=ERO1B PE=1 SV=2 | sp\|Q86YB8\|ERO1B_HUMAN (+1) | 54 kDa |
| 620 | Phospholipase D3 OS=Homo sapiens GN=PLD3 PE=1 SV=1 | sp\|Q8IV08\|PLD3_HUMAN (+1) | 55 kDa |
| 621 | Synaptonemal complex protein SC65 OS=Homo sapiens GN=P3H4 PE=1 SV=1 | sp\|Q92791\|SC65_HUMAN (+1) | 50 kDa |
| 622 | Flt3-interacting zinc finger protein 1 OS=Homo sapiens GN=FIZ1 PE=1 SV=2 | sp\|Q96SL8\|FIZ1_HUMAN | 52 kDa |
| 623 | Cell division control protein 6 homolog OS=Homo sapiens GN=CDC6 PE=1 SV=1 | sp\|Q99741\|CDC6_HUMAN (+2) | 63 kDa |
| 624 | Synembryn-A OS=Homo sapiens GN=RIC8A PE=1 SV=3 | sp\|Q9NPQ8\|RIC8A_HUMAN (+1) | 60 kDa |
| 625 | Carbohydrate sulfotransferase 7 OS=Homo sapiens GN=CHST7 PE=1 SV=2 | sp\|Q9NS84\|CHST7_HUMAN | 54 kDa |
| 626 | Abl interactor 2 OS=Homo sapiens GN=ABI2 PE=1 SV=1 | sp\|Q9NYB9\|ABI2_HUMAN (+3) | 56 kDa |
| 627 | Anaphase-promoting complex subunit 7 OS=Homo sapiens GN=ANAPC7 PE=1 SV=4 | sp\|Q9UJX3\|APC7_HUMAN (+6) | 67 kDa |
| 628 | Multiple inositol polyphosphate phosphatase 1 OS=Homo sapiens GN=MINPP1 PE=1 SV=1 | sp\|Q9UNW1\|MINP1_HUMAN (+2) | 55 kDa |
| 629 | pre-rRNA processing protein FTSJ3 OS=Homo sapiens GN=FTSJ3 PE=1 SV=2 | sp\|Q8IY81\|SPB1_HUMAN (+1) | 97 kDa |
| 630 | Probable 28S rRNA (cytosine-C(5))-methyltransferase OS=Homo sapiens GN=NSUN5 PE=1 SV=2 | sp\|Q96P11\|NSUN5_HUMAN (+1) | 47 kDa |
| 631 | Calcium uptake protein 1, mitochondrial OS=Homo sapiens GN=MICU1 PE=1 SV=1 | sp\|Q9BPX6\|MICU1_HUMAN (+2) | 54 kDa |
| 632 | Putative methyltransferase C9orf114 OS=Homo sapiens GN=SPOUT1 PE=1 SV=3 | sp\|Q5T280\|CI114_HUMAN | 42 kDa |
| 633 | NADP-dependent malic enzyme OS=Homo sapiens GN=ME1 PE=1 SV=1 | sp\|P48163\|MAOX_HUMAN (+1) | 64 kDa |
| 634 | MAP7 domain-containing protein 1 OS=Homo sapiens GN=MAP7D1 PE=1 SV=1 | sp\|Q3KQU3\|MA7D1_HUMAN (+3) | 93 kDa |
| 635 | TLD domain-containing protein 1 OS=Homo sapiens GN=TLDC1 PE=1 SV=2 | sp\|Q6P9B6\|TLDC1_HUMAN (+1) | 51 kDa |
| 636 | Lysine--tRNA ligase OS=Homo sapiens GN=KARS PE=1 SV=3 | sp\|Q15046\|SYK_HUMAN | 68 kDa |
| 637 | CCR4-NOT transcription complex subunit 11 OS=Homo sapiens GN=CNOT11 PE=1 SV=1 | sp\|Q9UKZ1\|CNO11_HUMAN | 55 kDa |
| 638 | Protein disulfide-isomerase A5 OS=Homo sapiens GN=PDIA5 PE=1 SV=1 | sp\|Q14554\|PDIA5_HUMAN | 60 kDa |
| 639 | Ribosome biogenesis regulatory protein homolog OS=Homo sapiens GN=RRS1 PE=1 SV=2 | sp\|Q15050\|RRS1_HUMAN | 41 kDa |
| 640 | La-related protein 4B OS=Homo sapiens GN=LARP4B PE=1 SV=3 | sp\|Q92615\|LAR4B_HUMAN | 81 kDa |
| 641 | Protein Njmu-R1 OS=Homo sapiens GN=C17orf75 PE=1 SV=2 | sp\|Q9HAS0\|NJMU_HUMAN (+1) | 45 kDa |
| 642 | Insulin receptor substrate 4 OS=Homo sapiens GN=IRS4 PE=1 SV=1 | sp\|O14654\|IRS4_HUMAN | 134 kDa |
| 643 | Microfibrillar-associated protein 1 OS=Homo sapiens GN=MFAP1 PE=1 SV=2 | sp\|P55081\|MFAP1_HUMAN | 52 kDa |
| **644** | **E3 ubiquitin-protein ligase XIAP OS=Homo sapiens GN=XIAP PE=1 SV=2** | **sp\|P98170\|XIAP_HUMAN** | **57 kDa** |
| 645 | Spermatogenesis-defective protein 39 homolog OS=Homo sapiens GN=VIPAS39 PE=1 SV=1 | sp\|Q9H9C1\|SPE39_HUMAN (+3) | 57 kDa |
| 646 | Evolutionarily conserved signaling intermediate in Toll pathway, mitochondrial OS=Homo sapiens GN=ECSIT PE=1 SV=1 | sp\|Q9BQ95\|ECSIT_HUMAN (+1) | 49 kDa |
| 647 | Cyclin-dependent kinase 9 OS=Homo sapiens GN=CDK9 PE=1 SV=3 | sp\|P50750\|CDK9_HUMAN | 43 kDa |
| 648 | Tripeptidyl-peptidase 1 OS=Homo sapiens GN=TPP1 PE=1 SV=2 | sp\|O14773\|TPP1_HUMAN (+6) | 61 kDa |
| 649 | Protein regulator of cytokinesis 1 OS=Homo sapiens GN=PRC1 PE=1 SV=2 | sp\|O43663\|PRC1_HUMAN (+1) | 72 kDa |
| 650 | Slit homolog 2 protein OS=Homo sapiens GN=SLIT2 PE=1 SV=1 | sp\|O94813\|SLIT2_HUMAN (+3) | 170 kDa |
| 651 | Mitochondrial import receptor subunit TOM70 OS=Homo sapiens GN=TOMM70 PE=1 SV=1 | sp\|O94826\|TOM70_HUMAN (+1) | 67 kDa |
| 652 | Activator of 90 kDa heat shock protein ATPase homolog 1 OS=Homo sapiens GN=AHSA1 PE=1 SV=1 | sp\|O95433\|AHSA1_HUMAN (+3) | 38 kDa |
| 653 | Cathepsin D OS=Homo sapiens GN=CTSD PE=1 SV=1 | sp\|P07339\|CATD_HUMAN (+10) | 45 kDa |
| 654 | Dihydrolipoyl dehydrogenase, mitochondrial OS=Homo sapiens GN=DLD PE=1 SV=2 | sp\|P09622\|DLDH_HUMAN (+4) | 54 kDa |
| 655 | Poly [ADP-ribose] polymerase 1 OS=Homo sapiens GN=PARP1 PE=1 SV=4 | sp\|P09874\|PARP1_HUMAN (+3) | 113 kDa |
| 656 | Beta-galactosidase OS=Homo sapiens GN=GLB1 PE=1 SV=2 | sp\|P16278\|BGAL_HUMAN (+3) | 76 kDa |
| 657 | Methionine aminopeptidase 2 OS=Homo sapiens GN=METAP2 PE=1 SV=1 | sp\|P50579\|MAP2_HUMAN (+4) | 53 kDa |
| 658 | N-sulphoglucosamine sulphohydrolase OS=Homo sapiens GN=SGSH PE=1 SV=1 | sp\|P51688\|SPHM_HUMAN (+1) | 57 kDa |
| 659 | Transcriptional activator protein Pur-alpha OS=Homo sapiens GN=PURA PE=1 SV=2 | sp\|Q00577\|PURA_HUMAN (+4) | 35 kDa |
| 660 | E3 ubiquitin-protein ligase TRIP12 OS=Homo sapiens GN=TRIP12 PE=1 SV=1 | sp\|Q14669\|TRIPC_HUMAN | 220 kDa |

| # | Identified Proteins (716) | Accession Number | Molecular  weight |
| --- | --- | --- | --- |
| 661 | Caspase-8 OS=Homo sapiens GN=CASP8 PE=1 SV=1 | sp\|Q14790\|CASP8_HUMAN (+10) | 55 kDa |
| 662 | Protein phosphatase 1 regulatory subunit 7 OS=Homo sapiens GN=PPP1R7 PE=1 SV=1 | sp\|Q15435\|PP1R7_HUMAN (+5) | 42 kDa |
| 663 | Drebrin OS=Homo sapiens GN=DBN1 PE=1 SV=4 | sp\|Q16643\|DREB_HUMAN (+1) | 71 kDa |
| 664 | Inhibitor of nuclear factor kappa-B kinase-interacting protein OS=Homo sapiens GN=IKBIP PE=1 SV=1 | sp\|Q70UQ0\|IKIP_HUMAN | 39 kDa |
| 665 | Heparan sulfate 2-O-sulfotransferase 1 OS=Homo sapiens GN=HS2ST1 PE=1 SV=1 | sp\|Q7LGA3\|HS2ST_HUMAN (+1) | 42 kDa |
| 666 | Centrosomal protein of 57 kDa OS=Homo sapiens GN=CEP57 PE=1 SV=2 | sp\|Q86XR8\|CEP57_HUMAN (+3) | 57 kDa |
| 667 | Inactive serine/threonine-protein kinase VRK3 OS=Homo sapiens GN=VRK3 PE=1 SV=2 | sp\|Q8IV63\|VRK3_HUMAN (+2) | 53 kDa |
| 668 | PHD finger protein 6 OS=Homo sapiens GN=PHF6 PE=1 SV=1 | sp\|Q8IWS0\|PHF6_HUMAN (+2) | 41 kDa |
| 669 | Disks large homolog 3 OS=Homo sapiens GN=DLG3 PE=1 SV=2 | sp\|Q92796\|DLG3_HUMAN (+2) | 90 kDa |
| 670 | E3 ubiquitin-protein ligase TRIM11 OS=Homo sapiens GN=TRIM11 PE=1 SV=2 | sp\|Q96F44\|TRI11_HUMAN | 53 kDa |
| 671 | Zinc finger protein 703 OS=Homo sapiens GN=ZNF703 PE=1 SV=1 | sp\|Q9H7S9\|ZN703_HUMAN | 58 kDa |
| 672 | T-complex protein 11-like protein 1 OS=Homo sapiens GN=TCP11L1 PE=1 SV=1 | sp\|Q9NUJ3\|T11L1_HUMAN (+2) | 57 kDa |
| 673 | Alpha-parvin OS=Homo sapiens GN=PARVA PE=1 SV=1 | sp\|Q9NVD7\|PARVA_HUMAN (+3) | 42 kDa |
| 674 | Ataxin-10 OS=Homo sapiens GN=ATXN10 PE=1 SV=1 | sp\|Q9UBB4\|ATX10_HUMAN (+2) | 53 kDa |
| 675 | Nischarin OS=Homo sapiens GN=NISCH PE=1 SV=3 | sp\|Q9Y2I1\|NISCH_HUMAN (+2) | 167 kDa |
| 676 | Basic leucine zipper and W2 domain-containing protein 2 OS=Homo sapiens GN=BZW2 PE=1 SV=1 | sp\|Q9Y6E2\|BZW2_HUMAN (+4) | 48 kDa |
| 677 | WW domain-binding protein 4 OS=Homo sapiens GN=WBP4 PE=1 SV=1 | sp\|O75554\|WBP4_HUMAN (+2) | 43 kDa |
| 678 | Ran GTPase-activating protein 1 OS=Homo sapiens GN=RANGAP1 PE=1 SV=1 | sp\|P46060\|RAGP1_HUMAN (+1) | 64 kDa |
| 679 | 60S ribosomal protein L24 OS=Homo sapiens GN=RPL24 PE=1 SV=1 | sp\|P83731\|RL24_HUMAN (+3) | 18 kDa |
| 680 | Poly(rC)-binding protein 1 OS=Homo sapiens GN=PCBP1 PE=1 SV=2 | sp\|Q15365\|PCBP1_HUMAN (+1) | 37 kDa |
| 681 | NAD-dependent malic enzyme, mitochondrial OS=Homo sapiens GN=ME2 PE=1 SV=1 | sp\|P23368\|MAOM_HUMAN (+6) | 65 kDa |
| 682 | Prenylcysteine oxidase 1 OS=Homo sapiens GN=PCYOX1 PE=1 SV=3 | sp\|Q9UHG3\|PCYOX_HUMAN (+3) | 57 kDa |
| 683 | Pyruvate kinase PKM OS=Homo sapiens GN=PKM PE=1 SV=4 | sp\|P14618\|KPYM_HUMAN (+2) | 58 kDa |
| 684 | DNA primase small subunit OS=Homo sapiens GN=PRIM1 PE=1 SV=1 | sp\|P49642\|PRI1_HUMAN (+2) | 50 kDa |
| 685 | Poly(A) RNA polymerase GLD2 OS=Homo sapiens GN=PAPD4 PE=1 SV=1 | sp\|Q6PIY7\|GLD2_HUMAN (+1) | 56 kDa |
| 686 | Septin-7 OS=Homo sapiens GN=SEPT7 PE=1 SV=2 | sp\|Q16181\|SEPT7_HUMAN (+2) | 51 kDa |
| 687 | Serine/threonine-protein kinase 25 OS=Homo sapiens GN=STK25 PE=1 SV=1 | sp\|O00506\|STK25_HUMAN (+1) | 48 kDa |
| 688 | Catenin delta-1 OS=Homo sapiens GN=CTNND1 PE=1 SV=1 | sp\|O60716\|CTND1_HUMAN (+6) | 108 kDa |
| 689 | Low-density lipoprotein receptor-related protein 4 OS=Homo sapiens GN=LRP4 PE=1 SV=4 | sp\|O75096\|LRP4_HUMAN | 212 kDa |
| 690 | Signal recognition particle subunit SRP72 OS=Homo sapiens GN=SRP72 PE=1 SV=3 | sp\|O76094\|SRP72_HUMAN (+7) | 75 kDa |
| 691 | Ribonuclease inhibitor OS=Homo sapiens GN=RNH1 PE=1 SV=2 | sp\|P13489\|RINI_HUMAN (+2) | 50 kDa |
| 692 | Protein farnesyltransferase/geranylgeranyltransferase type-1 subunit alpha OS=Homo sapiens GN=FNTA PE=1 SV=1 | sp\|P49354\|FNTA_HUMAN (+2) | 44 kDa |
| 693 | 60S ribosomal protein L10a OS=Homo sapiens GN=RPL10A PE=1 SV=2 | sp\|P62906\|RL10A_HUMAN (+2) | 25 kDa |
| 694 | Protein Red OS=Homo sapiens GN=IK PE=1 SV=3 | sp\|Q13123\|RED_HUMAN (+3) | 66 kDa |
| 695 | Tubulin beta-3 chain OS=Homo sapiens GN=TUBB3 PE=1 SV=2 | sp\|Q13509\|TBB3_HUMAN | 50 kDa |
| 696 | Peroxisomal acyl-coenzyme A oxidase 1 OS=Homo sapiens GN=ACOX1 PE=1 SV=3 | sp\|Q15067\|ACOX1_HUMAN (+2) | 74 kDa |
| 697 | Intracellular hyaluronan-binding protein 4 OS=Homo sapiens GN=HABP4 PE=1 SV=1 | sp\|Q5JVS0\|HABP4_HUMAN | 46 kDa |
| 698 | Armadillo repeat-containing protein 6 OS=Homo sapiens GN=ARMC6 PE=1 SV=2 | sp\|Q6NXE6\|ARMC6_HUMAN (+2) | 54 kDa |
| 699 | Beta-1,3-glucosyltransferase OS=Homo sapiens GN=B3GLCT PE=1 SV=2 | sp\|Q6Y288\|B3GLT_HUMAN | 57 kDa |
| 700 | Uncharacterized protein C14orf80 OS=Homo sapiens GN=C14orf80 PE=2 SV=2 | sp\|Q86SX3\|CN080_HUMAN | 54 kDa |
| 701 | Transcription factor GATA-6 OS=Homo sapiens GN=GATA6 PE=1 SV=2 | sp\|Q92908\|GATA6_HUMAN (+1) | 60 kDa |
| 702 | Peptidyl-prolyl cis-trans isomerase FKBP10 OS=Homo sapiens GN=FKBP10 PE=1 SV=1 | sp\|Q96AY3\|FKB10_HUMAN (+3) | 64 kDa |
| 703 | Zinc finger protein 48 OS=Homo sapiens GN=ZNF48 PE=1 SV=2 | sp\|Q96MX3\|ZNF48_HUMAN (+2) | 68 kDa |
| 704 | Replication initiator 1 OS=Homo sapiens GN=REPIN1 PE=1 SV=1 | sp\|Q9BWE0\|REPI1_HUMAN (+6) | 64 kDa |
| 705 | Sentrin-specific protease 2 OS=Homo sapiens GN=SENP2 PE=1 SV=3 | sp\|Q9HC62\|SENP2_HUMAN (+4) | 68 kDa |
| 706 | Leucine-rich repeat protein SHOC-2 OS=Homo sapiens GN=SHOC2 PE=1 SV=2 | sp\|Q9UQ13\|SHOC2_HUMAN | 65 kDa |
| 707 | Torsin A interacting protein 2, isoform CRA_b OS=Homo sapiens GN=TOR1AIP2 PE=4 SV=1 | tr\|A0A024R957\|A0A024R957_HUMAN | 51 kDa |
| 708 | Probable ATP-dependent RNA helicase DDX41 OS=Homo sapiens GN=DDX41 PE=1 SV=2 | sp\|Q9UJV9\|DDX41_HUMAN (+3) | 70 kDa |
| 709 | Histone H1.4 OS=Homo sapiens GN=HIST1H1E PE=1 SV=2 | sp\|P10412\|H14_HUMAN (+6) | 22 kDa |
| 710 | Heterogeneous nuclear ribonucleoproteins A2/B1 OS=Homo sapiens GN=HNRNPA2B1 PE=1 SV=2 | sp\|P22626\|ROA2_HUMAN | 37 kDa |
| 711 | Glycogen synthase kinase-3 alpha OS=Homo sapiens GN=GSK3A PE=1 SV=2 | sp\|P49840\|GSK3A_HUMAN (+3) | 51 kDa |
| 712 | Occludin OS=Homo sapiens GN=OCLN PE=1 SV=1 | sp\|Q16625\|OCLN_HUMAN (+2) | 59 kDa |
| 713 | Tetratricopeptide repeat protein 31 OS=Homo sapiens GN=TTC31 PE=1 SV=3 | sp\|Q49AM3\|TTC31_HUMAN (+2) | 57 kDa |
| 714 | Protein SCAI OS=Homo sapiens GN=SCAI PE=1 SV=2 | sp\|Q8N9R8\|SCAI_HUMAN | 70 kDa |
| 715 | Protein disulfide-isomerase TMX3 OS=Homo sapiens GN=TMX3 PE=1 SV=2 | sp\|Q96JJ7\|TMX3_HUMAN | 52 kDa |
| 716 | Spastin OS=Homo sapiens GN=SPAST PE=1 SV=1 | sp\|Q9UBP0\|SPAST_HUMAN (+2) | 67 kDa |
