# Supplementary figures and images for: Mutual regulation between OGT and XIAP to control colon cancer cell growth and invasion
Source: Cell Death Dis. 2020 Sep 29;11(9):815. doi: 10.1038/s41419-020-02999-5 (PMC7525441; doi:10.1038/s41419-020-02999-5)

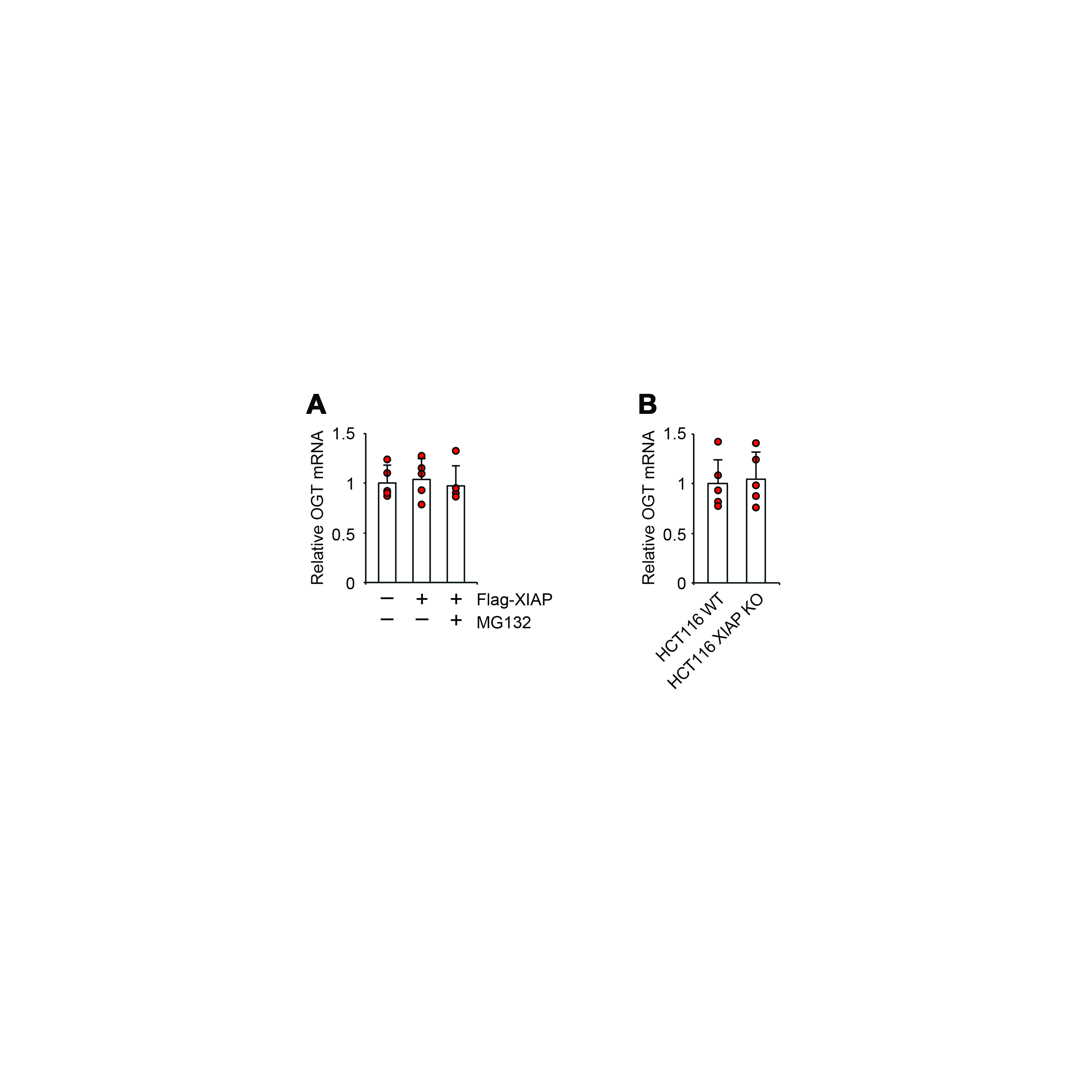

Supplement: Supplementary file 2 — Supplementary Fig. S1 Effects of XIAP overexpression on OGT mRNA level. [file 41419_2020_2999_MOESM2_ESM.tif]

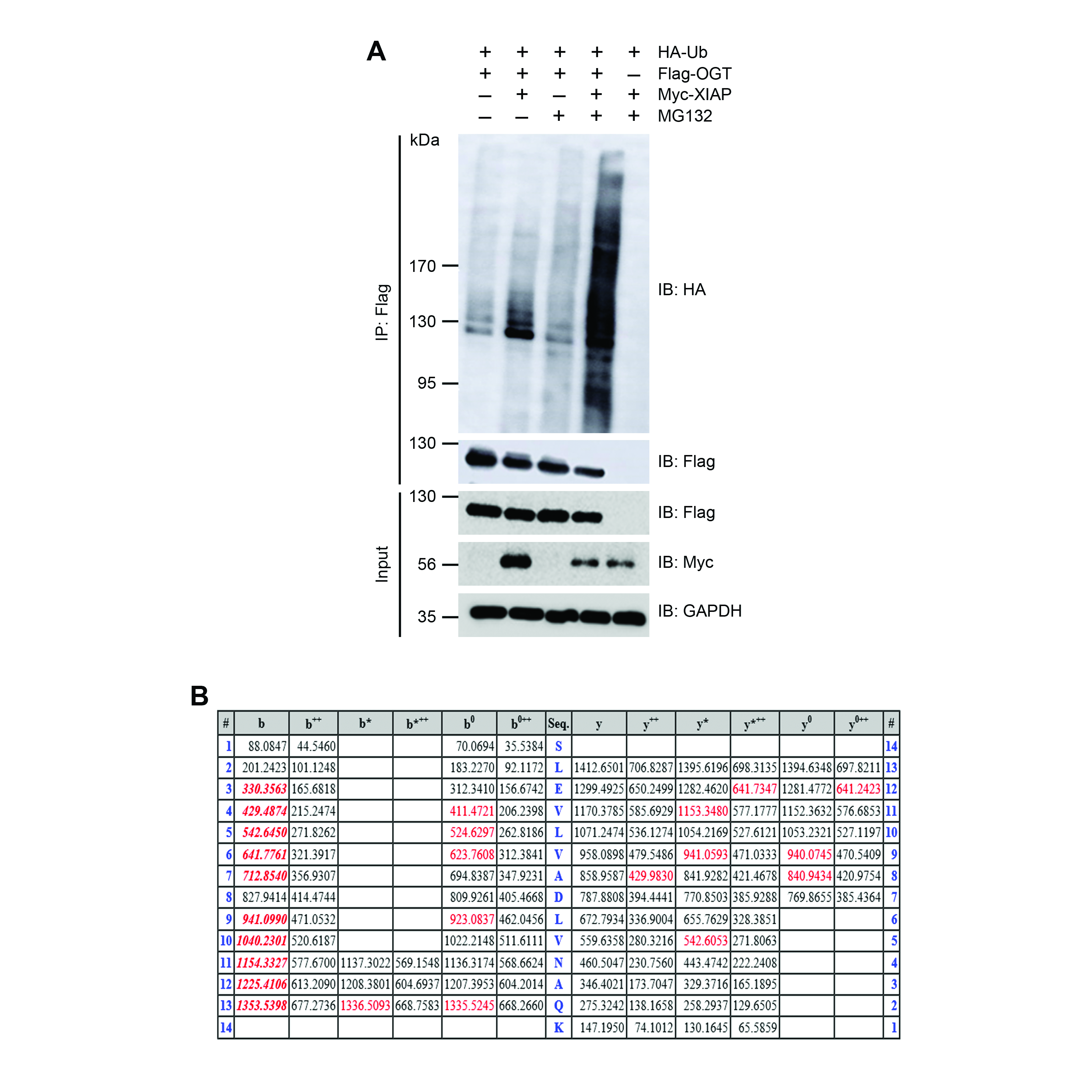

Supplement: Supplementary file 3 — Supplementary Fig. S2 OGT is a substrate of XIAP and mediates the O-GlcNAcylation of XIAP in HEK293 cells. [file 41419_2020_2999_MOESM3_ESM.tif]

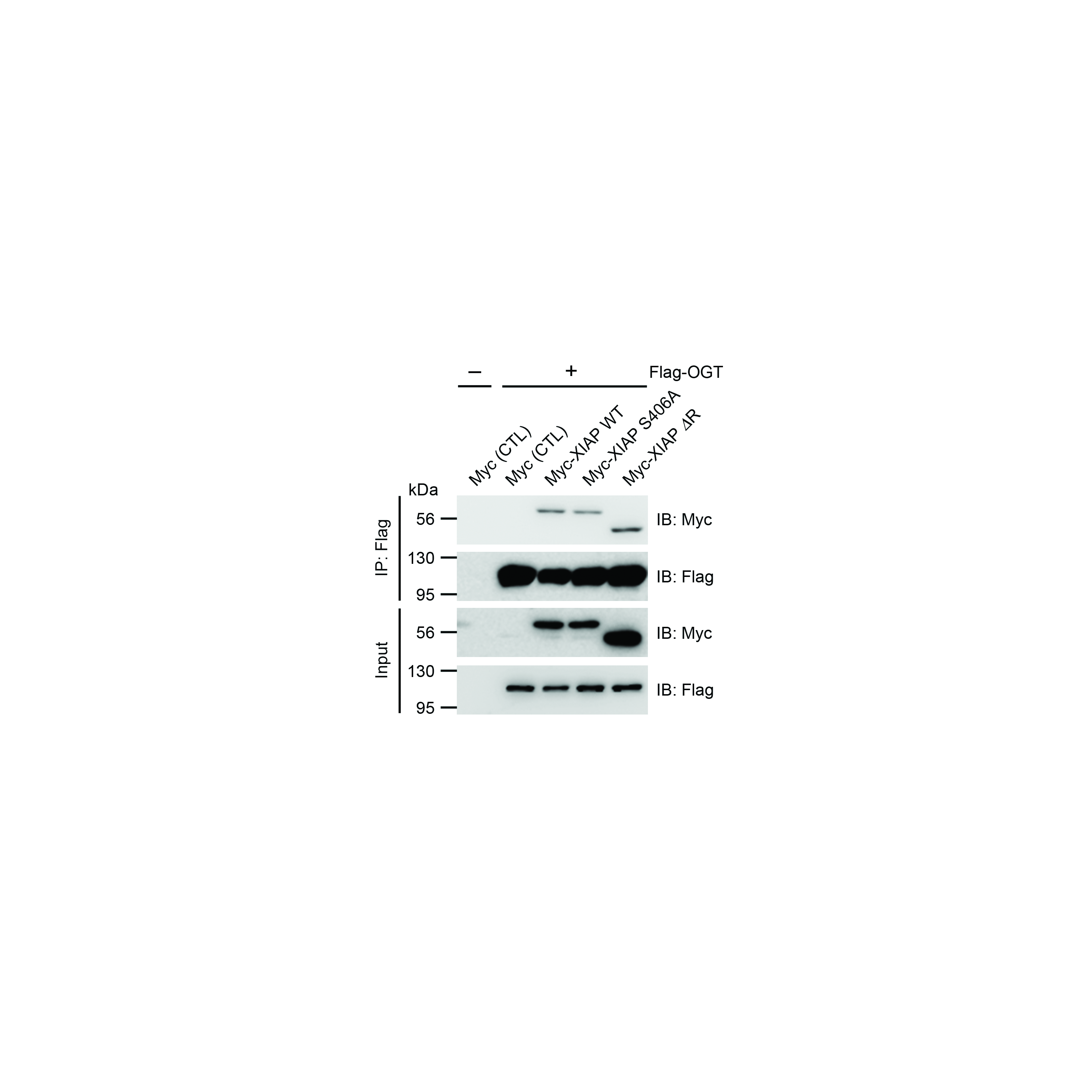

Supplement: Supplementary file 4 — Supplementary Fig. S3 The substitution of Serine 406 to alanine and the deletion of the RING domains in XIAP does not affect its interactions with OGT. [file 41419_2020_2999_MOESM4_ESM.tif]

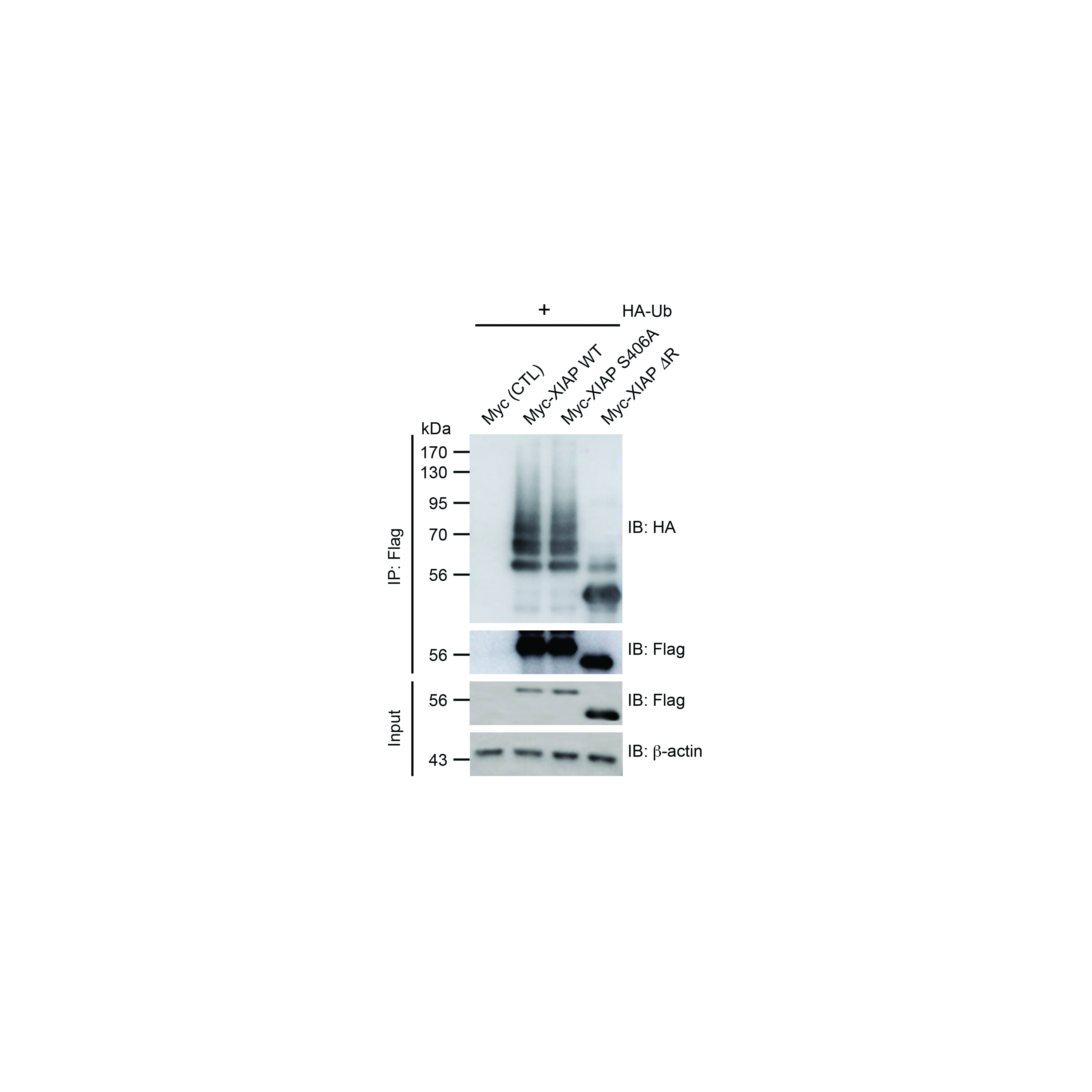

Supplement: Supplementary file 5 — Supplementary Fig. S4 The substitution of Serine 406 to alanine in XIAP does not affect the auto-ubiquitination. [file 41419_2020_2999_MOESM5_ESM.tif]

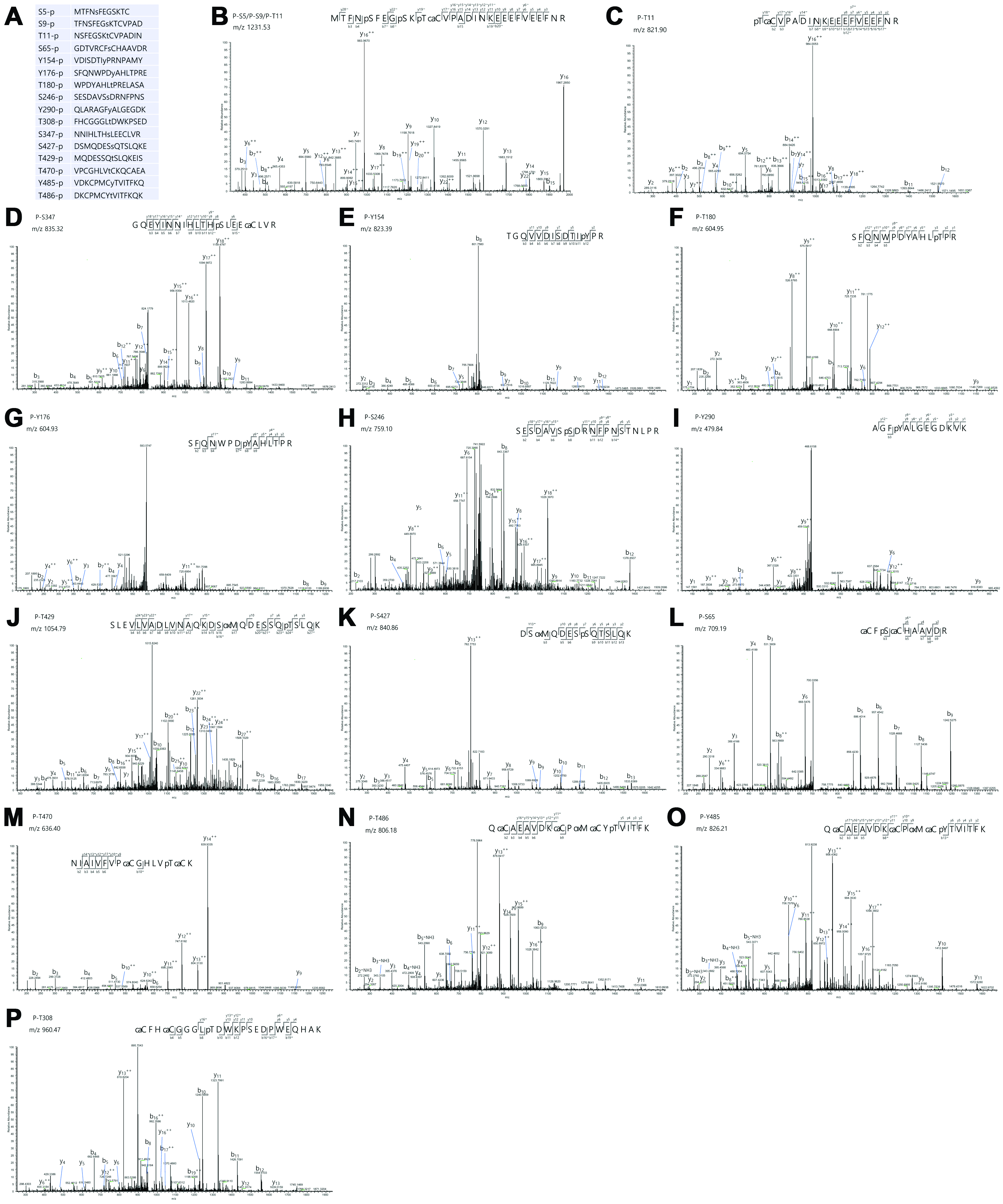

Supplement: Supplementary file 6 — Supplementary Fig. S5 MS analysis for phosphorylation residues on XIAP. [file 41419_2020_2999_MOESM6_ESM.tif]

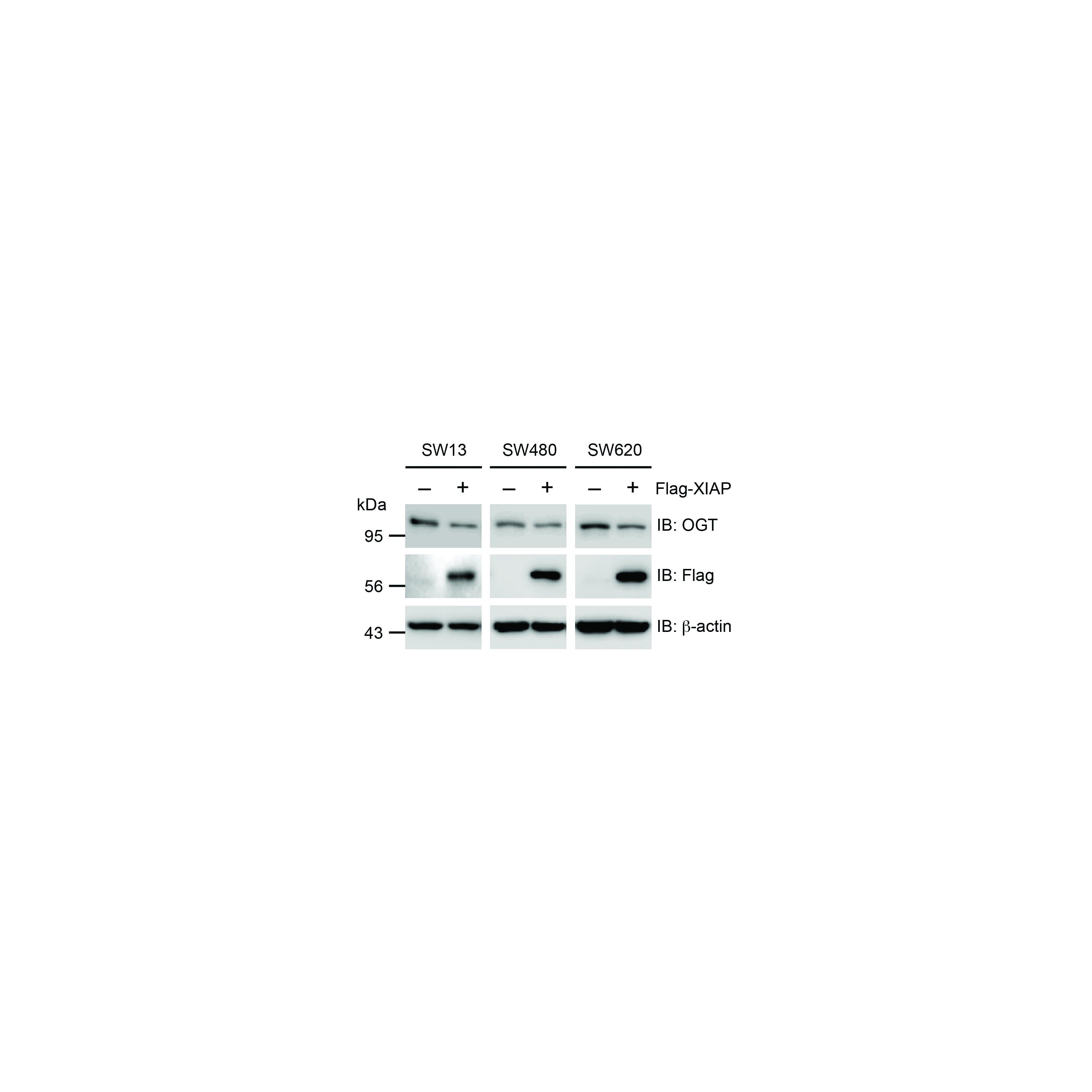

Supplement: Supplementary file 7 — Supplementary Fig. S6 Effects of XIAP overexpression on OGT protein level in several colon cancer cell lines. [file 41419_2020_2999_MOESM7_ESM.tif]
