# Supplementary material for: Mutual regulation between OGT and XIAP to control colon cancer cell growth and invasion
Source: Cell Death Dis. 2020 Sep 29;11(9):815. doi: 10.1038/s41419-020-02999-5 (PMC7525441; doi:10.1038/s41419-020-02999-5)
Supplement: Supplementary file 8 — Supplementary Figure legends (Fig S1-S6) [file 41419_2020_2999_MOESM8_ESM.docx]

**Supplementary Figure Legends**

**Supplementary Fig. S1 Effects of XIAP overexpression on OGT mRNA level.**

**a** Empty or expression vectors encoding Flag-XIAP were transfected into HCT116 cells as indicated. The cells were treated with 20 μM of MG132 or not treated with MG132 as indicated before being harvested. The mRNA levels of OGT were detected by qRT-PCR (*n* = 5 per condition). β-actin was used for normalization.

**b** OGT mRNA levels in HCT116 WT or HCT116 XIAP KO cells were measured by qRT-PCR (*n* = 5 per condition). The values were normalized to β-actin mRNA levels.

The error bars represent as the means ± SD from three independent experiments.

**Supplementary Fig. S2 OGT is a substrate of XIAP and mediates the O-GlcNAcylation of XIAP in HEK293 cells.**

**a** Expression vectors encoding Flag-OGT and HA-Ub were transiently transfected into HEK293 cells overexpressing Myc-XIAP as indicated. The cells were treated with 20 μM of MG132 for 4 h before being harvested. After immunoprecipitation with α-Flag antibodies, the ubiquitination of OGT was analyzed by immunoblotting with α-HA antibodies. The same membrane was re-probed with α-Flag antibodies. Equal amounts of total lysates were subjected to immunoblotting with the indicated antibodies. All data are representative of at least three independent experiments.

**b** Mascot interpretation of O-GlcNAcylated XIAP peptides 406–419 on XIAP in HEK293 cells. Red numbers indicate matches with XIAP peptides.

**Supplementary Fig. S3 The substitution of Serine 406 to alanine and the deletion of the RING domains in XIAP does not affect its interactions with OGT.**

Myc-XIAP WT, XIAP S406A mutant, and XIAP ΔR mutants were transfected into HCT116 cells with transiently expressing Flag-OGT. Cell lysates were immunoprecipitated with α-Flag antibodyies. Co-immunoprecipitated XIAP and loading amounts were analyzed by Western blotting with α-Myc antibodies. Immunoblotting with α-Flag antibodies confirmed that equal amounts of Flag constructs were immunoprecipitated. All data are representative of at least three independent experiments.

**Supplementary Fig. S4 The substitution of Serine 406 to alanine in XIAP does not affect the auto-ubiquitination.**

Expression vectors encoding Flag-XIAP WT, XIAP S406A mutants, XIAP ΔR mutants, and HA-Ub were transfected into HCT116 XIAP KO cells as indicated. After immunoprecipitation with α-Flag antibodies, the ubiquitination of XIAP was analyzed by immunoblotting with α-HA antibodies. The same membrane was re-probed with α-Flag antibodies. Equal amounts of total lysates were subjected to immunoblotting with the indicated antibodies. All data are representative of at least three independent experiments.

**Supplementary Fig. S5 MS analysis for phosphorylation residues on XIAP.**

**a** Summary of phosphorylation sites on XIAP. Expression vectors encoding Flag-OGT were transiently transfected into HEK293 cells and immunoprecipitated Flag-XIAP was subjected to MS analysis.

**b** The CTD MS/MS spectrum of residues 1–29, phosphorylated XIAP peptides, with the doubly charged precursor ion m/z 1231.53 (M+2H)^2+^ is shown.

**c** The CTD MS/MS spectrum of residues 11–29, phosphorylated XIAP peptides, with the doubly charged precursor ion m/z 821.90 (M+2H)^2+^ is shown.

**d** The CTD MS/MS spectrum of residues 335–354, phosphorylated XIAP peptides, with the doubly charged precursor ion m/z 835.32 (M+2H)^2+^ is shown.

**e** The CTD MS/MS spectrum of residues 143–156, phosphorylated XIAP peptides, with the doubly charged precursor ion m/z 823.39 (M+2H)^2+^ is shown.

**f** The CTD MS/MS spectrum of residues 169–182, phosphorylated XIAP peptides, with the doubly charged precursor ion m/z 604.95 (M+2H)^2+^ is shown.

**g** The CTD MS/MS spectrum of residues 169–182, phosphorylated XIAP peptides, with the doubly charged precursor ion m/z 604.93 (M+2H)^2+^ is shown.

**h** The CTD MS/MS spectrum of residues 239–258, phosphorylated XIAP peptides, with the doubly charged precursor ion m/z 759.10 (M+2H)^2+^ is shown.

**i** The CTD MS/MS spectrum of residues 287–299, phosphorylated XIAP peptides, with the doubly charged precursor ion m/z 479.84 (M+2H)^2+^ is shown.

**j** The CTD MS/MS spectrum of residues 406–433, phosphorylated XIAP peptides, with the doubly charged precursor ion m/z 1054.79 (M+2H)^2+^ is shown.

**k** The CTD MS/MS spectrum of residues 420–433, phosphorylated XIAP peptides, with the doubly charged precursor ion m/z 840.86 (M+2H)^2+^ is shown.

**l** The CTD MS/MS spectrum of residues 63–72, phosphorylated XIAP peptides, with the doubly charged precursor ion m/z 709.19 (M+2H)^2+^ is shown.

**m** The CTD MS/MS spectrum of residues 457–472, phosphorylated XIAP peptides, with the doubly charged precursor ion m/z 636.40 (M+2H)^2+^ is shown.

**n** The CTD MS/MS spectrum of residues 473–491, phosphorylated XIAP peptides, with the doubly charged precursor ion m/z 806.18 (M+2H)^2+^ is shown.

**o** The CTD MS/MS spectrum of residues 473–491, phosphorylated XIAP peptides, with the doubly charged precursor ion m/z 826.21 (M+2H)^2+^ is shown.

**p** The CTD MS/MS spectrum of residues 300–322, phosphorylated XIAP peptides, with the doubly charged precursor ion m/z 960.47 (M+2H)^2+^ is shown.

**Supplementary Fig. S6 Effects of XIAP overexpression on OGT protein level in several colon cancer cell lines.**

Empty or expression vectors encoding Flag-XIAP were transfected into several colon carcinoma cell lines, including SW13, SW480, and SW620 as indicated. The expression levels of OGT were analyzed by immunoblotting with α-OGT antibodies. The same membrane was re-probed with α-Flag antibodies. β-actin was used as a loading control.
